# Patterns of Cannabis- and Substance-Related Congenital General Anomalies in Europe: A Geospatiotemporal and Causal Inferential Study

**DOI:** 10.3390/pediatric15010009

**Published:** 2023-02-07

**Authors:** Albert Stuart Reece, Gary Kenneth Hulse

**Affiliations:** 1 Division of Psychiatry, University of Western Australia, Crawley, WA 6009, Australia; 2 School of Medical and Health Sciences, Edith Cowan University, Joondalup, WA 6027, Australia

**Keywords:** tobacco, alcohol, cannabis, cannabinoid, cancer, cancerogenesis, mutagenesis, oncogenesis, genotoxicity, epigenotoxicity, transgenerational inheritance

## Abstract

Introduction: Recent series of congenital anomaly (CA) rates (CARs) have showed the close and epidemiologically causal relationship of cannabis exposure to many CARs. We investigated these trends in Europe where similar trends have occurred. Methods: CARs from EUROCAT. Drug use from European Monitoring Centre for Drugs and Drug Addiction. Income data from World Bank. Results: CARs were higher in countries with increasing daily use overall (*p* = 9.99 × 10^−14^, minimum E-value (mEV) = 2.09) and especially for maternal infections, situs inversus, teratogenic syndromes and VACTERL syndrome (*p* = 1.49 × 10^−15^, mEV = 3.04). In inverse probability weighted panel regression models the series of anomalies: all anomalies, VACTERL, foetal alcohol syndrome, situs inversus (SI), lateralization (L), and teratogenic syndromes (TS; AAVFASSILTS) had cannabis metric *p*-values from: *p* < 2.2 × 10^−16^, 1.52 × 10^−12^, 1.44 × 10^−13^, 1.88 × 10^−7^, 7.39 × 10^−6^ and <2.2 × 10^−16^. In a series of spatiotemporal models this anomaly series had cannabis metric *p*-values from: 8.96 × 10^−6^, 6.56 × 10^−6^, 0.0004, 0.0019, 0.0006, 5.65 × 10^−5^. Considering E-values, the cannabis effect size order was VACTERL > situs inversus > teratogenic syndromes > FAS > lateralization syndromes > all anomalies. 50/64 (78.1%) E-value estimates and 42/64 (65.6%) mEVs > 9. Daily cannabis use was the strongest predictor for all anomalies. Conclusion: Data confirmed laboratory, preclinical and recent epidemiological studies from Canada, Australia, Hawaii, Colorado and USA for teratological links between cannabis exposure and AAVFASSILTS anomalies, fulfilled epidemiological criteria for causality and underscored importance of cannabis teratogenicity. VACTERL data are consistent with causation via cannabis-induced Sonic Hedgehog inhibition. TS data suggest cannabinoid contribution. SI&L data are consistent with results for cardiovascular CAs. Overall, these data show that cannabis is linked across space and time and in a manner which fulfills epidemiological criteria for causality not only with many CAs, but with several multiorgan teratologic syndromes. The major clinical implication of these results is that access to cannabinoids should be tightly restricted in the interests of safeguarding the community’s genetic heritage to protect and preserve coming generations, as is done for all other major genotoxins.

## 1. Introduction

An increasing number of recent reports document the involvement of cannabis in a diverse range of teratological syndromes [1,2,3,4,5,6,7,8,9,10,11,12,13,14,15,16,17,18,18,19,20]. Recent research documents that anomalies in systems as diverse as the cardiovascular, central nervous, chromosomal, gastrointestinal, uronephrolgical, body wall, limb and orofacial systems have been identified as being linked with prenatal or community levels of cannabis exposure [3,7,10,11,13,14,19]. Such a medically systematic approach is obviously normal in considering any environmental toxidrome but it begs the question as to the “other” group of anomalies which do not necessarily fit within the usual systematic approach that medicine generally adopts. Some of the most interesting anomalies of all are in this fascinating and important group of “other” congenital anomalies (CAs).

It is pertinent to observe that cannabinoid teratogenicity is a subset of disorders within the broader class of cannabinoid genotoxicity which also includes cannabinoid-related carcinogenicity and cannabinoid-related cellular aging. These are other closely-related subjects which meaningfully inform the present discussion of cannabinoid teratogenicity.

Recent reports also demonstrate clearly that both Europe and North America are experiencing a triple confluence of, in the first instance, a rising prevalence of cannabis use; secondly, a rising intensity of daily cannabis use; and thirdly, a rising cannabinoid potency for most phytocannabinoids in commercially-available products [21,22,23,23]. This suggests that street-level cannabinoid exposure has increased substantially under the influence of this triple confluence.

This is of major concern given the proliferation of cellular and molecular research demonstrating the exponential dose-response effects of cannabinoid genotoxicity and epigenotoxicity, including cannabis-induced disruptions of mitochondrial and cellular metabolism which directly control chromosomal, genomic and epigenomic processes generally [24,25,26,27,28,29,30,31,32,33,33,34,35]. Moreover, several recent epidemiological studies have verified the predicted abrupt jump in anomaly rates observed following the highest levels of cannabis exposure [2,7,8,14]. Thus, community epidemiological data confirms laboratory findings of exponential cannabis effects [24,25,26,27,28,29,30,31,32,33,33,34,35]. This in turn implies that due to the relatively sudden rises in cannabis use prevalence, intensity and potency, a relatively abrupt rise in major teratologic, mutagenic and genotoxic presentations may be expected as major public health outcomes. Indeed, there are already indications of similar abrupt increases in anomaly rates both in the USA and Europe [21,22,23,23].

A group of eleven anomalies including all anomalies, conjoint twins, foetal alcohol syndrome (FAS), skeletal dysplasias, teratogenic syndromes, amniotic bands, lateralization anomalies, maternal infections causing malformations, situs inversus, valproate syndrome, and VACTERL syndrome were studied. While the group of “all anomalies” is evidently of crucial interest, a number of anomalies provided significant insight into the effects of cannabis exposure. VACTERL syndrome is an acronym for vertebral defects, anorectal anomalies, cardiac defects, tracheoesophageal fistula/oesophageal atresia, renal anomalies and limb anomalies [36]. It was recently shown to be due to inhibition of one of the major embryonic morphogens, sonic hedgehog, in a manner which could be induced by the cannabinoids Δ9-tetrahydrocannabinol (THC) and cannabidiol both directly [34] and via epigenomic pathways [37]. Moreover, it seemed to contain within just one syndrome most of the multisystem disorders which have recently been attributed to cannabis exposure. Furthermore, a demonstrated causal relationship with the multisystem VACTERL syndrome would completely belie the typically harmless characterization of cannabis in popular culture. Given that the cluster of “teratogenic syndromes” were clearly in need of some aetiopathological explanation, and given the diverse nature of cannabinoid teratology, we wanted to learn if perhaps some of the variance seen in this disorder might potentially be epidemiologically explained by cannabinoid exposure. Foetal alcohol syndrome (FAS) has been described as being caused in part by epigenomic signalling through the cannabinoid type 1 receptor (CB1R). Moreover, cannabis and alcohol are often co-abused and each has been reported as performing a gateway role for the other [38,39,40,41,42,43,44,45,46]. This made the outcome of this analysis of great interest. Cannabis has recently been reported as being associated with many cardiovascular anomalies, including transposition of the great arteries [7,14,47], which can be considered as a limited cardiovascular manifestation of lateralization syndromes or situs inversus. Taken together, these disorders would argue for a general disruption of left-right lateralization mechanisms generally throughout body morphogenesis.

Many pathways have been described implicating several cannabinoids in genotoxic outcomes, including grossly abnormal sperm morphology [48,49], disruption of oocyte division [50], single- and double-stranded DNA breaks [51,52], chromosomal translocations and anomalous end-to-end joining [48], chromosomal bridge formation [33,50,53,54], micronucleus formation [27,30,55,56,57,58,59,60,61,62,63,64], oxidation of the bases of DNA [33], reduced DNA, RNA and protein synthesis [65,66,67,68,69], reduced histone synthesis [66,67,70,71,72] including post-translational modifications of histones [66,67,70,71,72], disordered oviduct function [73], disrupted sperm motility [48,49], grossly altered DNA methylation [37,74,75,76,77,78,79,80,81] which was shown to be inheritable via sperm [37,80,81], and heritable alterations of histone patterns [72]. Moreover, mitochondrial metabolism, on which many genomic and epigenomic reactions are based, has also been well-demonstrated to be grossly disrupted by several mechanisms [73,82,83,84,85,86,87,88], including reduction of synthesis of many cytochromes of the electron transport chain and, indeed, the critical F1-ATPase itself [88].

This study was performed to assess if any of the CA disorders in this group demonstrated a relationship to metrics of cannabis exposure, survived multivariable adjustment, and if so, whether this relationship fulfilled quantitative epidemiological criteria for causality. As a result of the wider links between many of these disorders and other syndromes, the present analysis has implications stretching beyond merely the CAs listed herein, and indeed, contributes pointedly to a generic consideration of cannabinoid genotoxicity more broadly.

## 2. Methods

Data for analysis was downloaded from the European Network of Population-Based Registries for the Epidemiological Surveillance of Congenital Anomalies (EUROCAT) website [89]. Data on all available congenital anomaly rates was sourced by each individual year for each of 14 nations. The total congenital anomaly rate in the EUROCAT data comprehends anomaly rates amongst live births, stillbirths, and cases where early termination for anomaly was practised, all combined together. The total congenital anomaly rate therefore represents a total overall picture across all classes of births. Nations were selected based on the availability of their congenital anomaly data for the years 2010–2019. The World Health Organization [90] was the source of national tobacco (percent daily tobacco use prevalence) and alcohol (litres of pure alcohol consumed per capita annually) use data. Drug use data was sourced from the European Monitoring Centre for Drugs and Drug Addiction (EMCDDA) [91] for cannabis, amphetamines and cocaine. In addition to this, data cannabis consumption data was supplemented by data on the tetrahydrocannabinol (THC) content of cannabis herb and resin published in recent reports [22]. This data was originally sourced from EMCDDA and was therefore coincident with EMCDDA data for this data field [22]. The World Bank [92] was the source of median household income data (in USD).

Nations were categorized as being either low and/or falling daily cannabis use, or high and/or rising daily cannabis use, based on a recent European epidemiological study (see Supplementary Figure S4 [22]). Thus Belgium, Italy, The Netherlands, Norway, Portugal, Croatia, France, Germany and Spain were categorized as nations experiencing increasing daily use, while Poland, Bulgaria, Finland, Hungary and Sweden were nations which were experiencing low or falling levels of daily cannabis use.

Various derived metrics of cannabis exposure could be calculated from the several metrics of cannabis use available. In this way, last month cannabis use prevalence data was multiplied by the THC content of cannabis herb and resin to derive compound metrics. These metrics were then also multiplied by imputed daily cannabis use prevalence rates to derive further compound metrics both for cannabis herb and cannabis resin.

Missing data was completed by linear interpolation. This technique was particularly employed for daily cannabis use. A total of 59 data points on daily cannabis use were available from EMCDDA for these 14 nations over this period. Linear interpolation was used to expand this dataset to 129 datapoints (further details provided in Section 3). Whilst data on cannabis resin THC concentration were not available for Sweden, it was noted that the resin to herb THC concentration was almost constant in nearby Norway at 17.7, so this ratio was therefore applied to the Swedish cannabis herb THC concentration data to derive estimates of Swedish cannabis resin THC concentration. Similarly, data for the cannabis resin THC concentration in Poland were unavailable. The resin to herb THC concentration ratio of neighbouring Germany was used to estimate the resin THC content in Poland from the Polish herb THC concentrations which were known. Since geospatial analytical techniques do not tolerate missing data, the dataset was completed by the last observation carried forward or backwards for The Netherlands in 2010 and for Croatia in 2018 and 2019. Multiple imputation methods could not be used for this analysis as multiple datasets cannot be inputted in panel or spatial multivariable regression techniques.

RStudio version 1.4.1717, based on R version 4.1.1 from the Comprehensive R Archive Network and the R Foundation for Statistical Computing [93], was used for data processing. The analysis was performed in December 2021. dplyr from the tidyverse [94] was used for data manipulation. The results of the Shapiro-Wilk test were used to guide the decision on whether to log transform data in order to improve compliance with normality assumptions. ggplot2 from tidyverse was used to draw graphs. ggplot2 and sf (simple features) [95] were used to draw maps and both custom colour palettes and palettes taken from the viridis and viridisLite packages were used to generate colour fill panels [96]. The package colorplaner [97] was used to draw bivariate maps. All illustrations are original. They have not been published previously. Linear regression was performed in Base R. Mixed effects regression was conducted using R package nlme [98]. In all multivariable models the classical technique of model reduction was employed using serial deletion of the least significant term to yield a final reduced model which is the model presented. Multiple linear models were processed in a single pass using nested and combined techniques from R packages purrr and broom [94,99,100]. The overall effect of covariates in multivariable models may be quantified and is known as the marginal effect. In this study, the overall marginal effect was calculated using the R package margins [101].

The presence of multiple different metrics for cannabis consumption and exposure created an important problem of covariate redundancy for analysis as it was not clear which was the most appropriate metric to employ for any particular model. Use of excessive covariates in a multivariable model would unnecessarily consume degrees of freedom and lead to problems of collinearity and thereby restrict ability to assess interactions. This issue was addressed formally by the use of random forest regression using the R package ranger [102], with variable importance being quantified via the R package vip (variable importance plot) [103]. This process was used to select the most predictive covariates which were entered into the regression modelling equations. The tables from this analysis are presented in the Section 3.

Panel analysis was conducted using R package plm [104] across both space and time simultaneously for which the “twoways” effect was employed. The spatial weights matrix was computed using the edge and corner “queen” relationships using R package spdep (spatial dependency) [105]. Geospatial modelling was conducted using the spatial panel random effects maximum likelihood (spreml) function from the package spml, which allows modelling and correction of model error structures in a detailed manner [106,107]. Such models may produce four model coefficients of interest, which can then be used to determine the most appropriate error structure for the model. These coefficients are rho, the spatial coefficient; phi, the random error effect; psi, the serial correlation effect; and theta, the spatial autocorrelation coefficient. In this manner, the most appropriate error structure was chosen for each spatial model, generally, taking care to preserve the model error specification across closely-related models. The appropriate error structure was determined by the backwards methods from the full general model to the most specific model, as has been published previously [108]. Both panel and geospatial models were temporally lagged as shown by one to two years.

The tools of formal causal inference were employed in this analysis. Inverse probability weighting (ipw) is the technique of choice to convert a purely observational study into a pseudo-randomized study and from such analyses, it is appropriate to draw causal inferences [109]. All of the multivariable panel models presented herein were inverse probability weighted. Inverse probability weighting was performed using the R package ipw. Similarly, E-values (expected values) quantify the correlation required of some hypothetical unmeasured confounder covariate with both the outcome of interest and the exposure of concern in order to explain away some apparently causal relationship [110,111,112]. It is thus an important technique of sensitivity analysis and therefore provides a quantitative measure of the robustness of the model to extraneous covariates which have not been accounted for within the measured parameters. E-values have a confidence interval associated with them and the 95% lower bound of this confidence interval is reported in the present study. E-value estimates greater than 1.25 are indicative of causality [113], with E-values greater than 9 described as being in the high range [114]. E-values were calculated from the R package EValue [115]. Both inverse probability weighting and E-values are foundational and pivotal techniques used in formal causal inferential methods in order to allow causal relationships to be assessed from real-world observational studies and together create a powerful causal inferential pseudo-randomized analytical paradigm.

## 3. Results

Presentation of Results in this section would be aided by a Navigation chart to assist the reader to properly understand the analysis plan which has been undertaken. This may be set out as follows: 

### 3.1. Data Presentation

An overall profile of the 14 countries contributing data and the 11 congenital anomalies investigated is shown in Supplementary Table S1. The table also provides national substance use exposure, including compound variables for cannabis exposure, and median household income.

Supplementary Table S2 provides the available data on daily cannabis use for countries over the applicable time period. It is notably incomplete. For these reasons, data was supplemented by linear interpolation with the addition of a further 70 data points to obtain the dataset shown in Supplementary Table S3.

### 3.2. Bivariate Analysis

#### 3.2.1. Continuous Data

**Figure 1:**
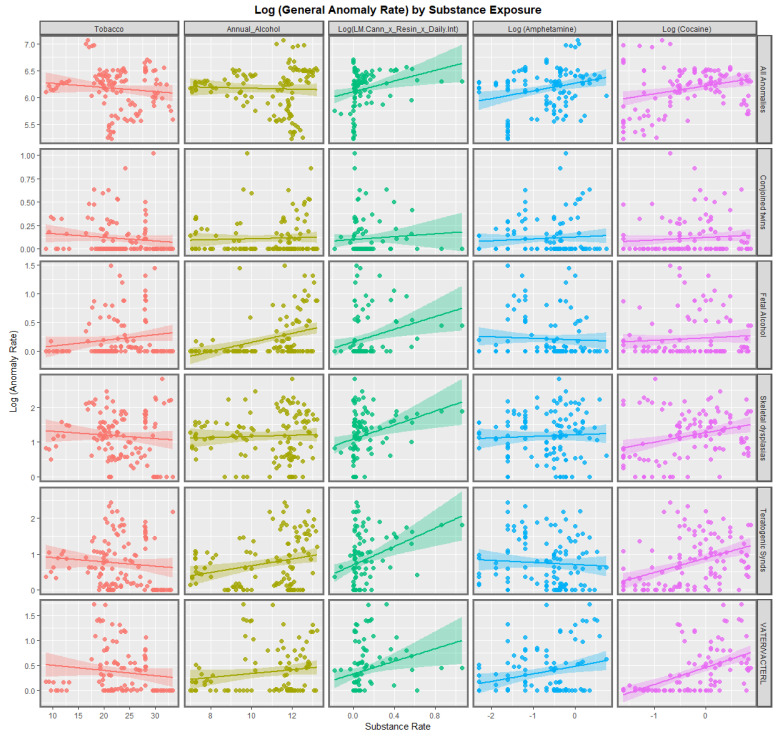
Log (Congenital Anomaly Rates) by substance exposures. Panelled scatterplots of log (rates of selected congenital anomalies) against various substance exposures.

**Figure 2:**
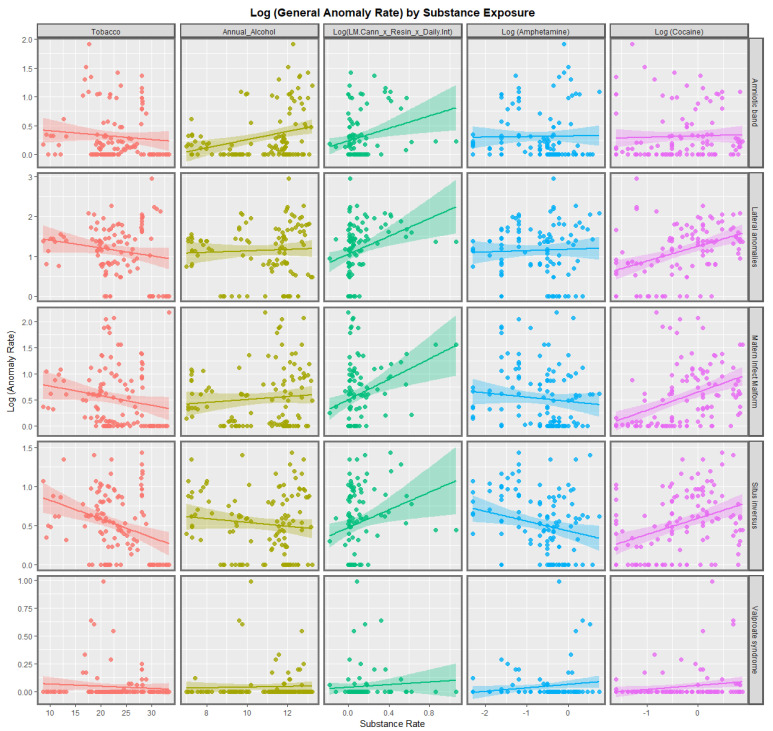
Log (Congenital Anomaly Rates) by substance exposures. Panelled scatterplots of log (rates of selected congenital anomalies) against various substance exposures. The set of congenital anomalies illustrated in this Figure is different to those chosen in Figure 1.

Figure 1 and Figure 2 show the bivariate relationship between substance exposure and the rates of the various anomalies in this class. It is interesting that tobacco use is negatively associated with all the anomalies except fetal alcohol syndrome (FAS), which is unsurprising as tobacco and alcohol use are often co-associated. The trend lines for alcohol use are mostly flat although they are strongly positive for FAS, teratogenic syndromes and amniotic bands. For amphetamine exposure, the trend lines are mostly flat or negative but are significantly positive for all anomalies and VACTERL syndrome. Seven trend lines for cocaine exposure are strongly positive as indicated.

The cannabis use metrics employed in these graphs is the compound metric of last month daily use × cannabis resin THC concentration × daily use interpolated. For ten of the eleven anomalies listed the trend line for this cannabis exposure metric is more strongly positive than any of the other trend lines in these two graphs. From visual inspection it is likely that the exceptional anomaly is conjoined twins.

**Figure 3:**
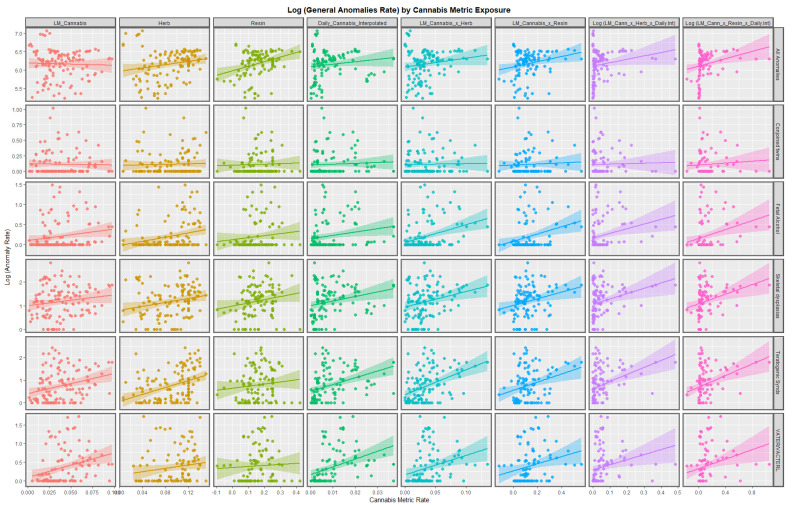
Log (Congenital Anomaly Rates) by cannabis metrics. Panelled scatterplots of log (rates of selected congenital anomalies) against various metrics of cannabis exposure.

**Figure 4:**
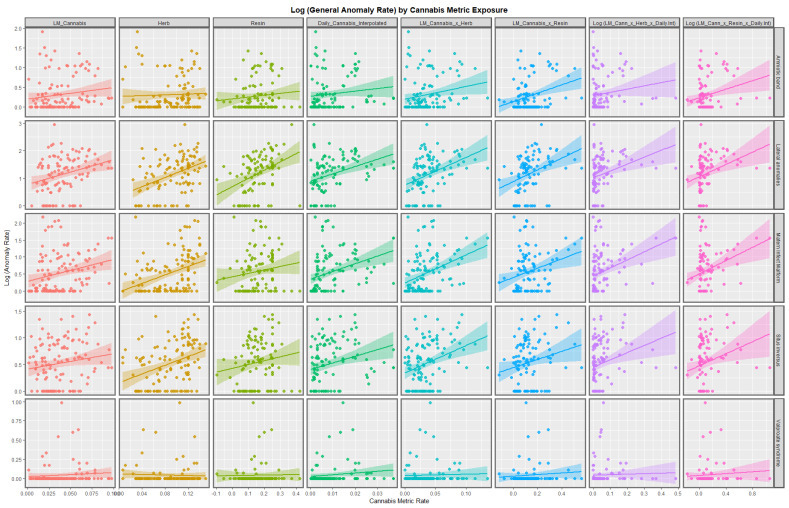
Log (Congenital Anomaly Rates) by cannabis metrics. Panelled scatterplots of log (rates of selected congenital anomalies) against various metrics of cannabis exposure. This Figure illustrates a different set of congenital anomalies additional to those shown in Figure 3.

Figure 3 and Figure 4 shown the bivariate relationship between the various anomalies and different cannabis exposure metrics. In stark contrast to the preceding figures, many of the cannabis exposure metrics appear to be strongly related to this set of anomalies, especially for the compound metrics on the right hand side of the graph. 

**Figure 5:**
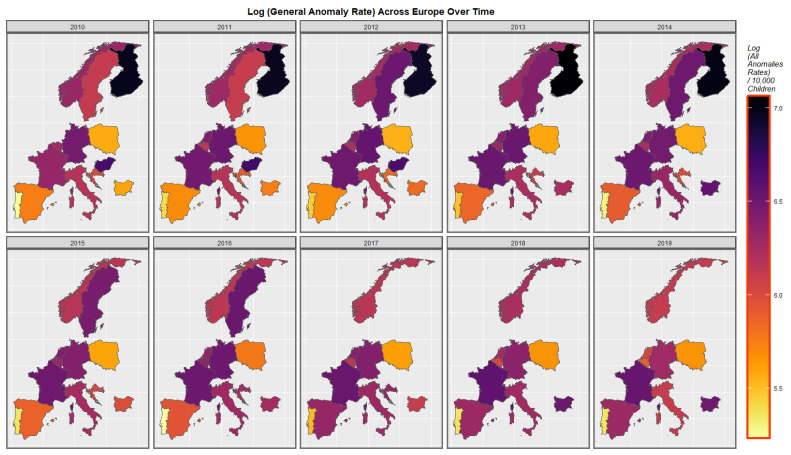
Log (All General Anomaly Rate) in selected European nations. Sequential map-graph of the log rate of all general anomalies over time in selected European nations.

The distribution of the group “All Anomalies” across space and time over the decade in Europe is shown in Figure 5. The situation in France, Spain, Bulgaria, Poland and Sweden has deteriorated whilst that in Germany appears to have improved. The rates in Belgium, The Netherlands and Portugal has varied across this period.

Supplementary Figure S1 reveals that the rates of FAS across space and time have varied in a patchy manner.

Supplementary Figure S2 charts the space-time occurrence of skeletal dysplasia. The largest consistent change on this map is the rise in the rates of this anomaly in Spain. The Netherlands, Belgium and Bulgaria also reported high rates at times.

**Figure 6:**
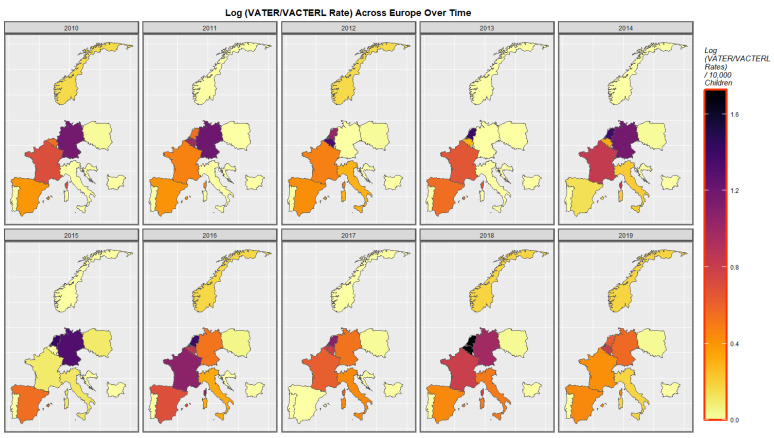
Log (VACTERL Rates) in selected European nations. Sequential map-graph of the log rate of VACTERL anomalies over time in selected European nations.

The rates across Europe of VACTERL syndrome are shown in Figure 6. Very high rates prominently stand out in Belgium and The Netherlands in some years, which is more prominent when it is noted that this is actually a plot of the logarithm of the rates, so the differences for the raw data are even greater.

**Figure 7:**
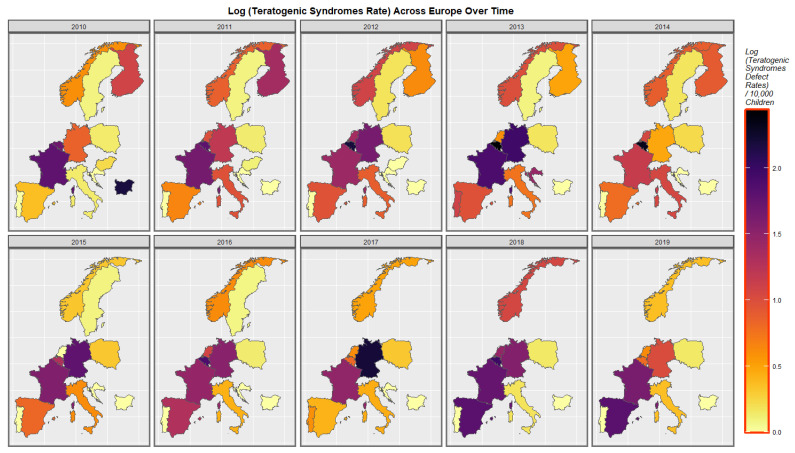
Log (Teratogenic Syndrome Rates) in selected European nations. Sequential map-graph of the log rate of teratogenic syndromes over time in selected European nations.

The rates of teratogenic syndromes in Europe are shown in Figure 7. Belgium often reports very high rates with Germany and Bulgaria reporting high rates in some years. The rates in France, Germany and Norway seem to fluctuate significantly over this decade.

Supplementary Figure S3 illustrates the rates of the compound cannabis index last month cannabis use × cannabis resin THC concentration × daily cannabis use interpolated. It is apparent that this index has increased in all nations across the continent over this time with particularly marked increases in France and Spain, but The Netherlands, Poland, Portugal, Norway and Croatia also reporting higher rates.

**Figure 8:**
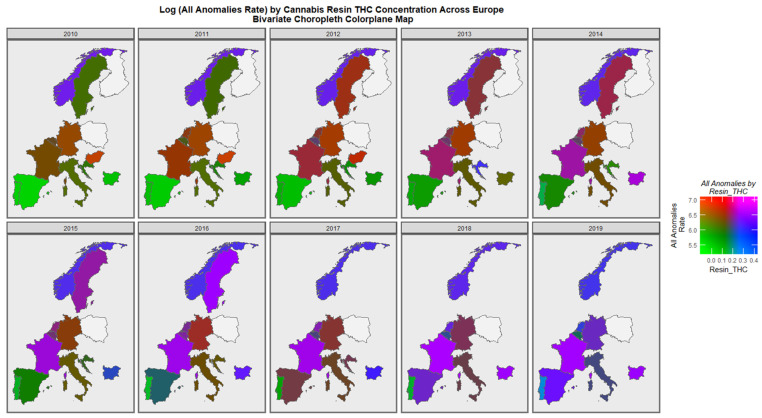
Bivariate graph of All General Anomalies by Cannabis Resin THC Concentration. Bivariate colorplane sequential map-graph of the log rate of all anomalies by cannabis resin THC concentration in selected European nations. See text for interpretation.

Figure 8 is a bivariate colorplane map showing the association between the rate of all anomalies and the cannabis resin THC concentration. As shown in the colorplane key, green shading is indicative of low rates of both covariates, whilst purple and pink shading shows that both rates are high. Other colours have meanings as shown in the colorplane key. Therefore, the increase in both covariates is clearly demonstrated for France, Bulgaria, Spain, Germany and Sweden, which move into violet and purple shades.

Supplementary Figure S4 shows the space-time co-distribution of FAS and cannabis use × resin THC concentration × daily interpolated use. France is noted to have experienced high rates of both covariates in 2014, 2015 and 2018.

**Figure 9:**
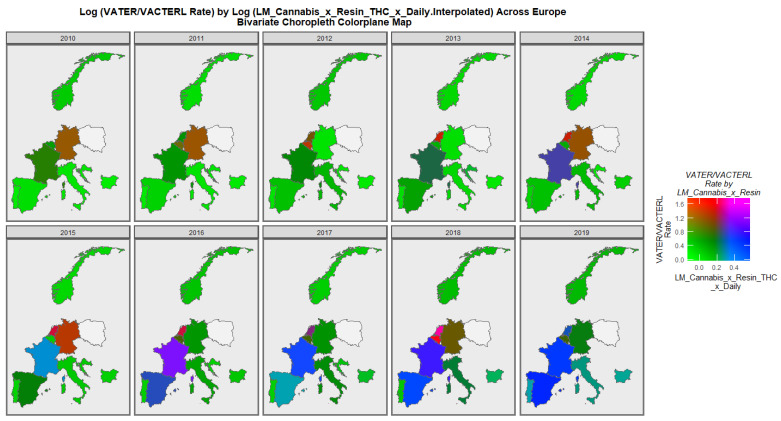
Bivariate graph of VACTERL syndrome by last month cannabis use. Bivariate colorplane sequential map-graph of the log rate of VACTERL anomalies by last month cannabis use: cannabis resin THC concentration: daily cannabis use interpolated in selected European nations.

The co-varying patterns of VACTERL syndrome and cannabis use × resin THC concentration are shown in Figure 9. France is noted to have had high rates in 2016, and Belgium and The Netherlands are both noted to have reported high rates of these covariates in 2018.

**Figure 10:**
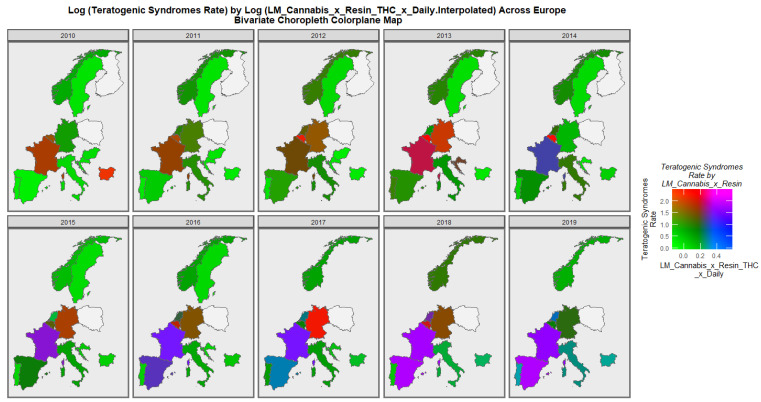
Bivariate graph of teratogenic syndromes by last month cannabis use. Bivariate colorplane sequential map-graph of the log rate of teratogenic syndromes by last month cannabis use: cannabis resin THC concentration: daily cannabis use interpolated in selected European nations.

The spatiotemporal patterns of teratogenic syndromes across Europe is shown in Figure 10. There is a prominent emergence of high rates of both covariates in France, Spain, and The Netherlands over the decade of observation.

From Supplementary Figure S5 it emerges that high rates of the covariates skeletal dysplasia and cannabis use × resin THC concentration × daily interpolated emerged in France and Spain across this period.

Interestingly, when the covariates maternal infections and cannabis use × resin THC concentration × daily interpolated are considered as in Supplementary Figure S6, one again notes the emergence of high rates of these covariates together in France and Spain.

#### 3.2.2. Categorical Bivariate Analysis

**Figure 11:**
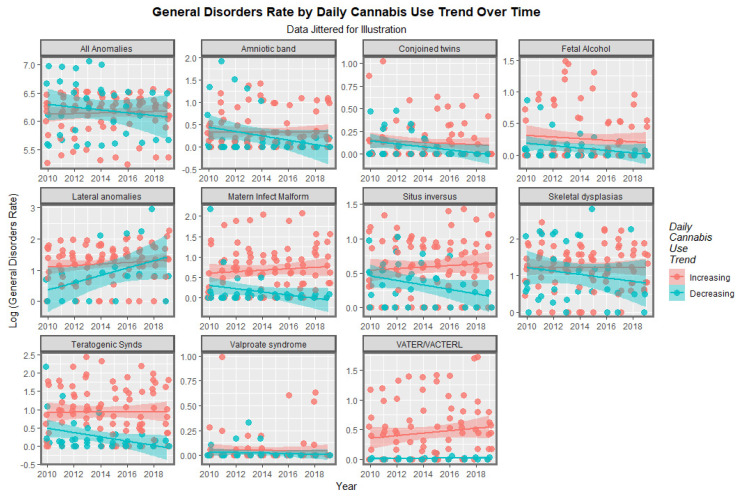
Log rate of General Disorders Over Time, by disorder. Panelled scatterplots of the rate of various anomalies in nations with increasing daily cannabis use compared to those which do not, by congenital anomaly.

Nations may be divided into those with high rates of daily use or lower or decreasing rates of daily cannabis use, based on recent epidemiological analyses of these trends [22]. When the rates of these CAs across the continent are compared, it is clear that for some CAs the rates in those nations with increasing rates of daily cannabis use are higher than those where daily use is less prominent (Figure 11). At mixed effects regression, using the CA as the random effect, these differences were highly significant (β-est. = 0.2223, t = 7.52, *p* = 9.99 × 10^−14^; model AIC = 1876.46, Log.Lik = −923.873; minimum E-value = 2.09). When consideration is limited to the four variables where this effect was most obvious (from Figure 11, namely maternal infections, situs inversus, teratogenic syndromes and VACTERL syndrome) the effects was more pronounced still (β-est. = 1.4923, t = 8.26, *p* = 1.49 × 10^−15^; model AIC = 718.878, Log.Lik = −355.439; minimum E-value = 3.05).

**Figure 12:**
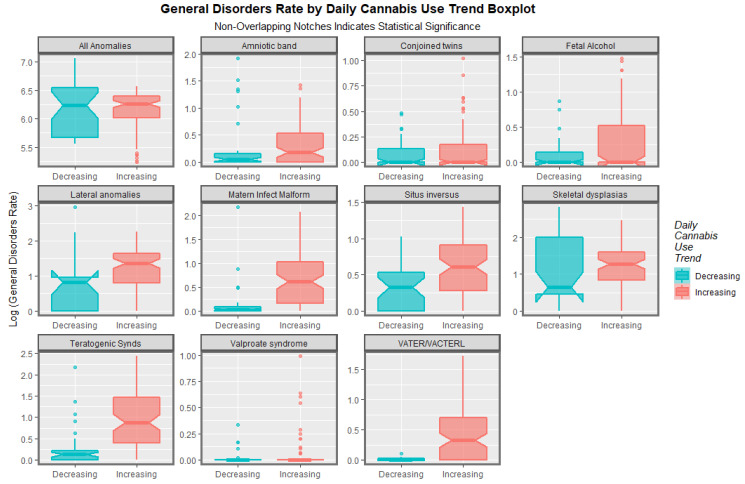
Time-aggregated boxplots of log rate of General Disorders Over Time, by Disorder. Panelled boxplots of the time-aggregated rate of various anomalies in nations with increasing daily cannabis use compared to those which do not, by congenital anomaly.

These effects may be aggregated in time and the relative rates compared. This has been done in Figure 12 where box plots convey this information graphically. The areas where the notches do not overlap indicate statistically significant differences graphically. These differences in the aggregated rates are shown quantitatively in Table 1 along with the relative rates in the countries with increasing daily use compared to that in those where it is declining, along with *t*-tests of statistical significance. The table is ordered in order of declining values of Student’s *t*. It is interesting to note that the table is headed by the VACTERL syndrome, followed by teratogenic syndromes. Situs inversus is also highly significant. FAS is also significantly higher in nations with increasing daily cannabis use.

The slopes of 132 of the regression lines from Figure 1 and Figure 2 are shown in Supplementary Table S4. Those 66 terms with positive and statistically significant regression coefficients are extracted and shown in Table 2. The terms are listed in descending order of minimum E-value (mEV). It is of interest that 55 (83.3%) of the remaining terms include various cannabis metrics, 8 (12.1%) include cocaine and 3 (4.5%) include alcohol. The top rows of this table shows that six of the first nine terms include interpolated daily cannabis exposure.

### 3.3. Multivariate Analysis

#### 3.3.1. Panel Regression

Having demonstrated these important and powerful bivariate relationships, the next issue to arise was how they compared in a multivariable context. However, given the very large number of covariates concerned, including the high number of covariates for the various cannabis metrics, the issue of the choice of the best covariates to enter into multivariable regression equations is non-trivial. This issue was formally addressed by using random Forrest regression (from the R Package ranger) in tandem with variable importance plots (from R package vip). This gave rise to the Random Forrest Variable Importance tables shown in Supplementary Tables S5–S10.

As suggested in these tables, it was decided to focus the analysis going forward on the six CAs described in these tables, namely All anomalies, VACTERL syndrome, FAS, situs inversus, lateralization anomalies and teratogenic syndromes. The rationale for selection of these six CAs is explained in the Section 4.

Supplementary Table S11 sets out the results of inverse probability weighted multivariable panel regression in additive and interactive models and in models lagged by one and two years. Inverse probability weighting is an important technical modification to usual panel regression which allows us to generalize our results from the merely observational context into a more generalizable scenario through the adoption of pseudo-randomization via the weighting procedure. In each case, terms including cannabis exposure remain in the final model, have positive coefficients and are highly statistically significant. Indeed, in the two lagged models several terms positive and significant for cannabis are reported.

Similar findings appear in Supplementary Table S12 for VACTERL syndrome. In this case however, the signal for cannabis terms disappears at two years of temporal lag but re-appears at four years of temporal lag as indicated.

Since the cause of VACTERL syndrome was not elucidated until recently, it is of interest to formally compare it to a similar model which omits cannabis terms. This additive model is shown in the last section of Supplementary Table S12. Unfortunately, it is not possible to directly compare panel models with, for example, an ANOVA test, as would usually be done for many other model types. However, it is noted that when moving from the additive model without cannabis terms to the additive model which includes cannabis terms, the degree of the data variance which is accounted for by the model (indicated by R-squared) increases from 28.29% to 39.46% and the significance of the model *p*-value greatly increases from *p* = 4.51 × 10^−3^ to *p* = 4.45 × 10^−14^.

Supplementary Tables S13–S16 perform similar functions for FAS, situs inversus. Lateralization syndromes and teratogenic syndromes all include the important inverse probability weighting. Similar multivariable findings are made as those noted above.

In the case of teratogenic syndromes reported in Supplementary Table S16 it is of great interest to consider the potential contribution of cannabis to this syndrome which is clearly not well understood. Once again, a model which excludes cannabis terms in included at the foot of this table with details as shown. It is noted that compared to the model without cannabis-related terms, the amount of variance accounted for by the model greatly increases from 30.41% to 40.89% and the significance of the model *p*-value rises by 19 orders of magnitude, from *p* = 1.32 × 10^−7^ to 1.61 × 10^−26^.

#### 3.3.2. Geospatial Analysis

It was of interest to consider these data in their native space-time context in order to formally account for analytically-important confounding factors including random error effects, serial correlation, spatial correlation and spatial autocorrelation. For this purpose, geospatial links between the countries were derived, edited and finalized as shown in Supplementary Figure S7, which formed the basis of the sparse spatial weights matrix for the implementation of formal geospatial regression.

Table 3 presents the results of geospatial regression in additive and interactive models and in models lagged to two years. In all three models, terms including cannabis use remain in the final model, are positive and are collectively highly statistically significant. Indeed, it is noted that in the interactive model, only cannabis terms remain in the final model. From comparing the magnitude of the regression coefficients (as they all sum to greater than zero), it is clear that the overall effect of cannabis metrics in these models is strongly in the positive direction.

When the VACTERL syndrome is considered (Table 4), similar findings are made. Again, many terms positive for cannabis remain in final models and the overall effect of cannabis on these models is clearly in the positive direction. In this case, models lagged to four and six years are also included and these effects continue to become even stronger with lagged time.

With VACTERL, it is again of interest to compare these spatial models to those without cannabis terms included. This is presented in Table 5. Here the Log of the maximum likelihood ratio at model optimization (LogLik.) increases from −28.449 without cannabis terms in the additive model to −26.104 when they are included. Spatial models may be directly compared using the spatial Hausman test. In this case, the model with cannabis terms included is superior (Chi Squ. = 8.12, df = 3, *p* = 0.0436). When the model lagged by two years without cannabis terms is compared to the similar model with them included the LogLik. Ratio increases from −39.6349 to −22.2103 (Spatial Hausman test ChiSqu. = 82.41, df = 3, *p* = 9.33 × 10^−18^). Hence, the models which include cannabis terms are shown to be clearly analytically greatly superior to those without such terms.

Similar findings apply to the spatial analysis of FAS, situs inversus, lateralization syndromes and teratogenic syndromes presented in Table 6, Table 7, Table 8 and Table 9.

For the teratogenic syndromes, which are clearly of unknown aetiology, it is of interest to formally consider the potential difference made by the inclusion of cannabis metrics. Table 10 presents the model without cannabis terms and as noted there, the model which includes cannabis is greatly and significantly statistically superior (LogLik. increases from −106.99 to −63.74, Spatial Hausman test: ChiSqu. = 184.20, df = 2, *p* = 1.00 × 10^−40^).

### 3.4. Causal Inference

#### E-Values

E-values or expected values can be calculated from all of the various multivariable regression models presented. These are listed for panel models and for geospatial models in Table 11 and Table 12 respectively. It is of considerable interest and importance that four of the E-value estimates for VACTERL syndrome in spatial models are infinite ad are two of the mEV values. These two tables are combined and listed in descending order of the mEV in Supplementary Table S17. It is significant here that the VACTERL syndrome, spatial regression formats and daily cannabis use occupy the first four rows on this list.

These 64 E-value pairs are then listed consecutively in descending order in Table 13. 50/64 (78.1%) E-value estimates exceed 9 and are therefore considered to be in the high range [114], and all 64 (100%) exceed the threshold for causality at 1.25 [113]. For the minimum E-value estimates, 42/64 (65.6%) exceed 9 and are thus in the high range and 62/64 (96.9%) exceed the threshold for causality at 1.25.

Table 14 re-lists Supplementary Table S17 in order of CAs so that they can be compared directly. Summary measures of the E-value estimate and the mEV are presented in Table 15 and listed in descending order of mEV and E-value estimate. It is important to note that the list is headed by VACTERL syndrome and teratogenic syndromes, and FAS also scores highly in this table.

Table 14 is re-listed in order of the substance exposure term in Table 16 and the exposures are grouped into the primary exposure of interest being daily cannabis use interpolated, cannabis herb THC concentration of cannabis resin THC concentration. Grouped summary data for these E-values are then presented in Table 17 and again ordered by descending E-value. It is noted in this table that the order here is daily cannabis use > cannabis herb > cannabis resin.

These groups are formally compared using the Wilcoxson test in Table 18 and all the apparent differences noted in Table 17 are found to be highly statistically significant at the levels indicated in Table 18.

## 4. Discussion

### 4.1. Main Results

A strong positive relationship was shown in bivariate analysis between CARs and many metrics of cannabis exposure. The compound metric last month cannabis use × cannabis resin THC concentration × daily cannabis use interpolated was a powerful predictor of CAR (Figure 3 and Figure 4), just as was shown earlier [7]. Strong upward trends between cannabis resin THC concentration and all anomalies, skeletal dysplasia, lateral anomalies, maternal infections and situs inversus were noted.

Consideration of the maps showed that for VACTERL syndrome, there had been a dramatic rise in low countries and France. Teratogenic syndromes were also often high in low countries. Bivariate maps showed that the rates of the following anomalies increased along with cannabis herb THC concentration in France and Spain—All anomalies, FAS, VACTERL, Teratogenic syndromes, skeletal anomalies and maternal infections.

CARs were shown to be higher in countries with increasing daily use overall (*p* = 9.99 × 10^−14^, mEV = 2.09) and especially for maternal infections, situs inversus, teratogenic syndromes and VACTERL syndrome (*p* = 1.49 × 10^−15^, mEV = 3.04). For VACTERL and teratogenic syndromes, the relative rates in countries with increasing daily use were 31.82 and 3.83 times higher than other nations (*p* = 7.49 × 10^−13^ and 1.81 × 10^−8^ respectively).

In inverse probability weighted panel regression models, the series of anomalies: all anomalies, VACTERL, foetal alcohol syndrome, situs inversus (SI) lateralization (L) and teratogenic syndromes had *p*-values for cannabis metrics from: *p* < 2.2 × 10^−16^, 1.52 × 10^−12^, 1.44 × 10^−13^, 1.88 × 10^−7^, 7.39 × 10^−6^ and <2.2 × 10^−16^. In a series of spatiotemporal models, the same anomaly series had *p*-values for cannabis metrics from: 8.96 × 10^−6^, 6.56 × 10^−6^, 0.0004, 0.0019, 0.0006, 5.65 × 10^−5^.

Comparison of geospatial VACTERL models with and without cannabis metrics showed that models including terms for cannabis were greatly superior (at 2 Lags, *p* < 2.2 × 10^−16^). Comparison of geospatial models for teratogenic syndromes with and without cannabis found similarly (additive model *p* < 2.2 × 10^−16^).

A total of 50/64 (78.1%) E-value estimates and 42/64 (65.6%) minimum E-values exceeded 9 and so were in the high risk zone [114]. All 64 E-value estimates and 62/64 lower E-values exceeded 1.25 and thus achieve the threshold for causal inference [113]. Considering E-values, the rate of effect size from cannabis was VACTERL > situs inversus > teratogenic syndromes > FAS > lateralization syndromes > all anomalies. Daily cannabis use interpolated the strongest predictor for all anomalies.

### 4.2. Choice of Anomalies

This group of anomalies was made up from CAs which did not fit in the standard organ-specific systems such as cardiovascular system, etc. Some of the anomalies which were chosen for more detailed study were chosen due to high effect size on the bivariate plots in Figure 1, Figure 2, Figure 3 and Figure 4 (situs inversus, lateralization syndromes, foetal alcohol syndrome). All anomalies was chosen for its obvious overall importance to the field.

Some syndromes were chosen because they are known to have pathophysiological overlap with cannabinoid effects, such as foetal alcohol syndrome, which has been shown to signal to the epigenome through the cannabinoid type 1 receptor (CB1R) [116,117,118,119,120,121,122,123,124,125,126,127,128,129,130]. Moreover, cannabis is often co-abused with alcohol and each has been described as being a gateway drug for the other [38,40,41,42,43,44,45,46].

VACTERL syndrome was chosen as it had recently been described as being related to Sonic Hedgehog inhibition, a change which was inducible by both THC and cannabidiol [34]. Epigenetic modulation of the Sonic Hedgehog pathway in cannabis withdrawal has also been demonstrated [37]. This group identified differentially-methylated genes at BMP4 (bone morphogenetic protein 4), GLI3 (Gli family zinc finger 3), MEGF8 (multiple EGF-like domains 8), and TMEM107 (transmembrane protein 107) and CHD7 (chromodomain helicase DNA binding protein 7) ([37] Supplementary Material Pages 352, 354, 356, *p* = 0.00547). The first four of these are all either part of the Sonic Hedgehog signalling pathway or are modifiers of it. Hence, we wished to test the epidemiological evidence for cannabis involvement in this syndrome.

Increased evidence of cannabinoid association or causation in VACTERL syndrome was also of great interest as it comprised within one syndrome most of the spectrum of congenital anomalies which have recently been connected to community or prenatal cannabis exposure. Hence, a demonstrated result here would prove the whole severity and broad spectrum which is increasingly being outlined within cannabis teratology. VACTERL syndrome was also of interest as it is by definition multisystem and polysyndromic. A positive result in causal inferential and space-time analysis would directly implicate cannabis in multisystem disease and disprove the recalcitrant notion that persistently equates cannabis use with being harmless.

Similarly, teratogenic syndromes were chosen to see if cannabis might potentially explain some of the variance of this challenging group.

We were also interested in lateralization syndromes, including the full version of the disorder situs inversus, as there was considerable evidence from recent work that certain cardiovascular anomalies with which cannabis was implicated such as double outlet right ventricle and transposition of the great vessels included an element of malrotation or non-rotation in their dysmorphogenesis [7,14,47]. We therefore wished to see how far-ranging this left-right malrotation was in relation to cannabis teratogenesis. Left-right malrotation syndromes have also been described from cannabis epigenomically [37].

Some anomalies were chosen for several reasons as indicated.

### 4.3. Qualitative Causal Inference

In 1965, A.B. Hill outlined several criteria which would be used to assess potentially causal relationships. They include strength of association, consistency amongst studies, specificity, temporality, coherence with known data, biological plausibility, dose-response relationships, analogy with similar situations elsewhere, and experimental confirmation. It is observed that the present series of anomalies fulfills most of these criteria except those related to reproduction in other studies, which is unsurprising given this is an original report for many of these anomalies. Nor have this series of anomalies been identified in earlier studies to our knowledge. However, it is important to note that a plethora of established biological pathways exist which could well explain these findings.

### 4.4. Quantitative Causal Inference

Inverse probability weighting is the technique of choice in causal inferential studies to even out environmental exposures across groups and make them truly comparable so that causal inferences can appropriately be derived from observations datasets [109,131]. The process of inverse probability weighting is known to convert observations datasets into pseudorandomized studies so that causal inferences can properly be determined. It is noted that this was applied to all the multivariable panel models reported in the present study.

E-values quantify the degree of association required of some extraneous covariate which has not been included in the analysis with both the exposure of concern and the outcome of interest to explain away and obviate an apparently causal association. As such, it lends great confidence to an analysis as it is a form of sensitivity testing to the effects of added covariates. E-values greater than 9 are said to be high [114] and E-values in excess of 1.25 are usually taken as being indicative of potentially causal relationships [113]. Most of the E-values reported in the present study are much greater than 1.25, which greatly strengthens the conclusions drawn.

### 4.5. Mechanisms

As noted in the introduction, there are a variety of mechanisms by which cannabinoids exert genotoxic effects. It is, however, also important to consider some of the cannabinoid-related related epigenomic mechanisms which have been elucidated by recent whole genome epigenetic screens.

### 4.6. Epigenomic Controls

A recent paper cataloguing genome-wide DNA methylation changes provided great insight into the heritable mechanism underlying cannabinoid genotoxicity and teratogenesis in particular [37]. Researchers looked at 20 cannabis-dependent patients and controls both at study initiation and after 17 weeks of confirmed cannabis abstinence in the experimental group. Seventeen weeks was chosen as it is the normal sperm cycle time in human males. Hence, each patient formed their own control and investigators were able to look at the comparative epigenomes both at time zero and in cannabis withdrawal and compare them both to each other and between groups. A total of 163 differentially methylated regions (DMRs) were identified in cannabis dependence between the two groups and 127 DMRs were identified in cannabis withdrawal at the 17-week mark. These DMRs in turn affected hundreds of genes. The present consideration of the general group of anomalies provides an ideal opportunity to consider the breadth of the findings of this remarkable paper. Study results were supported by detailed online supplementary material which ran to 359 pages.

During cannabis dependence pathways annotated for cerebral disorder, neurodevelopmental disorders, agenesis (lack of growth), growth of organism, cardiogenesis, haematological and immune changes and liver lesions were defined. During cannabis withdrawal, brain changes (hippocampal formation cognitive impairments, learning, encephalopathy, quantity of pyramidal neurons), activation of alveolar macrophages, organismal death and abnormal morphology of seminiferous tubules were identified.

As to the specific genes identified by functional annotations of Ingenuity Pathway Analysis, most will be covered in a companion paper in this series dealing with specific organ systems. Consideration in this paper will be confined to those which broadly affect all body systems or are not addressed elsewhere. References are to the page in the Supplementary material of Schrott [37].

A total of 256 hits for DNA metabolism were found in cannabis dependence and withdrawal, including DNA transcription (60 genes, page 314), DNA promoter activity (49 genes, page 317), DNA replication, recombination and repair (12 genes, page 317), DNA binding (24 genes, page 323), DNA synthesis and repair (20 genes, page 323), and DNA replication, recombination and repair (4 genes, page 344).

This is a fascinating list and may explain the frequent observations of DNA and chromosomal breaks after cannabinoid exposure from failed recombination and repair events. It also explains why testicular and lymphoid cancers are more common in cannabis-exposed patients, as recombination events happen programmatically in those tissues as part of gamete crossing-over events and hypermutation in immune follicles during antigen selection processes. It is also noted that there are many annotations in this appendix for immune and haemopoietic systems which are detailed elsewhere [12,132,133].

One hit was found for mitochondria disorders which related to mitochondrial function in the eye (1 gene, RNASEH1 [Ribonuclease H1], page 357).

Very interestingly, there were two hits for disorders of microtubule dynamics (58 genes, page 300, and 24 genes, page 352). This may relate to disorders of the mitotic spindle function which is perhaps the best-recognized feature of cannabis teratology [1,2,3,4,7,10,13,14,134,135,136].

For body axis development, there was a single hit with 50 genes annotated (page 302).

There was one reference to diminished ovarian reserve (2 genes, page 349, *p* = 0.00308).

Searching limb morphogenesis, a gene called MEGF8 (multiple EGF-like domains) was encountered (page 354). In fact, there were 105 annotations in the Supplementary Appendix where this gene was identified. One of its functions is left-right patterning [137]. This may be part of the reason for the positive findings with situs inversus and lateralization syndromes in the present report and for transposition of the great vessels in others [7,14,47].

A search for embryo growth revealed 101 hits including (page 296, 83 genes) and 306 (27 genes). Such a finding may explain the reports of small babies and smaller heads on cannabis-exposed neonates [138,139]. Growth of the embryo and embryonic morphogenesis were also noted (Page 310, 39 and 15 genes each), morphogenesis of embryonic tissue (12 genes, page 300), embryonic growth and organismal development (27 genes, page 305), and differentiation of embryonic cells (15 genes, page 316).

Growth itself scored 84 hits, including cellular proliferation and outgrowth of cells (27 genes, page 295). Body trunk development was identified (50 genes, page 317). Neuronal growth was noted (25 genes, page 298), neuronal development (43 genes, page 299, Supplementary Table S4), synapse growth (15 genes, page 308), morphogenesis of breast cell lines (3 genes, page 312), formation of colony forming granulocytes (3 genes, page 312) and myogenesis of germ cell tumour and carcinoma cell lines (2 genes each, page 320).

A search found 126 hits for carcinoma, 487 hits for cancer and 112 hits for tumour, making it one of the major themes of both the Supplementary Material and the main report. Indeed, in their manuscript, Schrott and colleagues advise that they ignored all of the strong cancer signals to get at the main subject of interest. However, from other reports it may be that cannabis carcinogenicity [1,12,16,17,134,135,136,140,141,142,143,144,145,146,147,148,149,150,151,152] and its epigenomics are very highly relevant indeed to the overall subject of cannabis genotoxicity as outlined above [1,12,134,135,136,151,152,153].

Given the definition of VACTERL syndrome, it was of interest to determine if vertebrae was identified in this material. This was indeed the case and a search identified three genes at page 353 (*p* = 0.00492).

A total of 22 hits were found for bone, including abnormal morphology of bone (20 genes, page 319), (13 genes, page 334), (15 genes, page 339), bone ossification (6 genes, page 343), bone mineralization (6 genes, page 349), proliferation of bone marrow cells (5 genes, page 353), bone mineral density (6 genes, page 353), development of bone marrow cell lines, loss of bone tissue and cell movement of bone marrow cells (5, 3 and 3 genes respectively on page 323) and bone mineral trabecular layer (1 gene, IGF1, page 357, *p* = 0.00701).

This concise survey shows that the epigenomic findings of this study account for many of the clinically relevant dysmorphogenetic features encountered in cannabis teratogenesis. Further details relating to other specific systems are provided in accompanying reports [7].

Of significance, virtually all of the gene annotations identified occurred in the cannabis withdrawal samples rather than the cannabis dependence samples (the sole exception being the reference to Table S4 above). This is also clinically highly relevant as many patients will find that their cannabis supply varies across time, and indeed, drug withdrawal is one of the primary motivations to continue drug self-administration [154].

### 4.7. Morphogen Gradients

It is important to appreciate that cannabinoids broadly disrupt embryonic pattern formation by gradients of tissue morphogens which in general control the patterning, length and physical extent of most body tissues. It has been noted that cannabis disrupts many of these morphogen gradients including retinoic acid [155,156,157], Sonic Hedgehog [34], Wnt signalling [158,159,160,161,162,163], bone morphogenetic proteins [164,165,166], notch [167,168,169], neuroligin [170] and fibroblast growth factor [171,172].

Given that such gradients are foundational and fundamental to embryonic patterning and morphogenesis, it becomes easy to comprehend how their systematic disruption can lead to various and diverse teratological outcomes depending on the timing and dose of administration during the organogenic period.

### 4.8. Exponential Genotoxic Effects

One of the great concerns voiced by ourselves and others is the exponential dose-response of cannabinoids in the development of many mutagenicity and related metabolic assays [37,80,81]. Not only is this exponential dose-response well described in the laboratory, but it has now been described in a number of epidemiological reports in human populations [2,7,14,136].

Since many parts of Europe are clearly caught up in a triple confluence of increased prevalence of use, increased intensity of daily use [21,22], and increased cannabinoid concentrations, the conclusion seems inescapable that many regional populations have increased cannabinoid exposures, doubtless exacerbated by the long half-life of cannabinoids in fatty tissues in adipose, brain and gonadal stores. Further, given that in some rural areas large cannabis crops are being grown, it seems that escape of cannabinoids into the water table and probably into the food chain is occurring.

Reports from both the Ain region in northeast France and from the Brittany region indicate that increased rates of babies being born without limbs has recently occurred [173,174,175]. In both areas, cannabis crops are being cultivated. In the Ain region, calves are being born without limbs [173,174,175]. In this context concern may be expressed that it may be that cows are eating either contaminated feed or water and then their products are entering the food chain which is being manifested in elevated rates of major congenital anomalies.

Similar observations are being reported in parts of USA in relation to atrial septal defects [15].

The now epidemiologically-observed sudden and significant spike in rates of congenital anomalies is what would be predicted from laboratory studies following exponential exposure which achieved a threshold for cannabis genotoxicity.

### 4.9. Generalizability

Current study results have employed one of the most comprehensive and largest datasets globally. We also find assurance in the analyses of the anomalies in this dataset because wherever they can be paired with other published comparable results from large datasets from elsewhere, in general, similar results are obtained [1,2,14]. However, as most of the anomalies in this group have not been studied previously, it is important to see the results of similar studies undertaken on other datasets. Moreover, for the six specific anomalies which were the particular focus of this study, the results at bivariate analysis were confirmed in various multivariable models and within a pseudorandomized causal inferential analytical framework at high levels of statistical significance. For this group particularly, we would be confident that subsequent analyses would find confirmatory results.

### 4.10. Strengths and Limitations

The strengths of this study are that it was conducted on one of the largest, most comprehensive datasets in the world and employed cutting-edge analytical tools such as inverse probability weighting, E-value, and panel and geospatial regression techniques. As such, these techniques convert observational data into a pseudorandomized dataset from which it is entirely appropriate to draw causal inferences, providing a robust analytical paradigm from which to draw important conclusions. One of the great strengths of using European data is that the dataset is complete in the sense that it also includes stillbirths and early terminations for anomaly, which are notably absent from the data from many other datasets. Another strength is that these anomalies are not listed or studied by many other registries and in this sense, the dataset is more complete than many others. Ranger regression was used for formal variable selection. Like many epidemiological studies, the present work did not have available individual participant cannabis exposure and anomaly outcome data. Nor was it possible to complete full analyses on all the anomalies listed. Like most epidemiological studies, this study is not mechanistic in the experimental sense. However, observations made here, together with the complex mechanistic underpinning, strongly indicate additional laboratory work to be performed to further investigate the observed relationships in a formal experimental setting.

## 5. Conclusions

Nine of the eleven anomalies studied in this section demonstrate strong and robust bivariate associations with metrics of cannabis exposure, the two exceptions being conjoined twins and valproate syndrome. All six of the congenital anomalies studied in multivariable detail, namely all anomalies, VACTERL syndrome, FAS, situs inversus, lateralization syndromes and teratogenic syndromes demonstrated strong and robust associations with cannabis exposure metrics which survived multivariable adjustment and persisted in final inverse probability weighted panel and spatiotemporal models. In each case, high E-values and inverse probability weighting fulfilled quantitative epidemiological criteria for causal relationships. These results confirm earlier preliminary results on this dataset and are consistent with other recent reports which have found that rates of diverse groups of congenital anomalies are increased in association with cannabinoid exposure. The very robust response of the VACTERL dataset to the effects of cannabinoids is consistent with the prediction that the syndrome is caused by cannabinoid-induced inhibition of Sonic Hedgehog. The data for teratogenic syndromes is similarly consistent with a significant contribution to the aetiopathology of this group of disorders coming from cannabinoids. The data for situs inversus and lateralization syndromes similarly strongly implicate cannabinoids as an important contributing cause. This is intriguing as transposition of the great arteries, which may be considered to be a *forme fruste* of these disorders, has been identified in several other studies as being an anomaly which is cannabis-related [14,50]. The present results support this conclusion. Epigenomic mechanisms may well explain many of the dysmorphogenetic features of cannabis teratogenesis. Particular concern is expressed at the rapidly-rising confluence of cannabinoid exposure in many parts of Europe with the known exponential genotoxic dose-response curve, and that the recent serious French experience with limb reduction anomalies may be portentous of more such outbreaks to come. Given the present powerful findings it would appear important to limit community exposure to powerful teratogens in the interests of protection of the genome and epigenome for coming generations.

## Figures and Tables

**Table 1:** Absolute and Relative Rates of Anomalies in Nations with Increasing Daily Cannabis Use to Those without, Together with Significance Levels.

Anomaly	Mean ± S.E. Increasing	Mean ± S.E. Decreasing	Relative Rate Incr./Decr.	Student’s *t*	*p*-Value
VATER/VACTERL	0.46 (0.42, 0.5)	0.02 (0.03, 0.07)	31.820	8.3870	7.49× 10^−13^
Teratogenic Synds	1.07 (0.52, 1.62)	0.28 (−0.09, 0.23)	3.830	6.1691	1.81 × 10^−8^
Matern Infect Malform	0.73 (0.44, 1.02)	0.15 (−0.08, 0.2)	4.748	5.7942	1.01 × 10^−7^
Situs inversus	0.63 (−0.06, 1.32)	0.35 (−0.05, 0.15)	1.789	3.6097	5.18 × 10^−4^
Fetal Alcohol	0.25 (0.01, 0.49)	0.12 (−0.04, 0.12)	2.161	2.3870	0.0186
Lateral anomalies	1.52 (−0.48, 3.52)	1.02 (−0.33, 0.45)	1.497	1.5385	0.1374
Valproate syndrome	0.05 (0.01, 0.09)	0.02 (0, 0.04)	2.104	1.2358	0.2190
Conjoined twins	0.12 (−0.04, 0.28)	0.08 (−0.02, 0.06)	1.448	1.1442	0.2555
Skeletal dysplasias	1.55 (−0.9, 4)	1.25 (−0.21, 0.33)	1.239	1.1265	0.2656
Amniotic band	0.34 (−0.19, 0.87)	0.27 (−0.14, 0.22)	1.261	0.6950	0.4902
All Anomalies	233.39 (−251.87, 718.65)	247.58 (−0.14, 0.22)	0.943	0.6372	0.5271

**Table 2:** Positive and significant slopes for bivariate analysis of CA rates by substance exposure.

Anomaly	Substance	Mean Anomaly Rate	Estimate	Std. Error	Sigma	t_Statistic	*p*_Value	E-Value Estimate	E-Value Lower Bound
VATER/VACTERL	Daily.Interpol.	0.4377	20.8858	4.6484	0.4252	4.4932	4.4932	1.81 × 10^−5^	5.19 × 10^19^
Teratogenic Synds	Daily.Interpol.	1.0683	29.9733	6.8274	0.6408	4.3901	4.3901	2.56 × 10^−5^	6.10 × 10^18^
Lateral anomalies	Daily.Interpol.	1.7332	26.6028	6.7914	0.6212	3.9172	3.9172	1.60 × 10^−4^	1.68 × 10^17^
Matern Infect Malform	Daily.Interpol.	0.6880	22.6665	5.8194	0.5462	3.8950	3.8950	1.67 × 10^−4^	5.02 × 10^16^
Teratogenic Synds	LMCannabis_Herb	1.0683	11.1368	2.1817	0.6263	5.1046	5.1046	1.26 × 10^−6^	2.13 × 10^7^
Situs inversus	Daily.Interpol.	0.5932	12.3967	4.2919	0.4029	2.8884	2.8884	0.0046	2.90 × 10^12^
Lateral anomalies	LMCannabis_Herb	1.7332	10.0481	2.3741	0.6147	4.2324	4.2324	4.96 × 10^−5^	5.78 × 10^6^
Matern Infect Malform	LMCannabis_Herb	0.6880	8.2647	1.8585	0.5335	4.4469	4.4469	1.96 × 10^−5^	2.65 × 10^6^
Skeletal dysplasias	Daily.Interpol.	1.8050	19.3566	7.0728	0.6639	2.7368	2.7368	0.0072	6.67 × 10^11^
Lateral anomalies	Herb	1.7332	8.3890	1.8336	0.6072	4.5752	4.5752	1.31 × 10^−5^	5.76 × 10^5^
Teratogenic Synds	Herb	1.0683	7.7193	1.6312	0.6343	4.7322	4.7322	6.13 × 10^−6^	1.29 × 10^5^
Matern Infect Malform	Herb	0.6880	6.2822	1.3652	0.5308	4.6016	4.6016	1.05 × 10^−5^	9.51 × 10^4^
Situs inversus	LMCannabis_Herb	0.5932	5.1389	1.3379	0.3840	3.8410	3.8410	1.97 × 10^−4^	3.88 × 10^5^
Situs inversus	Herb	0.5932	4.0862	0.9779	0.3802	4.1784	4.1784	5.60 × 10^−5^	3.53 × 10^4^
Fetal Alcohol	LMCannabis_Herb	0.2458	4.3930	1.2221	0.3508	3.5945	3.5945	4.73 × 10^−4^	1.78 × 10^5^
VATER/VACTERL	LM_Cannabis	0.4377	6.5421	2.0669	0.4436	3.1652	3.1652	0.0020	1.35 × 10^6^
Teratogenic Synds	LM_Cannabis	1.0683	8.8997	2.7367	0.6624	3.2519	3.2519	0.0015	4.09 × 10^5^
Lateral anomalies	LM_Cannabis	1.7332	8.9858	2.9721	0.6378	3.0234	3.0234	0.0031	7.39 × 10^5^
VATER/VACTERL	LMCannabis_Herb	0.4377	5.1942	1.7200	0.4453	3.0199	3.0199	0.0032	8.14 × 10^4^
Matern Infect Malform	LM_Cannabis	0.6880	6.4725	2.3045	0.5577	2.8087	2.8087	0.0058	7.72 × 10^4^
Skeletal dysplasias	LMCannabis_Herb	1.8050	6.5116	2.2481	0.6453	2.8965	2.8965	0.0045	1.94 × 10^4^
Teratogenic Synds	LM.Cannabis × Herb.THC × Daily.Interpol.	1.0683	3.1437	0.7564	0.6457	4.1562	4.1562	6.32 × 10^−5^	167.39
Lateral anomalies	Resin	1.7332	3.0137	0.7391	0.6363	4.0773	4.0773	9.46 × 10^−5^	148.33
Fetal Alcohol	Herb	0.2458	2.6533	0.9181	0.3570	2.8898	2.8898	0.0046	1.73 × 10^3^
Matern Infect Malform	LM.Cannabis × Herb.THC × Daily.Interpol.	0.6880	2.3897	0.6433	0.5492	3.7149	3.7149	3.18 × 10^−4^	104.39
Lateral anomalies	LM.Cannabis × Herb.THC × Daily.Interpol.	1.7332	2.4982	0.7511	0.6325	3.3259	3.3259	0.0012	72.26
All Anomalies	Resin	255.4744	1.2426	0.3661	0.3291	3.3942	3.3942	9.72 × 10^−4^	61.62
Fetal Alcohol	Daily.Interpol.	0.2458	8.2930	3.9391	0.3697	2.1053	2.1053	0.0375	1.46 × 10^9^
Lateral anomalies	LMCannabis_Resin	1.7332	2.1164	0.5631	0.6436	3.7586	3.7586	2.95 × 10^−4^	39.37
Skeletal dysplasias	Herb	1.8050	4.2006	1.6733	0.6506	2.5103	2.5103	0.0134	711.51
Amniotic band	LMCannabis_Resin	0.3730	1.0556	0.3089	0.3699	3.4170	3.4170	9.02 × 10^−4^	26.33
Skeletal dysplasias	LM.Cannabis × Herb.THC × Daily.Interpol.	1.8050	2.2295	0.7751	0.6617	2.8764	2.8764	0.0048	42.40
Situs inversus	LM.Cannabis × Herb.THC × Daily.Interpol.	0.5932	1.3525	0.4721	0.4031	2.8647	2.8647	0.0050	41.87
Teratogenic Synds	LMCannabis_Resin	1.0683	1.8066	0.5687	0.6810	3.1768	3.1768	0.0020	21.85
Fetal Alcohol	LM.Cannabis × Herb.THC × Daily.Interpol.	0.2458	1.1590	0.4278	0.3653	2.7090	2.7090	0.0078	35.39
Teratogenic Synds	LM.Cannabis × Resin.THC × Daily.Interpol.	1.0683	1.2927	0.3585	0.6708	3.6062	3.6062	4.91 × 10^−4^	11.03
Fetal Alcohol	LMCannabis_Resin	0.2458	0.8989	0.3077	0.3684	2.9213	2.9213	0.0043	17.90
Matern Infect Malform	LMCannabis_Resin	0.6880	1.3658	0.4763	0.5703	2.8674	2.8674	0.0050	17.16
All Anomalies	Herb	255.4744	2.2466	1.0012	0.3893	2.2438	2.2438	0.0267	381.13
Matern Infect Malform	LM.Cannabis × Resin.THC × Daily.Interpol.	0.6880	0.9847	0.3031	0.5672	3.2483	3.2483	0.0016	9.18
VATER/VACTERL	LM.Cannabis × Herb.THC × Daily.Interpol.	0.4377	1.3541	0.5352	0.4507	2.5300	2.5300	0.0129	30.28
Lateral anomalies	LM.Cannabis × Resin.THC × Daily.Interpol.	1.7332	1.1244	0.3521	0.6555	3.1934	3.1934	0.0019	9.00
Amniotic band	LMCannabis_Herb	0.3730	3.2048	1.4989	0.4302	2.1381	2.1381	0.0345	1.76 × 10^3^
VATER/VACTERL	Cocaine	0.4377	0.3393	0.0527	0.3931	6.4369	6.4369	3.72 × 10^−9^	3.81
Skeletal dysplasias	LMCannabis_Resin	1.8050	1.3870	0.5266	0.6306	2.6337	2.6337	0.0097	14.28
Skeletal dysplasias	LM.Cannabis × Resin.THC × Daily.Interpol.	1.8050	1.0055	0.3440	0.6436	2.9233	2.9233	0.0043	7.75
All Anomalies	LMCannabis_Resin	255.4744	0.7027	0.2813	0.3368	2.4982	2.4982	0.0140	12.83
All Anomalies	LM.Cannabis × Resin.THC × Daily.Interpol.	255.4744	0.4992	0.1811	0.3388	2.7572	2.7572	0.0070	7.11
Matern Infect Malform	Cocaine	0.6880	0.3460	0.0644	0.5169	5.3762	5.3762	3.80 × 10^−7^	3.08
Fetal Alcohol	LM.Cannabis × Resin.THC × Daily.Interpol.	0.2458	0.5533	0.2024	0.3787	2.7344	2.7344	0.0074	7.02
Teratogenic Synds	Cocaine	1.0683	0.3971	0.0780	0.6266	5.0903	5.0903	1.34 × 10^−6^	2.96
Amniotic band	LM.Cannabis × Resin.THC × Daily.Interpol.	0.3730	0.5439	0.2053	0.3842	2.6491	2.6491	0.0094	6.71
VATER/VACTERL	LM.Cannabis × Herb.THC: LM.Cannabis × Resin.THC × Daily.Interpol.	0.4377	0.9512	0.4034	0.4610	2.3582	2.3582	0.0204	12.55
Lateral anomalies	Cocaine	1.7332	0.3713	0.0815	0.6077	4.5565	4.5565	1.41 × 10^−5^	2.88
VATER/VACTERL	LM.Cannabis × Resin.THC × Daily.Interpol.	0.4377	0.6332	0.2464	0.4587	2.5702	2.5702	0.0117	6.48
Situs inversus	LM.Cannabis × Resin.THC × Daily.Interpol.	0.5932	0.5669	0.2250	0.4211	2.5194	2.5194	0.0134	6.27
Situs inversus	Cocaine	0.5932	0.2054	0.0471	0.3781	4.3619	4.3619	2.74 × 10^−5^	2.66
Situs inversus	LMCannabis_Resin	0.5932	0.7543	0.3444	0.4123	2.1905	2.1905	0.0307	10.04
Skeletal dysplasias	Cocaine	1.8050	0.2686	0.0794	0.6378	3.3820	3.3820	9.72 × 10^−4^	2.29
All Anomalies	Cocaine	255.4744	0.1491	0.0476	0.3821	3.1349	3.1349	0.0022	2.21
Fetal Alcohol	Annual_Alcohol	0.2458	0.0793	0.0169	0.3393	4.6992	4.6992	7.02 × 10^−6^	1.78
All Anomalies	Daily.Interpol.	255.4744	0.1426	0.0481	0.3836	2.9645	2.9645	0.0037	2.15
VATER/VACTERL	Amphetamine	0.4377	0.1506	0.0582	0.4501	2.5891	2.5891	0.0110	2.05
Amniotic band	Annual_Alcohol	0.3730	0.0709	0.0208	0.4186	3.4067	3.4067	8.95 × 10^−4^	1.61
Teratogenic Synds	Annual_Alcohol	1.0683	0.0942	0.0333	0.6690	2.8296	2.8296	0.0055	1.53
Valproate syndrome	Cocaine	0.0434	0.0362	0.0169	0.1354	2.1472	2.1472	0.0338	1.87

Table key: LM.Cannabis—last month cannabis use; Herb.THC—THC concentration of cannabis herb; Resin.THC—THC concentration of cannabis herb; Daily.Interpol.—daily cannabis use interpolated.

**Table 3:** Multivariable geospatial analysis of All Congenital Anomalies.

Parameter Values			Model Parameters		
Parameter	Estimate (C.I.)	*p*-Value	Parameter	Value	Significance
					
***Additive***					
*Rate ~ Tobacco + Alcohol + LM.Cannabis × Herb.THC × Daily.Interpol. + LM.Cannabis × Resin.THC × Daily.Interpol. + Daily.Interpol. + LM.Cannabis × Resin.THC + Amphetamines + Cocaine + Income)*					
LM.Cannabis × Resin.THC	0.94 (0.53, 1.36)	8.96 × 10^−6^	psi	0.9116	<2.2 × 10^−16^
Cocaine	0.09 (0.02, 0.17)	0.0136	rho	0.6488	2.81 × 10^−12^
			lambda	−0.4147	0.00167
					
***Interactive***					
Rate ~ Tobacco + LM.Cannabis × Resin.THC * Herb + LM.Cannabis × Resin.THC × Daily.Interpol. + LM.Cannabis × Herb.THC × Daily.Interpol. + Alcohol + Amphetamines + Cocaine + Income					
Herb	2.02 (1.13, 2.9)	8.09 × 10^−6^	psi	0.9073	<2.2 × 10^−16^
LM.Cannabis × Herb.THC × Daily.Interpol.	0.05 (0.01, 0.1)	2.23 × 10^−2^	rho	−0.5330	1.50 × 10^−5^
			lambda	0.5605	2.34 × 10^−7^
					
***2 Lags***					
Rate ~ Tobacco + LM.Cannabis × Resin.THC * Herb + LM.Cannabis × Resin.THC × Daily.Interpol. + LM.Cannabis × Herb.THC × Daily.Interpol. + Alcohol + Amphetamines + Cocaine + Income					
LM.Cannabis × Resin.THC	0.74 (0.24, 1.23)	0.0037	psi	0.9215	<2.2 × 10^−16^
LM.Cannabis × Resin.THC × Daily.Interpol.	0.06 (0.01, 0.1)	0.0198	rho	0.6514	1.89 × 10^−8^
Cocaine	−0.09 (−0.17, −0.01)	0.0304	lambda	−0.406	0.013

Table key: Abbreviations as in Table 2.

**Table 4:** Multivariable geospatial analysis of VACTERL Syndrome Rates.

Parameter Values			Model Parameters		
Parameter	Estimate (C.I.)	*p*-Value	Parameter	Value	Significance
					
***Additive***					
***Rate ~ Tobacco + Alcohol + LM.Cannabis × Herb.THC × Daily.Interpol. + LM.Cannabis × Resin.THC × Daily.Interpol. + Daily.Interpol. + LM.Cannabis × Resin.THC + Amphetamines + Cocaine + Income)***					
Alcohol	0.07 (0, 0.13)	0.0472	psi	0.5261	5.52 × 10^−12^
Daily.Interpol.	15.4 (2.37, 28.43)	0.0205	Log.Lik.	−26.1038	
Income	0 (0, 0)	0.0011			
					
***Interactive***					
Rate ~ Tobacco * Daily.Interpol. + LM.Cannabis × Herb.THC + LM.Cannabis × Resin.THC × Daily.Interpol. + LM.Cannabis × Herb.THC × Daily.Interpol. + Alcohol + Amphetamines + Cocaine + Income					
Tobacco	0.04 (0, 0.07)	0.0383	psi	0.2864	0.00512
Daily.Interpol.	118 (50.77, 185.23)	0.0006			
Alcohol	0.1 (0.05, 0.16)	0.0002			
Cocaine	0.29 (0.06, 0.51)	0.0121			
Income	0 (0, 0)	0.0097			
Tobacco: Daily.Interpol.	−4.97 (−7.56, −2.38)	0.0002			
					
***2 Lags***					
Rate ~ Tobacco * Daily.Interpol. + LM.Cannabis × Herb.THC + LM.Cannabis × Resin.THC × Daily.Interpol. + LM.Cannabis × Herb.THC × Daily.Interpol. + Alcohol + Amphetamines + Cocaine + Income					
Tobacco	0.04 (0, 0.07)	0.029285	Least Squares		
Daily.Interpol.	184 (103.84, 264.16)	6.56 × 10^−6^	S.D.	0.3114	
Alcohol	0.11 (0.06, 0.16)	1.90 × 10^−5^	Log.Lik.	−22.2103	
Cocaine	0.33 (0.09, 0.57)	0.0072			
Income	0 (0, 0)	0.0291			
Tobacco: Daily.Interpol.	−7.43 (−10.33, −4.53)	4.97 × 10^−7^			
					
***4 Lags***					
Rate ~ Tobacco * Daily.Interpol. + LM.Cannabis × Herb.THC + LM.Cannabis × Resin.THC × Daily.Interpol. + LM.Cannabis × Herb.THC × Daily.Interpol. + Alcohol + Amphetamines + Cocaine + Income					
Tobacco	0.06 (0.03, 0.09)	0.0002	Least Squares		
Daily.Interpol.	277 (197.82, 356.18)	6.81 × 10^−12^	S.D.	0.2859	
LM.Cannabis × Herb.THC	7.9 (2.84, 12.96)	0.0022			
Alcohol	0.09 (0.04, 0.13)	0.0001			
Income	0 (0, 0)	3.26 × 10^−5^			
Tobacco: Daily.Interpol.	−10.6 (−13.68, −7.52)	1.29 × 10^−11^			
					
***6 Lags***					
Rate ~ Tobacco * Daily.Interpol. + LM.Cannabis × Herb.THC + LM.Cannabis × Resin.THC × Daily.Interpol. + LM.Cannabis × Herb.THC × Daily.Interpol. + Alcohol + Amphetamines + Cocaine + Income					
Tobacco	0.12 (0.07, 0.16)	9.80 × 10^−7^	Least Squares		
Daily.Interpol.	375 (255.44, 494.56)	8.10 × 10^−10^	S.D.	0.2953	
LM.Cannabis × Herb.THC: LM.Cannabis × Resin.THC × Daily.Interpol.	−0.31 (−0.46, −0.17)	3.13 × 10^−5^			
Income	0 (0, 0)	4.57 × 10^−6^			
Tobacco: Daily.Interpol.	−13.8 (−18.43, −9.17)	4.95 × 10^−9^			

Table key: Abbreviations as in Table 2.

**Table 5:** Comparing geospatial VACTERL Models with and without cannabis.

Parameter Values			Model Parameters		
Parameter	Estimate (C.I.)	*p*-Value	Parameter	Value	Significance
***Additve Model without Cannabis Terms***					
*Rate ~ Alcohol + Cocaine + Income*					
Alcohol	0.09 (0.04, 0.15)	0.0010	psi	3.73 × 10^−7^	
Resin	0.24 (0.1, 0.38)	0.0007	S.D.	0.3495	
Income	0 (0, 0)	0.0059	Log.Lik.	−28.449	
					
			***Spatial Hausman Test***		
			Chi.Squared	8.12	
			Deg.Freedom	3	
			*p*-Value		0.0436
					
***Models at 2 Lags without Cannabis Terms***					
Alcohol	0.07 (0.02, 0.11)	0.0029	Least Squares		
Cocaine	0.4 (0.28, 0.52)	2.90 × 10^−11^	S.D.	0.3796	
			Log.Lik.	−39.6349	
					
			***Spatial Hausman Test***		
			Chi.Squared	82.41	
			Deg.Freedom	3	
			*p*-Value		<2.2 × 10^−16^

**Table 6:** Multivariable geospatial analysis of Fetal Alcohol Syndrome Rates.

Parameter Values			Model Parameters		
Parameter	Estimate (C.I.)	*p*-Value	Parameter	Value	Significance
					
***Additive***					
*Rate ~ Tobacco + Alcohol + LM.Cannabis × Herb.THC × Daily.Interpol. + LM.Cannabis × Resin.THC × Daily.Interpol. + Daily.Interpol. + LM.Cannabis × Resin.THC + Amphetamines + Cocaine + Income)*					
Tobacco	0.05 (0.03, 0.06)	3.96 × 10^−9^	rho	−0.4998	7.08 × 10^−5^
Alcohol	0.06 (0.03, 0.08)	1.32 × 10^−5^	lambda	0.4343	9.82 × 10^−5^
Herb	2.46 (1.1, 3.82)	0.0004			
Amphetamines	−0.11 (−0.17, −0.05)	0.0004			
Income	0 (0, 0)	5.36 × 10^−8^			
					
***Interactive***					
Rate ~ Tobacco + LM.Cannabis × Resin.THC * Herb + LM.Cannabis × Herb.THC × Daily.Interpol. + LM.Cannabis × Resin.THC × Daily.Interpol. + Alcohol + Amphetamines + Cocaine + Income					
Tobacco	0.05 (0.03, 0.06)	3.96 × 10^−9^	rho	−0.4998	7.10 × 10^−5^
Herb	2.46 (1.1, 3.82)	0.0004	lambda	0.4343	9.79 × 10^−5^
Alcohol	0.06 (0.03, 0.08)	1.32 × 10^−5^			
Amphetamines	−0.11 (−0.17, −0.05)	0.0004			
Income	0 (0, 0)	5.36 × 10^−8^			
					
***2 Lags***					
Rate ~ Tobacco + LM.Cannabis × Resin.THC * Herb + LM.Cannabis × Resin.THC × Daily.Interpol. + LM.Cannabis × Herb.THC × Daily.Interpol. + Alcohol + Amphetamines + Cocaine + Income					
Tobacco	0.05 (0.04, 0.07)	1.36 × 10^−10^	rho	−0.6212	3.41 × 10^−9^
LM.Cannabis × Resin.THC × Daily.Interpol.	1.41 (0.47, 2.35)	0.0034	lambda	0.4866	1.44 × 10^−7^
LM.Cannabis × Herb.THC × Daily.Interpol.	−2.92 (−5.06, −0.78)	0.0076			
Alcohol	0.07 (0.04, 0.1)	9.45 × 10^−6^			
Amphetamines	−0.18 (−0.26, −0.1)	1.09 × 10^−5^			
Income	0 (0, 0)	6.49 × 10^−12^			

Table key: Abbreviations as in Table 2.

**Table 7:** Multivariable geospatial analysis of Situs Inversus Rates.

Parameter Values			Model Parameters		
Parameter	Estimate (C.I.)	*p*-Value	Parameter	Value	Significance
					
***Additive***					
*Rate ~ Tobacco + Alcohol + LM.Cannabis × Herb.THC × Daily.Interpol. + LM.Cannabis × Resin.THC × Daily.Interpol. + Daily.Interpol. + LM.Cannabis × Resin.THC + Amphetamines + Cocaine + Income)*					
Alcohol	0.04 (0.01, 0.07)	0.0169	Least Squares		
Herb	2.99 (1.11, 4.87)	0.0019	S.D.	0.2519	
Amphetamines	−0.2 (−0.27, −0.12)	1.74 × 10^−7^			
Cocaine	0.2 (0.11, 0.28)	9.22 × 10^−6^			
Income	0 (0, 0)	4.11 × 10^−6^			
					
***Interactive***					
Rate ~ Tobacco + LM.Cannabis × Resin.THC * Herb + LM.Cannabis × Resin.THC × Daily.Interpol. + LM.Cannabis × Herb.THC × Daily.Interpol. + Alcohol + Amphetamines + Cocaine + Income					
Herb	2.99 (1.11, 4.87)	0.0019	Least Squares		
Alcohol	0.04 (0.01, 0.07)	0.0169	S.D.	0.2519	
Amphetamines	−0.2 (−0.27, −0.12)	1.74 × 10^−7^			
Cocaine	0.2 (0.11, 0.28)	9.22 × 10^−6^			
Income	0 (0, 0)	4.11 × 10^−6^			
					
***1 Lags***					
Rate ~ Tobacco + LM.Cannabis × Resin.THC * Herb + LM.Cannabis × Resin.THC × Daily.Interpol. + LM.Cannabis × Herb.THC × Daily.Interpol. + Alcohol + Amphetamines + Cocaine + Income					
Herb	2.37 (0.23, 4.51)	0.0299	Least Squares		
Amphetamines	−0.19 (−0.27, −0.1)	1.64 × 10^−5^	S.D.	0.3024	
Cocaine	0.18 (0.08, 0.28)	0.0003			
Income	0 (0, 0)	5.31 × 10^−5^			

Table key: Abbreviations as in Table 2.

**Table 8:** Multivariable geospatial analysis of Lateralization Rates.

Parameter Values			Model Parameters		
Parameter	Estimate (C.I.)	*p*-Value	Parameter	Value	Significance
					
***Additive***					
*Rate ~ Tobacco + Alcohol + LM.Cannabis × Herb.THC × Daily.Interpol. + LM.Cannabis × Resin.THC × Daily.Interpol. + Daily.Interpol. + LM.Cannabis × Resin.THC + Amphetamines + Cocaine + Income)*					
Alcohol	0.12 (0.06, 0.18)	2.50 × 10^−5^	rho	0.4947	4.86 × 10^−5^
LM.Cannabis × Resin.THC	3.31 (1.42, 5.2)	0.0006	lambda	−0.5897	1.68 × 10^−8^
LM.Cannabis × Resin.THC × Daily.Interpol.	−1.83 (−3.58, −0.08)	0.0412			
LM.Cannabis × Herb.THC × Daily.Interpol.	2.09 (0.01, 4.17)	0.0476			
Amphetamines	−0.15 (−0.28, −0.03)	0.0182			
Cocaine	0.29 (0.14, 0.44)	0.0001			
Income	0 (0, 0)	3.88 × 10^−7^			
					
***Interactive***					
Rate ~ Tobacco + LM.Cannabis × Resin.THC * Herb + LM.Cannabis × Resin.THC × Daily.Interpol. + LM.Cannabis × Herb.THC × Daily.Interpol. + Alcohol + Amphetamines + Cocaine + Income					
LM.Cannabis × Resin.THC	3.31 (1.42, 5.2)	0.0006	rho	0.4947	4.89 × 10^−5^
LM.Cannabis × Resin.THC × Daily.Interpol.	−1.83 (−3.58, −0.08)	0.0412	lambda	−0.5897	1.69 × 10^−8^
LM.Cannabis × Herb.THC × Daily.Interpol.	2.09 (0.01, 4.17)	0.0476			
Alcohol	0.12 (0.06, 0.18)	2.50 × 10^−5^			
Amphetamines	−0.15 (−0.28, −0.03)	0.0182			
Cocaine	0.29 (0.14, 0.44)	0.0001			
Income	0 (0, 0)	3.88 × 10^−7^			
					
***2 Lags***					
Rate ~ Tobacco + LM.Cannabis × Resin.THC * Herb + LM.Cannabis × Resin.THC × Daily.Interpol. + LM.Cannabis × Herb.THC × Daily.Interpol. + Alcohol + Amphetamines + Cocaine + Income					
LM.Cannabis × Resin.THC	1.65 (0.34, 2.96)	0.0134	rho	0.4855	0.00038
LM.Cannabis × Resin.THC × Daily.Interpol.	−0.05 (−0.09, −0.01)	0.0083	lambda	−0.6324	1.91 × 10^−8^
Alcohol	0.14 (0.08, 0.21)	1.01 × 10^−5^			
Amphetamines	−0.18 (−0.33, −0.04)	0.014951			
Cocaine	0.32 (0.16, 0.49)	0.0002			
Income	0 (0, 0)	1.66 × 10^−5^			

Table key: Abbreviations as in Table 2.

**Table 9:** Multivariable geospatial analysis of Teratogenic Syndrome Rates.

Parameter Values			Model Parameters		
Parameter	Estimate (C.I.)	*p*-Value	Parameter	Value	Significance
					
***Additive***					
*Rate ~ Tobacco + Alcohol + LM.Cannabis × Herb.THC × Daily.Interpol. + LM.Cannabis × Resin.THC × Daily.Interpol. + Daily.Interpol. + LM.Cannabis × Resin.THC + Amphetamines + Cocaine + Income)*					
Tobacco	0.06 (0.03, 0.09)	7.85 × 10^−6^	rho	−0.2466	0.1750
Alcohol	0.16 (0.1, 0.21)	7.80 × 10^−9^	lambda	0.2035	0.2040
LM.Cannabis × Resin.THC	−2.08 (−3.75, −0.41)	0.0146	S.D.	0.4193	
Herb	4.39 (1.45, 7.33)	0.0035	Log.Lik	−63.7327	
LM.Cannabis × Resin.THC × Daily.Interpol.	3.86 (1.74, 5.98)	0.0003			
LM.Cannabis × Herb.THC × Daily.Interpol.	−7.12 (−11.06, −3.18)	0.0004			
Amphetamines	−0.31 (−0.44, −0.18)	1.55 × 10^−6^			
Cocaine	0.41 (0.21, 0.61)	7.76 × 10^−5^			
Income	0 (0, 0)	7.22 × 10^−8^			
					
***Interactive***					
Rate ~ Tobacco * Herb + LM.Cannabis × Resin.THC × Daily.Interpol. + LM.Cannabis × Resin.THC + LM.Cannabis × Herb.THC × Daily.Interpol. + Alcohol + Amphetamines + Cocaine + Income					
Tobacco	0.14 (0.08, 0.19)	1.90 × 10^−7^	rho	−0.2964	0.0830
Herb	25.8 (12.3, 39.3)	0.0002	lambda	0.2477	0.0959
LM.Cannabis × Resin.THC × Daily.Interpol.	4.13 (2.11, 6.15)	5.65 × 10^−5^			
LM.Cannabis × Resin.THC	−1.58 (−3.19, 0.03)	0.053753			
LM.Cannabis × Herb.THC × Daily.Interpol.	−8.65 (−12.49, −4.81)	9.86 × 10^−6^			
Alcohol	0.2 (0.14, 0.26)	1.60 × 10^−11^			
Amphetamines	−0.36 (−0.49, −0.24)	1.32 × 10^−8^			
Cocaine	0.54 (0.33, 0.74)	4.82 × 10^−7^			
Income	0 (0, 0)	4.93 × 10^−5^			
Tobacco: Herb	−0.88 (−1.42, −0.34)	0.0014			
					
***2 Lags***					
Rate ~ Tobacco * LM.Cannabis × Resin.THC × Daily.Interpol. + Herb + LM.Cannabis × Resin.THC + LM.Cannabis × Herb.THC × Daily.Interpol. + Alcohol + Amphetamines + Cocaine + Income					
Tobacco	0.04 (0.01, 0.07)	9.22 × 10^−3^	rho	−0.4671	0.0021
LM.Cannabis × Resin.THC × Daily.Interpol.	5 (1.71, 8.29)	0.0029	lambda	0.3779	0.0091
Herb	4.95 (1.01, 8.89)	0.0137			
LM.Cannabis × Resin.THC	−4.14 (−7.28, −1)	0.0098			
LM.Cannabis × Herb.THC × Daily.Interpol.	−5.9 (−9.8, −2)	0.0030			
Alcohol	0.14 (0.09, 0.19)	2.04 × 10^−8^			
Amphetamines	−0.25 (−0.36, −0.13)	3.03 × 10^−5^			
Cocaine	0.33 (0.13, 0.54)	0.0014			
Income	0 (0, 0)	3.79 × 10^−3^			

Table key: Abbreviations as in Table 2.

**Table 10:** Comparing geospatial models of Teratogenic Syndromes with and without cannabis terms.

Parameter Values			Model Parameters		
Parameter	Estimate (C.I.)	*p*-Value	Parameter	Value	Significance
***Additive Model without Cannabis Terms***					
*Rate ~ Tobacco + Alcohol + Amphetamines + Cocaine + Income*					
Income	0 (0, 0)	2.57 × 10^−12^	Least Squares		
			S.D.	0.6399	
			Log.Lik.	−106.99	
					
					
			***Spatial Hausman Test***		
			Chi.Squared	184.20	
			Deg.Freedom	2	
			*p*-Value		<2.2 × 10^−16^

**Table 11:** E-values from panel models.

Anomaly	Term	*p*-Value	E-Value Estimate	Lower Bound E-Value
				
All Anomalies				
	***Additive***			
	LM.Cannabis × Resin.THC × Daily.Interpol.	0.0001	1.75	1.45
	***Interactive***			
	LM.Cannabis × Herb.THC × Daily.Interpol.	<2.2 × 10^−16^	212.22	106.93
	LM.Cannabis × Resin.THC × Daily.Interpol.: LM.Cannabis × Herb.THC × Daily.Interpol.	7.81 × 10^−15^	5.67	4.29
	***1 Lag***			
	Tobacco: LM.Cannabis × Resin.THC × Daily.Interpol.	<2.2 × 10^−16^	1.42	1.38
	LM.Cannabis × Resin.THC × Daily.Interpol.: LM.Cannabis × Herb.THC × Daily.Interpol.	<2.2 × 10^−16^	1.69	1.62
	***2 Lags***			
	LM.Cannabis × Herb.THC × Daily.Interpol.	0.0176	4.47 × 10^15^	1.76 × 10^3^
	Tobacco: LM.Cannabis × Resin.THC × Daily.Interpol.	2.66 × 10^−8^	1.77	1.57
	LM.Cannabis × Resin.THC × Daily.Interpol.: LM.Cannabis × Herb.THC × Daily.Interpol.	0.0303	73.47	2.36
				
VACTERL				
	***Additive***			
	LM.Cannabis × Resin.THC × Daily.Interpol.	1.52 × 10^−12^	230.82	72.72
	Herb	0.0042	1.42 × 10^4^	37.93
	***Interactive***			
	Tobacco: LM.Cannabis × Resin.THC × Daily.Interpol.	4.72 × 10^−5^	1.26	1.18
	***1 Lag***			
	Daily.Interpol.	0.0185	3.32 × 10^59^	1.67 × 10^11^
	***4 Lags***			
	LM.Cannabis × Herb.THC × Daily.Interpol.	0.0009	5.93 × 10^38^	1.56 × 10^17^
				
FAS				
	***Additive***			
	LM.Cannabis × Resin.THC × Daily.Interpol.	1.44 × 10^−13^	17.04	10.05
	***Interactive***			
	LM.Cannabis × Resin.THC × Daily.Interpol.	0.0020	1.57 × 10^4^	61.02
	LM.Cannabis × Resin.THC × Daily.Interpol.: LM.Cannabis × Herb.THC × Daily.Interpol.	0.0016	4.78	2.31
	***2 Lags***			
	LM.Cannabis × Resin.THC × Daily.Interpol.	6.97 × 10^−7^	1.00 × 10^18^	3.56 × 10^11^
	Herb	0.0070	4.29 × 10^4^	36.32
	Tobacco: LM.Cannabis × Herb.THC × Daily.Interpol.	1.31 × 10^−5^	6.65	3.61
				
Situs Inversus				
	***Additive***			
	LM.Cannabis × Herb.THC × Daily.Interpol.	0.0034	8.35	2.86
	LM.Cannabis × Herb.THC	5.44 × 10^−6^	7.97 × 10^10^	6.73 × 10^5^
	***Interactive***			
	LM.Cannabis × Herb.THC × Daily.Interpol.	0.0061	50.05	4.74
	Tobacco: Resin	1.26 × 10^−5^	3.01	2.15
	***2 Lags***			
	LM.Cannabis × Herb.THC × Daily.Interpol.	1.88 × 10^−7^	1.38 × 10^16^	4.79 × 10^10^
	LM.Cannabis × Herb.THC	0.0030	2.55 × 10^13^	1.07 × 10^4^
	Daily.Interpol.	0.0056	9.76 × 10^21^	1.20 × 10^7^
	Tobacco: LM.Cannabis × Resin.THC × Daily.Interpol.	1.36 × 10^−5^	7.48	3.88
				
Lateralization				
	***Additive***			
	Resin	0.0003	186.86	16.72
	LM.Cannabis × Herb.THC × Daily.Interpol.	0.0039	892.53	15.10
	***Interactive***			
	LM.Cannabis × Herb.THC × Daily.Interpol.	0.0061	263.18	9.83
	Tobacco: Resin	1.26 × 10^−5^	75.09	10.63
	***2 Lags***			
	Resin	7.39 × 10^−6^	50.49	13.06
				
				
Teratogenic Syndromes				
	***Additive***			
	LM.Cannabis × Resin.THC × Daily.Interpol.	<2.2 × 10^−16^	356.29	145.49
	***Interactive***			
	LM.Cannabis × Herb.THC × Daily.Interpol.	6.64 × 10^−8^	1.86 × 10^4^	850.04
	Tobacco: LM.Cannabis × Resin.THC × Daily.Interpol.	<2.2 × 10^−16^	2.10	1.95
	***2 Lags***			
	lag(LpmResinDailyInt, 2)	5.52 × 10^−9^	39.30	15.60

Table key: Abbreviations as in Table 2.

**Table 12:** E-Values from Geospatial Models.

Anomaly	Term	*p*-Value	E-Value Estimate	Lower Bound E-Value
				
All Anomalies				
	***Additive***			
	LM.Cannabis × Resin.THC	8.96 × 10^−6^	8.49 × 10^3^	213.85
	***Interactive***			
	Herb	8.09 × 10^−6^	2.87 × 10^4^	1.08 × 10^3^
	LM.Cannabis × Herb.THC × Daily.Interpol.	2.23 × 10^−2^	1.92	1.34
	***2 Lags***			
	LM.Cannabis × Resin.THC	0.0037	18.72	3.60
	LM.Cannabis × Resin.THC × Daily.Interpol.	0.0198	1.65	1.20
				
VACTERL				
	***Additive***			
	Daily.Interpol.	0.0205	2.61 × 10^16^	647.17
	***Interactive***			
	Daily.Interpol.	0.0006	Infinity	2.53 × 10^67^
	***2 Lags***			
	Daily.Interpol.	6.56 × 10^−6^	Infinity	6.52 × 10^138^
	***4 Lags***			
	Daily.Interpol.	6.81 × 10^−12^	Infinity	Infinity
	LM.Cannabis × Herb.THC	0.0022	1.65 × 10^11^	1.76 × 10^4^
	***6 Lags***			
	Daily.Interpol.	8.10 × 10^−10^	Infinity	Infinity
				
FAS				
	***Additive***			
	Herb	0.0004	1.41 × 10^4^	104.35
	***Interactive***			
	Herb	0.0004	1.41 × 10^4^	104.35
	***2 Lags***			
	LM.Cannabis × Resin.THC × Daily.Interpol.	0.0034	23.51	1.53
				
Situs Inversus				
	***Additive***			
	Herb	0.0019	9.61 × 10^4^	107.71
	***Interactive***			
	Herb	0.0019	9.61 × 10^4^	107.71
	***1 Lags***			
	Herb	0.0299	1.05 × 10^4^	4.10
Lateralization				
	***Additive***			
	LM.Cannabis × Resin.THC	0.0006	1.83 × 10^3^	36.69
	LM.Cannabis × Herb.THC × Daily.Interpol.	0.0476	149.75	1.29
	***Interactive***			
	LM.Cannabis × Resin.THC	0.0006	1.83 × 10^3^	36.69
	LM.Cannabis × Herb.THC × Daily.Interpol.	0.0476	149.75	1.29
	***2 Lags***			
	LM.Cannabis × Resin.THC	0.0134	54.61	3.41
				
Teratogenic Syndromes				
	***Additive***			
	Herb	0.0035	1.77 × 10^5^	155.66
	LM.Cannabis × Resin.THC × Daily.Interpol.	0.0003	2.91 × 10^3^	33.76
	***Interactive***			
	Herb	0.0002	7.54 × 10^17^	2.85 × 10^5^
	LM.Cannabis × Resin.THC × Daily.Interpol.	5.65 × 10^−5^	2.63 × 10^3^	29.12
	***2 Lags***			
	LM.Cannabis × Resin.THC × Daily.Interpol.	0.0029	2.02 × 10^5^	21.03
	Herb	0.0137	2.28 × 10^5^	107.43

Table key: Abbreviations as in Table 2.

**Table 13:** E-values List.

No.	E-Value Estimate	Lower Bound E-Value
		
1	Infinity	Infinity
2	Infinity	Infinity
3	Infinity	6.52 × 10^138^
4	Infinity	2.53 × 10^67^
5	3.32 × 10^59^	1.56 × 10^17^
6	5.93 × 10^38^	3.56 × 10^11^
7	9.76 × 10^21^	1.67 × 10^11^
8	1.00 × 10^18^	4.79 × 10^10^
9	7.54 × 10^17^	1.20 × 10^7^
10	2.61 × 10^16^	6.73 × 10^5^
11	1.38 × 10^16^	2.85 × 10^5^
12	4.47 × 10^15^	1.76 × 10^4^
13	2.55 × 10^13^	1.07 × 10^4^
14	1.65 × 10^11^	1.76 × 10^3^
15	7.97 × 10^10^	1.08 × 10^3^
16	2.28 × 10^5^	850.04
17	2.02 × 10^5^	647.17
18	1.77 × 10^5^	213.85
19	9.61 × 10^4^	155.66
20	9.61 × 10^4^	145.49
21	4.29 × 10^4^	107.71
22	2.87 × 10^4^	107.71
23	1.86 × 10^4^	107.43
24	1.57 × 10^4^	106.93
25	1.42 × 10^4^	104.35
26	1.41 × 10^4^	104.35
27	1.41 × 10^4^	72.72
28	1.05 × 10^4^	61.02
29	8.49 × 10^3^	37.93
30	2.91 × 10^3^	36.69
31	2.63 × 10^3^	36.69
32	1.83 × 10^3^	36.32
33	1.83 × 10^3^	33.76
34	892.53	29.12
35	356.29	21.03
36	263.18	16.72
37	230.82	15.60
38	212.22	15.10
39	186.86	13.06
40	149.75	10.63
41	149.75	10.05
42	75.09	9.83
43	73.47	4.74
44	54.61	4.29
45	50.49	4.10
46	50.05	3.88
47	39.30	3.61
48	23.51	3.60
49	18.72	3.41
50	17.04	2.86
51	8.35	2.36
52	7.48	2.31
53	6.65	2.15
54	5.67	1.95
55	4.78	1.62
56	3.01	1.57
57	2.10	1.53
58	1.92	1.45
59	1.77	1.38
60	1.75	1.34
61	1.69	1.29
62	1.65	1.29
63	1.42	1.20
64	1.26	1.18

**Table 14:** E-Values by Congenital Anomaly.

No.	Anomaly	Regression	Model Type	Term	*p*-Value	E-Value Estimate	Lower Bound E-Value
							
1	All Anomalies	Panel	2 Lags	LM.Cannabis × Herb.THC × Daily.Interpol.	0.0176	4.47 × 10^15^	1.76 × 10^3^
2	All Anomalies	Spatial	Interactive	Herb	8.09 × 10^−6^	2.87 × 10^4^	1.08 × 10^3^
3	All Anomalies	Spatial	Additive	LM.Cannabis × Resin.THC	8.96 × 10^−6^	8.49 × 10^3^	213.85
4	All Anomalies	Panel	Interactive	LM.Cannabis × Herb.THC × Daily.Interpol.	<2.2 × 10^−16^	212.22	106.93
5	All Anomalies	Panel	Interactive	LM.Cannabis × Resin.THC × Daily.Interpol.: LM.Cannabis × Herb.THC × Daily.Interpol.	7.81 × 10^−15^	5.67	4.29
6	All Anomalies	Spatial	2 Lags	LM.Cannabis × Resin.THC	0.0037	18.72	3.60
7	All Anomalies	Panel	2 Lags	LM.Cannabis × Resin.THC × Daily.Interpol.: LM.Cannabis × Herb.THC × Daily.Interpol.	0.0303	73.47	2.36
8	All Anomalies	Panel	1 Lag	LM.Cannabis × Resin.THC × Daily.Interpol.: LM.Cannabis × Herb.THC × Daily.Interpol.	<2.2 × 10^−16^	1.69	1.62
9	All Anomalies	Panel	2 Lags	Tobacco: LM.Cannabis × Resin.THC × Daily.Interpol.	2.66 × 10^−8^	1.77	1.57
10	All Anomalies	Panel	Additive	LM.Cannabis × Resin.THC × Daily.Interpol.	0.0001	1.75	1.45
11	All Anomalies	Panel	1 Lag	Tobacco: LM.Cannabis × Resin.THC × Daily.Interpol.	<2.2 × 10^−16^	1.42	1.38
12	All Anomalies	Spatial	Interactive	LM.Cannabis × Herb.THC × Daily.Interpol.	2.23 × 10^−2^	1.92	1.34
13	All Anomalies	Spatial	2 Lags	LM.Cannabis × Resin.THC × Daily.Interpol.	0.0198	1.65	1.20
14	FAS	Panel	2 Lags	LM.Cannabis × Resin.THC × Daily.Interpol.	6.97 × 10^−7^	1.00 × 10^18^	3.56 × 10^11^
15	FAS	Spatial	Additive	Herb	0.0004	1.41 × 10^4^	104.35
16	FAS	Spatial	Interactive	Herb	0.0004	1.41 × 10^4^	104.35
17	FAS	Panel	Interactive	LM.Cannabis × Resin.THC × Daily.Interpol.	0.0020	1.57 × 10^4^	61.02
18	FAS	Panel	2 Lags	Herb	0.0070	4.29 × 10^4^	36.32
19	FAS	Panel	Additive	LM.Cannabis × Resin.THC × Daily.Interpol.	1.44 × 10^−13^	17.04	10.05
20	FAS	Panel	2 Lags	Tobacco: LM.Cannabis × Herb.THC × Daily.Interpol.	1.31 × 10^−5^	6.65	3.61
21	FAS	Panel	Interactive	LM.Cannabis × Resin.THC × Daily.Interpol.: LM.Cannabis × Herb.THC × Daily.Interpol.	0.0016	4.78	2.31
22	FAS	Spatial	2 Lags	LM.Cannabis × Resin.THC × Daily.Interpol.	0.0034	23.51	1.53
23	Lateralization	Panel	Additive	Resin	0.0003	186.86	16.72
24	Lateralization	Panel	Additive	LM.Cannabis × Herb.THC × Daily.Interpol.	0.0039	892.53	15.10
25	Lateralization	Panel	2 Lags	Resin	7.39 × 10^−6^	50.49	13.06
26	Lateralization	Panel	Interactive	Tobacco: Resin	1.26 × 10^−5^	75.09	10.63
27	Lateralization	Panel	Interactive	LM.Cannabis × Herb.THC × Daily.Interpol.	0.0061	263.18	9.83
28	Lateralization	Spatial	Additive	LM.Cannabis × Resin.THC	0.0006	1.83 × 10^3^	36.69
29	Lateralization	Spatial	Interactive	LM.Cannabis × Resin.THC	0.0006	1.83 × 10^3^	36.69
30	Lateralization	Spatial	2 Lags	LM.Cannabis × Resin.THC	0.0134	54.61	3.41
31	Lateralization	Spatial	Additive	LM.Cannabis × Herb.THC × Daily.Interpol.	0.0476	149.75	1.29
32	Lateralization	Spatial	Interactive	LM.Cannabis × Herb.THC × Daily.Interpol.	0.0476	149.75	1.29
33	Situs Inversus	Panel	2 Lags	LM.Cannabis × Herb.THC × Daily.Interpol.	1.88 × 10^−7^	1.38 × 10^16^	4.79 × 10^10^
34	Situs Inversus	Panel	2 Lags	Daily.Interpol.	0.0056	9.76 × 10^21^	1.20 × 10^7^
35	Situs Inversus	Panel	Additive	LM.Cannabis × Herb.THC	5.44 × 10^−6^	7.97 × 10^10^	6.73 × 10^5^
36	Situs Inversus	Panel	2 Lags	LM.Cannabis × Herb.THC	0.0030	2.55 × 10^13^	1.07 × 10^4^
37	Situs Inversus	Spatial	Additive	Herb	0.0019	9.61 × 10^4^	107.71
38	Situs Inversus	Spatial	Interactive	Herb	0.0019	9.61 × 10^4^	107.71
39	Situs Inversus	Panel	Interactive	LM.Cannabis × Herb.THC × Daily.Interpol.	0.0061	50.05	4.74
40	Situs Inversus	Spatial	1 Lags	Herb	0.0299	1.05 × 10^4^	4.10
41	Situs Inversus	Panel	2 Lags	Tobacco: LM.Cannabis × Resin.THC × Daily.Interpol.	1.36 × 10^−5^	7.48	3.88
42	Situs Inversus	Panel	Additive	LM.Cannabis × Herb.THC × Daily.Interpol.	0.0034	8.35	2.86
43	Situs Inversus	Panel	Interactive	Tobacco: Resin	1.26 × 10^−5^	3.01	2.15
44	Teratogenic Syndromes	Spatial	Interactive	Herb	0.0002	7.54 × 10^17^	2.85 × 10^5^
45	Teratogenic Syndromes	Panel	Interactive	LM.Cannabis × Herb.THC × Daily.Interpol.	6.64 × 10^−8^	1.86 × 10^4^	850.04
46	Teratogenic Syndromes	Spatial	Additive	Herb	0.0035	1.77 × 10^5^	155.66
47	Teratogenic Syndromes	Panel	Additive	LM.Cannabis × Resin.THC × Daily.Interpol.	<2.2 × 10^−16^	356.29	145.49
48	Teratogenic Syndromes	Spatial	2 Lags	Herb	0.0137	2.28 × 10^5^	107.43
49	Teratogenic Syndromes	Spatial	Additive	LM.Cannabis × Resin.THC × Daily.Interpol.	0.0003	2.91 × 10^3^	33.76
50	Teratogenic Syndromes	Spatial	Interactive	LM.Cannabis × Resin.THC × Daily.Interpol.	5.65 × 10^−5^	2.63 × 10^3^	29.12
51	Teratogenic Syndromes	Spatial	2 Lags	LM.Cannabis × Resin.THC × Daily.Interpol.	0.0029	2.02 × 10^5^	21.03
52	Teratogenic Syndromes	Panel	2 Lags	lag(LpmResinDailyInt, 2)	5.52 × 10^−9^	39.30	15.60
53	Teratogenic Syndromes	Panel	Interactive	Tobacco: LM.Cannabis × Resin.THC × Daily.Interpol.	<2.2 × 10^−16^	2.10	1.95
54	VACTERL	Spatial	4 Lags	Daily.Interpol.	6.81 × 10^−12^	Infinity	Infinity
55	VACTERL	Spatial	6 Lags	Daily.Interpol.	8.10 × 10^−10^	Infinity	Infinity
56	VACTERL	Spatial	2 Lags	Daily.Interpol.	6.56 × 10^−6^	Infinity	6.52 × 10^138^
57	VACTERL	Spatial	Interactive	Daily.Interpol.	0.0006	Infinity	2.53 × 10^67^
58	VACTERL	Panel	4 Lags	LM.Cannabis × Herb.THC × Daily.Interpol.	0.0009	5.93 × 10^38^	1.56 × 10^17^
59	VACTERL	Panel	1 Lag	Daily.Interpol.	0.0185	3.32 × 10^59^	1.67 × 10^11^
60	VACTERL	Spatial	4 Lags	LM.Cannabis × Herb.THC	0.0022	1.65 × 10^11^	1.76 × 10^4^
61	VACTERL	Spatial	Additive	Daily.Interpol.	0.0205	2.61 × 10^16^	647.17
62	VACTERL	Panel	Additive	LM.Cannabis × Resin.THC × Daily.Interpol.	1.52 × 10^−12^	230.82	72.72
63	VACTERL	Panel	Additive	Herb	0.0042	1.42 × 10^4^	37.93
64	VACTERL	Panel	Interactive	Tobacco: LM.Cannabis × Resin.THC × Daily.Interpol.	4.72 × 10^−5^	1.26	1.18

Table key: Abbreviations as in Table 2.

**Table 15:** Summary of E-values by Congenital Anomaly.

Anomaly	Number	Mean Minimum E-Value	Median Minimum E-Value	Minimum Minimum E-Value	Maximum Minimum E-Value	Mean E-Value Estimate	Median E-Value Estimate	Minimum E-Value Estimate	Maximum E-Value Estimate
VACTERL	11	2.73 × 10^306^	1.67 × 10^11^	1.18	1.50 × 10^307^	5.45 × 10^306^	5.93 × 10^38^	1.26	1.50 × 10^307^
Situs Inversus	11	4.36 × 10^9^	107.71	2.15	4.79 × 10^10^	8.87 × 10^20^	9.61 × 10^4^	3.01	9.76 × 10^21^
Teratogenic Syndromes	10	2.86 × 10^4^	70.595	1.95	285,000	7.54 × 10^16^	10,755.00	2.10	7.54 × 10^17^
FAS	9	3.96 × 10^10^	36.32	1.53	3.56 × 10^11^	1.11 × 10^17^	14,100.00	4.78	1.00 × 10^18^
Lateralization	10	14.471	11.845	1.29	36.69	548.226	168.31	50.49	1830
All Anomalies	13	244.58	2.36	1.2	1760	3.44 × 10^14^	5.67	1.42	4.47 × 10^15^

**Table 16:** E-Values by major cannabis metric group.

No.	Anomaly	Regression	Model Type	Term	Group	*p*-Value	E-Value Estimate	Lower Bound E-Value
								
1	VACTERL	Spatial	4 Lags	Daily.Interpol.	Daily	6.81 × 10^−12^	Infinity	Infinity
2	VACTERL	Spatial	6 Lags	Daily.Interpol.	Daily	8.10 × 10^−10^	Infinity	Infinity
3	VACTERL	Spatial	2 Lags	Daily.Interpol.	Daily	6.56 × 10^−6^	Infinity	6.52 × 10^138^
4	VACTERL	Spatial	Interactive	Daily.Interpol.	Daily	0.0006	Infinity	2.53 × 10^67^
5	VACTERL	Panel	1 Lag	Daily.Interpol.	Daily	0.0185	3.32 × 10^59^	1.67 × 10^11^
6	Situs Inversus	Panel	2 Lags	Daily.Interpol.	Daily	0.0056	9.76 × 10^21^	1.20 × 10^7^
7	VACTERL	Spatial	Additive	Daily.Interpol.	Daily	0.0205	2.61 × 10^16^	647.17
8	Teratogenic Syndromes	Spatial	Interactive	Herb	Herb	0.0002	7.54 × 10^17^	2.85 × 10^5^
9	All Anomalies	Spatial	Interactive	Herb	Herb	8.09 × 10^−6^	2.87 × 10^4^	1.08 × 10^3^
10	Teratogenic Syndromes	Spatial	Additive	Herb	Herb	0.0035	1.77 × 10^5^	155.66
11	Situs Inversus	Spatial	Additive	Herb	Herb	0.0019	9.61 × 10^4^	107.71
12	Situs Inversus	Spatial	Interactive	Herb	Herb	0.0019	9.61 × 10^4^	107.71
13	Teratogenic Syndromes	Spatial	2 Lags	Herb	Herb	0.0137	2.28 × 10^5^	107.43
14	FAS	Spatial	Additive	Herb	Herb	0.0004	1.41 × 10^4^	104.35
15	FAS	Spatial	Interactive	Herb	Herb	0.0004	1.41 × 10^4^	104.35
16	VACTERL	Panel	Additive	Herb	Herb	0.0042	1.42 × 10^4^	37.93
17	FAS	Panel	2 Lags	Herb	Herb	0.0070	4.29 × 10^4^	36.32
18	Situs Inversus	Spatial	1 Lags	Herb	Herb	0.0299	1.05 × 10^4^	4.10
19	Teratogenic Syndromes	Panel	2 Lags	lag (LpmResinDailyInt, 2)	Herb	5.52 × 10^−9^	39.30	15.60
20	Situs Inversus	Panel	Additive	LM.Cannabis × Herb.THC	Herb	5.44 × 10^−6^	7.97 × 10^10^	6.73 × 10^5^
21	VACTERL	Spatial	4 Lags	LM.Cannabis × Herb.THC	Herb	0.0022	1.65 × 10^11^	1.76 × 10^4^
22	Situs Inversus	Panel	2 Lags	LM.Cannabis × Herb.THC	Herb	0.0030	2.55 × 10^13^	1.07 × 10^4^
23	VACTERL	Panel	4 Lags	LM.Cannabis × Herb.THC × Daily.Interpol.	Herb	0.0009	5.93 × 10^38^	1.56 × 10^17^
24	Situs Inversus	Panel	2 Lags	LM.Cannabis × Herb.THC × Daily.Interpol.	Herb	1.88 × 10^−7^	1.38 × 10^16^	4.79 × 10^10^
25	All Anomalies	Panel	2 Lags	LM.Cannabis × Herb.THC × Daily.Interpol.	Herb	0.0176	4.47 × 10^15^	1.76 × 10^3^
26	Teratogenic Syndromes	Panel	Interactive	LM.Cannabis × Herb.THC × Daily.Interpol.	Herb	6.64 × 10^−8^	1.86 × 10^4^	850.04
27	All Anomalies	Panel	Interactive	LM.Cannabis × Herb.THC × Daily.Interpol.	Herb	<2.2 × 10^−16^	212.22	106.93
28	Lateralization	Panel	Additive	LM.Cannabis × Herb.THC × Daily.Interpol.	Herb	0.0039	892.53	15.10
29	Lateralization	Panel	Interactive	LM.Cannabis × Herb.THC × Daily.Interpol.	Herb	0.0061	263.18	9.83
30	Situs Inversus	Panel	Interactive	LM.Cannabis × Herb.THC × Daily.Interpol.	Herb	0.0061	50.05	4.74
31	Situs Inversus	Panel	Additive	LM.Cannabis × Herb.THC × Daily.Interpol.	Herb	0.0034	8.35	2.86
32	All Anomalies	Spatial	Interactive	LM.Cannabis × Herb.THC × Daily.Interpol.	Herb	2.23 × 10^−2^	1.92	1.34
33	Lateralization	Spatial	Additive	LM.Cannabis × Herb.THC × Daily.Interpol.	Herb	0.0476	149.75	1.29
34	Lateralization	Spatial	Interactive	LM.Cannabis × Herb.THC × Daily.Interpol.	Herb	0.0476	149.75	1.29
35	All Anomalies	Spatial	Additive	LM.Cannabis × Resin.THC	Resin	8.96 × 10^−6^	8.49 × 10^3^	213.85
36	Lateralization	Spatial	Additive	LM.Cannabis × Resin.THC	Resin	0.0006	1.83 × 10^3^	36.69
37	Lateralization	Spatial	Interactive	LM.Cannabis × Resin.THC	Resin	0.0006	1.83 × 10^3^	36.69
38	All Anomalies	Spatial	2 Lags	LM.Cannabis × Resin.THC	Resin	0.0037	18.72	3.60
39	Lateralization	Spatial	2 Lags	LM.Cannabis × Resin.THC	Resin	0.0134	54.61	3.41
40	FAS	Panel	2 Lags	LM.Cannabis × Resin.THC × Daily.Interpol.	Resin	6.97 × 10^−7^	1.00 × 10^18^	3.56 × 10^11^
41	Teratogenic Syndromes	Panel	Additive	LM.Cannabis × Resin.THC × Daily.Interpol.	Resin	<2.2 × 10^−16^	356.29	145.49
42	VACTERL	Panel	Additive	LM.Cannabis × Resin.THC × Daily.Interpol.	Resin	1.52 × 10^−12^	230.82	72.72
43	FAS	Panel	Interactive	LM.Cannabis × Resin.THC × Daily.Interpol.	Resin	0.0020	1.57 × 10^4^	61.02
44	Teratogenic Syndromes	Spatial	Additive	LM.Cannabis × Resin.THC × Daily.Interpol.	Resin	0.0003	2.91 × 10^3^	33.76
45	Teratogenic Syndromes	Spatial	Interactive	LM.Cannabis × Resin.THC × Daily.Interpol.	Resin	5.65 × 10^−5^	2.63 × 10^3^	29.12
46	Teratogenic Syndromes	Spatial	2 Lags	LM.Cannabis × Resin.THC × Daily.Interpol.	Resin	0.0029	2.02 × 10^5^	21.03
47	FAS	Panel	Additive	LM.Cannabis × Resin.THC × Daily.Interpol.	Resin	1.44 × 10^−13^	17.04	10.05
48	FAS	Spatial	2 Lags	LM.Cannabis × Resin.THC × Daily.Interpol.	Resin	0.0034	23.51	1.53
49	All Anomalies	Panel	Additive	LM.Cannabis × Resin.THC × Daily.Interpol.	Resin	0.0001	1.75	1.45
50	All Anomalies	Spatial	2 Lags	LM.Cannabis × Resin.THC × Daily.Interpol.	Resin	0.0198	1.65	1.20
51	All Anomalies	Panel	Interactive	LM.Cannabis × Resin.THC × Daily.Interpol.: LM.Cannabis × Herb.THC × Daily.Interpol.	Resin	7.81 × 10^−15^	5.67	4.29
52	All Anomalies	Panel	2 Lags	LM.Cannabis × Resin.THC × Daily.Interpol.: LM.Cannabis × Herb.THC × Daily.Interpol.	Resin	0.0303	73.47	2.36
53	FAS	Panel	Interactive	LM.Cannabis × Resin.THC × Daily.Interpol.: LM.Cannabis × Herb.THC × Daily.Interpol.	Resin	0.0016	4.78	2.31
54	All Anomalies	Panel	1 Lag	LM.Cannabis × Resin.THC × Daily.Interpol.: LM.Cannabis × Herb.THC × Daily.Interpol.	Resin	<2.2 × 10^−16^	1.69	1.62
55	Lateralization	Panel	Additive	Resin	Resin	0.0003	186.86	16.72
56	Lateralization	Panel	2 Lags	Resin	Resin	7.39 × 10^−6^	50.49	13.06
57	FAS	Panel	2 Lags	Tobacco: LM.Cannabis × Herb.THC × Daily.Interpol.	Herb	1.31 × 10^−5^	6.65	3.61
58	Situs Inversus	Panel	2 Lags	Tobacco: LM.Cannabis × Resin.THC × Daily.Interpol.	Resin	1.36 × 10^−5^	7.48	3.88
59	Teratogenic Syndromes	Panel	Interactive	Tobacco: LM.Cannabis × Resin.THC × Daily.Interpol.	Resin	<2.2 × 10^−16^	2.10	1.95
60	All Anomalies	Panel	2 Lags	Tobacco: LM.Cannabis × Resin.THC × Daily.Interpol.	Resin	2.66 × 10^−8^	1.77	1.57
61	All Anomalies	Panel	1 Lag	Tobacco: LM.Cannabis × Resin.THC × Daily.Interpol.	Resin	<2.2 × 10^−16^	1.42	1.38
62	VACTERL	Panel	Interactive	Tobacco: LM.Cannabis × Resin.THC × Daily.Interpol.	Resin	4.72 × 10^−5^	1.26	1.18
63	Lateralization	Panel	Interactive	Tobacco: Resin	Resin	1.26 × 10^−5^	75.09	10.63
64	Situs Inversus	Panel	Interactive	Tobacco: Resin	Resin	1.26 × 10^−5^	3.01	2.15

Table key: Abbreviations as in Table 2.

**Table 17:** Summary of E-values by major cannabis metric group.

Group	Number	Mean Minimum E-Value	Median Minimum E-Value	Minimum Minimum E-Value	Maximum Minimum E-Value	Mean E-Value Estimate	Median E-Value Estimate	Minimum E-Value Estimate	Maximum E-Value Estimate
Daily	7	4.29 × 10^306^	2.53 × 10^67^	647.17	1.50 × 10^307^	8.57 × 10^306^	1.50 × 10^307^	2.61 × 10^16^	1.50 × 10^307^
Herb	28	5.57 × 10^15^	105.64	1.29	1.56 × 10^17^	2.12 × 10^37^	16,400	1.92	5.93 × 10^38^
Resin	29	1.23 × 10^10^	4.29	1.18	3.56 × 10^11^	3.45 × 10^16^	50.49	1.26	1.00 × 10^18^

**Table 18:** Wilcoxson Tests of Intergroup Comparisons Between major cannabis metric groups.

Comparison	W-Statistic	Alternative	*p*-Value
Lower E-Value, Daily_v_Herb	184	two.sided	4.20 × 10^−4^
Lower E-Value, Daily_v_Resin	200	two.sided	8.94 × 10^−5^
Lower E-Value, Herb_v_Resin	592	two.sided	3.06 × 10^−3^
E-Value Estimate, Daily_v_Herb	193	two.sided	9.59 × 10^−5^
E-Value Estimate, Daily_v_Resin	202	two.sided	6.34 × 10^−5^
E-Value Estimate, Herb_v_Resin	642	two.sided	1.70 × 10^−4^

## Data Availability

All data generated or analysed during this study are included in this published article and its supplementary information files. Data along with the relevant R code has been made publicly available on the Mendeley Database Repository and can be accessed from these URLs: https://doi.org/10.17632/tysn37t426.1 and https://doi.org/10.17632/64b9hm8zdn.1 (accessed on 11 December 2022).

## Supplementary Materials

The following supporting information can be downloaded at: https://www.mdpi.com/article/10.3390/pediatric15010009/s1. Supplementary Tables S1–S17, Supplementary Figures S1–S7. List of Supplementary Tables: Supplementary Table S1. Overall Study Profile; Table S2. Daily Cannabis Use-Raw Data; Table S3. Daily Cannabis Use-Interpolated Data; Table S4. All regression slopes and results from bivariate analyses; Table S5. Variable Importance Tables from Ranger random forest regression-All anomalies; Table S6. Variable Importance Tables from Ranger random forest regression-VATER/VACTERL; Table S7. Variable Importance Tables from Ranger random forest regression-Fetal alcohol syndrome; Table S8. Variable Importance Tables from Ranger random forest regression-Situs inversus; Table S9. Variable Importance Tables from Ranger random forest regression–Lateralization; Table S10. Variable Importance Tables from Ranger random forest regression-Teratogenic syndromes; Table S11. Inverse probability weighted panel regression results-All anomalies; Table S12. Inverse probability weighted panel regression results-VATER/VACTERL; Table S13. Inverse probability weighted panel regression results-Fetal alcohol syndrome; Table S14. Inverse probability weighted panel regression results-Situs inversus; Table S15. Inverse probability weighted panel regression results–Lateralization; Table S16. Inverse probability weighted panel regression results-Teratogenic syndromes; Table S17. All E-Values by descending minimum E-Value. List of Supplementary Figures: Figure S1. Sequential map-graph of the log rate of Foetal Alcohol Syndrome over time in selected European nations; Figure S2. Sequential map-graph of the log rate of Skeletal Dysplasia over time in selected European nations; Figure S3. Sequential map-graph of the log rate of past month cannabis use × cannabis resin THC concentration × daily cannabis use interpolated over time in selected European nations; Figure S4. Bivariate colorplane sequential map-graph of the log rate of Foetal Alcohol Syndrome by last month cannabis use: cannabis resin THC concentration: daily cannabis use interpolated in selected European nations; Figure S5. Bivariate colorplane sequential map-graph of the log rate of Skeletal Dysplasia by last month cannabis use: cannabis resin THC concentration: daily cannabis use interpolated in selected European nations; Figure S6. Bivariate colorplane sequential map-graph of the log rate of Maternal Infections Anomalies by last month cannabis use: cannabis resin THC concentration: daily cannabis use interpolated in selected European nations; Figure S7. International geospatial links (A) raw (in blue) and edited (in red) and (B) final (in pink) used to derive the sparse spatial weights matrix for spatial regression.
