# Supplementary material for: Patterns of Cannabis- and Substance-Related Congenital General Anomalies in Europe: A Geospatiotemporal and Causal Inferential Study
Source: Pediatr Rep. 2023 Feb 7;15(1):69–118. doi: 10.3390/pediatric15010009 (PMC9944887; doi:10.3390/pediatric15010009)
Supplement: Supplementary file 1 [file pediatrrep-15-00009-s001.zip › pediatrrep-2159926-supplementary.pdf]

SUPPLEMENTARY TABLES

Table of Contents

Supplementary Table Number	Contents
1	Overall Study Profile
2	Daily Cannabis Use—Raw Data
3	Daily Cannabis Use—Interpolated Data
4	All regression slopes and results from bivariate analyses
5	Variable Importance Tables from Ranger random forest regression—All anomalies
6	Variable Importance Tables from Ranger random forest regression—VATER/VACTERL
7	Variable Importance Tables from Ranger random forest regression—Fetal alcohol syndrome
8	Variable Importance Tables from Ranger random forest regression—Situs inversus
9	Variable Importance Tables from Ranger random forest regression—Lateralization
10	Variable Importance Tables from Ranger random forest regression—Teratogenic syndromes
11	Inverse probability weighted panel regression results—All anomalies
12	Inverse probability weighted panel regression results—VATER/VACTERL
13	Inverse probability weighted panel regression results—Fetal alcohol syndrome
14	Inverse probability weighted panel regression results—Situs inversus
15	Inverse probability weighted panel regression results—Lateralization
16	Inverse probability weighted panel regression results—Teratogenic syndromes
17	All E-Values by descending minimum E-Value

Table S1. Overall Study Profile

Variable	Value
Sample Size	1312
Country (%)	
Belgium	110 (8.4)
Bulgaria	110 (8.4)
Croatia	88 (6.7)
Finland	45 (3.4)
France	110 (8.4)
Germany	110 (8.4)
Hungary	27 (2.1)
Italy	110 (8.4)
Netherlands	99 (7.5)
Norway	110 (8.4)
Poland	110 (8.4)
Portugal	110 (8.4)
Spain	110 (8.4)
Sweden	63 (4.8)
Year (Range)	2010: 2019
Anomaly (%)	
All Anomalies	122 (9.3)
Congenital constriction bands/amniotic band	122 (9.3)
Conjoined twins	122 (9.3)
Fetal alcohol syndrome	122 (9.3)
Lateral anomalies	107 (8.2)
Maternal infections resulting in malformations	122 (9.3)
Situs inversus	122 (9.3)
Skeletal dysplasias	122 (9.3)
Teratogenic syndromes with malformations	122 (9.3)
Valproate syndrome	122 (9.3)
VATER/VACTERL	107 (8.2)
Anomaly_Rate (mean (SD))	24.39 (79.61)
Tobacco (mean (SD))	22.80 (5.47)
Alcohol (p.c.L/Yr) (mean (SD))	10.77 (1.83)
Amphetamines (% Use) (median [IQR])	0.60 [0.28, 0.80]
Cocaine (% Use) (median [IQR])	0.73 [0.40, 1.20]
Last_Month_Cannabis (% Use) (mean (SD))	0.04 (0.02)
Cannabis_Herb_THC_Content (%) (mean (SD))	0.09 (0.03)
Cannabis_Resin_THC_Content (%) (mean (SD))	0.16 (0.09)
%_Daily_Cannabis_ Use (mean (SD))	0.01 (0.01)
%_Daily_Cannabis_Use_Interpolated (mean (SD))	0.01 (0.01)
Last_Month_Cannabis_x_Herb_THC_Content (mean (SD))	0.04 (0.03)
Last_Month_Cannabis_x_Resin_THC_Content (mean (SD))	0.13 (0.12)
Last_Month_Cannabis_x_Herb_THC_Content_x_Daily_Use_Interpolated (median [IQR])	0.02 [0.00, 0.06]
Last_Month_Cannabis_x_Resin_THC_Content_x_Daily_Use_Interpolated (median [IQR])	0.03 [0.01, 0.13]
Trend_in_Daily_Cannabis_Use = Increasing (%)	957 (72.9)
Mean_Annual_Household_Income (mean (SD))	30125.34 (17333.45)

Table S2. Daily Cannabis Use—Raw Data

Country	2010	2011	2012	2013	2014	2015	2016	2017	2018	2019
Belgium	—	—	—	0.0054	—	—	—	—	0.0132	—
Bulgaria	—	—	0.0020	—	—	—	0.0016	—	—	—
Croatia	—	—	0.0050	—	—	0.0078	—	—	—	—
Finland	0.0020	—	—	—	0.0031	—	—	—	—	—
France	0.0150	—	—	—	0.0217	—	—	0.0199	—	—
Germany	—	—	0.0060	—	—	0.0067	—	—	0.0084	—
Hungary	—	—	—	—	—	—	—	—	—	—
Italy	—	—	0.0030	—	0.0094	—	—	0.0108	—	—
Netherlands	—	0.0146	0.0146	0.0146	0.0146	0.0146	0.0133	0.0126	0.0190	0.0200
Norway	0.0030	0.0030	0.0030	0.0030	0.0037	0.0044	0.0053	0.0016	0.0068	0.0038
Poland	0.0040	—	0.0010	—	0.0018	—	—	—	—	—
Portugal	—	—	0.0050	—	—	—	0.0002	—	—	—
Spain	—	0.0250	—	0.0260	—	0.0285	—	0.0372	—	—
Sweden	—	—	—	—	—	—	—	—	—	—

Table S3. Daily Cannabis Use – Interpolated Data

Country	2010	2011	2012	2013	2014	2015	2016	2017	2018	2019
Belgium	0.0054	0.0054	0.0054	0.0054	0.0070	0.0085	0.0101	0.0116	0.0132	0.0132
Bulgaria	0.0020	0.0020	0.0020	0.0019	0.0018	0.0017	0.0016	0.0016	0.0016	0.0016
Croatia	0.0050	0.0050	0.0050	0.0059	0.0069	0.0078	0.0092	0.0107	—	—
Finland	0.0020	0.0023	0.0026	0.0028	0.0031	—	—	—	—	—
France	0.0150	0.0167	0.0184	0.0200	0.0217	0.0211	0.0205	0.0199	0.0199	0.0199
Germany	0.0060	0.0060	0.0060	0.0062	0.0065	0.0067	0.0073	0.0078	0.0084	0.0084
Hungary	0.0007	0.0007	0.0007	—	—	—	—	—	—	—
Italy	0.0030	0.0030	0.0030	0.0062	0.0094	0.0099	0.0103	0.0108	0.0108	0.0108
Netherlands	—	0.0146	0.0146	0.0146	0.0146	0.0146	0.0133	0.0126	0.0190	0.0200
Norway	0.0030	0.0030	0.0030	0.0030	0.0037	0.0044	0.0053	0.0016	0.0068	0.0038
Poland	0.0040	0.0025	0.0010	0.0014	0.0018	0.0018	0.0018	0.0018	0.0018	0.0018
Portugal	0.0050	0.0050	0.0050	0.0038	0.0026	0.0014	0.0002	0.0002	0.0002	0.0002
Spain	0.0250	0.0250	0.0255	0.0260	0.0273	0.0285	0.0329	0.0372	0.0372	0.0372
Sweden	—	—	—	—	—	—	—	—	—	—

Table S4. All Regression Slopes And Results From Bivariate Analyses

Anomaly	Substance	Mean Anomaly Rate	Estimate	Std.Error	Sigma	t_statistic	P_Value	E-Value Estimate	E-Value Lower Bound
VATER/VACTERL	Daily.Interpol.	0.4377	20.8858	4.6484	0.4252	4.4932	1.81E-05	5.19E+19	1.83E+11
Teratogenic Synds	Daily.Interpol.	1.0683	29.9733	6.8274	0.6408	4.3901	2.56E-05	6.10E+18	3.54E+10
Lateral anomalies	Daily.Interpol.	1.7332	26.6028	6.7914	0.6212	3.9172	1.60E-04	1.68E+17	5.95E+08
Matern Infect Malform	Daily.Interpol.	0.6880	22.6665	5.8194	0.5462	3.8950	1.67E-04	5.02E+16	2.92E+08
Teratogenic Synds	LMCannabis_Herb	1.0683	11.1368	2.1817	0.6263	5.1046	1.26E-06	2.13E+07	4.32E+04
Situs inversus	Daily.Interpol.	0.5932	12.3967	4.2919	0.4029	2.8884	0.0046	2.90E+12	1.68E+04
Lateral anomalies	LMCannabis_Herb	1.7332	10.0481	2.3741	0.6147	4.2324	4.96E-05	5.78E+06	5.97E+03
Matern Infect Malform	LMCannabis_Herb	0.6880	8.2647	1.8585	0.5335	4.4469	1.96E-05	2.65E+06	5.38E+03
Skeletal dysplasias	Daily.Interpol.	1.8050	19.3566	7.0728	0.6639	2.7368	0.0072	6.67E+11	3.87E+03
Lateral anomalies	Herb	1.7332	8.3890	1.8336	0.6072	4.5752	1.31E-05	5.76E+05	2.67E+03
Teratogenic Synds	Herb	1.0683	7.7193	1.6312	0.6343	4.7322	6.13E-06	1.29E+05	1.33E+03
Matern Infect Malform	Herb	0.6880	6.2822	1.3652	0.5308	4.6016	1.05E-05	9.51E+04	976.69
Situs inversus	LMCannabis_Herb	0.5932	5.1389	1.3379	0.3840	3.8410	1.97E-04	3.88E+05	786.86
Situs inversus	Herb	0.5932	4.0862	0.9779	0.3802	4.1784	5.60E-05	3.53E+04	362.44
Fetal Alcohol	LMCannabis_Herb	0.2458	4.3930	1.2221	0.3508	3.5945	4.73E-04	1.78E+05	359.95
VATER/VACTERL	LM_Cannabis	0.4377	6.5421	2.0669	0.4436	3.1652	0.0020	1.35E+06	336.59
Teratogenic Synds	LM_Cannabis	1.0683	8.8997	2.7367	0.6624	3.2519	0.0015	4.09E+05	260.80
Lateral anomalies	LM_Cannabis	1.7332	8.9858	2.9721	0.6378	3.0234	0.0031	7.39E+05	184.22
VATER/VACTERL	LMCannabis_Herb	0.4377	5.1942	1.7200	0.4453	3.0199	0.0032	8.14E+04	83.64
Matern Infect Malform	LM.Cannabis_x_Herb.THC: LM.Cannabis_x_Resin.THC_x_Daily.Interpol.	0.6880	6.4725	2.3045	0.5577	2.8087	0.0058	7.72E+04	48.85
Skeletal dysplasias	LMCannabis_Herb	1.8050	6.5116	2.2481	0.6453	2.8965	0.0045	1.94E+04	38.92

Teratogenic Synds	LM.Cannabis_x_Herb.THC_x_Daily.Interpol.	1.0683	3.1437	0.7564	0.6457	4.1562	6.32E-05	167.39	20.36
Lateral anomalies	Resin	1.7332	3.0137	0.7391	0.6363	4.0773	9.46E-05	148.33	18.31
Fetal Alcohol	Herb	0.2458	2.6533	0.9181	0.3570	2.8898	0.0046	1.73E+03	17.27
Matern Infect Malform	LM.Cannabis_x_Herb.THC_x_Daily.Interpol.	0.6880	2.3897	0.6433	0.5492	3.7149	3.18E-04	104.39	12.52
Lateral anomalies	LM.Cannabis_x_Herb.THC_x_Daily.Interpol.	1.7332	2.4982	0.7511	0.6325	3.3259	0.0012	72.26	8.26
All Anomalies	Resin	255.4744	1.2426	0.3661	0.3291	3.3942	9.72E-04	61.62	8.04
Fetal Alcohol	Daily.Interpol.	0.2458	8.2930	3.9391	0.3697	2.1053	0.0375	1.46E+09	7.97
Lateral anomalies	LMCannabis_Resin	1.7332	2.1164	0.5631	0.6436	3.7586	2.95E-04	39.37	7.87
Skeletal dysplasias	Herb	1.8050	4.2006	1.6733	0.6506	2.5103	0.0134	711.51	6.78
Amniotic band	LMCannabis_Resin	0.3730	1.0556	0.3089	0.3699	3.4170	9.02E-04	26.33	5.52
Skeletal dysplasias	LM.Cannabis_x_Herb.THC_x_Daily.Interpol.	1.8050	2.2295	0.7751	0.6617	2.8764	0.0048	42.40	4.78
Situs inversus	LM.Cannabis_x_Herb.THC_x_Daily.Interpol.	0.5932	1.3525	0.4721	0.4031	2.8647	0.0050	41.87	4.71
Teratogenic Synds	LMCannabis_Resin	1.0683	1.8066	0.5687	0.6810	3.1768	0.0020	21.85	4.49
Fetal Alcohol	LM.Cannabis_x_Herb.THC_x_Daily.Interpol.	0.2458	1.1590	0.4278	0.3653	2.7090	0.0078	35.39	3.89
Teratogenic Synds	LM.Cannabis_x_Resin.THC_x_Daily.Interpol.	1.0683	1.2927	0.3585	0.6708	3.6062	4.91E-04	11.03	3.89
Fetal Alcohol	LMCannabis_Resin	0.2458	0.8989	0.3077	0.3684	2.9213	0.0043	17.90	3.58
Matern Infect Malform	LMCannabis_Resin	0.6880	1.3658	0.4763	0.5703	2.8674	0.0050	17.16	3.41
All Anomalies	Herb	255.4744	2.2466	1.0012	0.3893	2.2438	0.0267	381.13	3.33
Matern Infect Malform	LM.Cannabis_x_Resin.THC_x_Daily.Interpol.	0.6880	0.9847	0.3031	0.5672	3.2483	0.0016	9.18	3.16
VATER/VACTERL	LM.Cannabis_x_Herb.THC_x_Daily.Interpol.	0.4377	1.3541	0.5352	0.4507	2.5300	0.0129	30.28	3.12
Lateral anomalies	LM.Cannabis_x_Resin.THC_x_Daily.Interpol.	1.7332	1.1244	0.3521	0.6555	3.1934	0.0019	9.00	3.06
Amniotic band	LMCannabis_Herb	0.3730	3.2048	1.4989	0.4302	2.1381	0.0345	1.76E+03	2.96

VATER/VACTERL	Cocaine	0.4377	0.3393	0.0527	0.3931	6.4369	3.72E-09	3.81	2.85
Skeletal dysplasias	LMCannabis_Resin	1.8050	1.3870	0.5266	0.6306	2.6337	0.0097	14.28	2.74
Skeletal dysplasias	LM.Cannabis_x_Resin.THC_x_Daily.Interpol.	1.8050	1.0055	0.3440	0.6436	2.9233	0.0043	7.75	2.58
All Anomalies	LMCannabis_Resin	255.4744	0.7027	0.2813	0.3368	2.4982	0.0140	12.83	2.39
All Anomalies	LM.Cannabis_x_Resin.THC_x_Daily.Interpol.	255.4744	0.4992	0.1811	0.3388	2.7572	0.0070	7.11	2.32
Matern Infect Malform	Cocaine	0.6880	0.3460	0.0644	0.5169	5.3762	3.80E-07	3.08	2.31
Fetal Alcohol	LM.Cannabis_x_Resin.THC_x_Daily.Interpol.	0.2458	0.5533	0.2024	0.3787	2.7344	0.0074	7.02	2.28
Teratogenic Synds	Cocaine	1.0683	0.3971	0.0780	0.6266	5.0903	1.34E-06	2.96	2.21
Amniotic band	LM.Cannabis_x_Resin.THC_x_Daily.Interpol.	0.3730	0.5439	0.2053	0.3842	2.6491	0.0094	6.71	2.15
VATER/VACTERL	LMCannabis_Resin	0.4377	0.9512	0.4034	0.4610	2.3582	0.0204	12.55	2.10
Lateral anomalies	Cocaine	1.7332	0.3713	0.0815	0.6077	4.5565	1.41E-05	2.88	2.09
VATER/VACTERL	LM.Cannabis_x_Resin.THC_x_Daily.Interpol.	0.4377	0.6332	0.2464	0.4587	2.5702	0.0117	6.48	2.04
Situs inversus	LM.Cannabis_x_Resin.THC_x_Daily.Interpol.	0.5932	0.5669	0.2250	0.4211	2.5194	0.0134	6.27	1.96
Situs inversus	Cocaine	0.5932	0.2054	0.0471	0.3781	4.3619	2.74E-05	2.66	1.95
Situs inversus	LMCannabis_Resin	0.5932	0.7543	0.3444	0.4123	2.1905	0.0307	10.04	1.68
Skeletal dysplasias	Cocaine	1.8050	0.2686	0.0794	0.6378	3.3820	9.72E-04	2.29	1.63
All Anomalies	Cocaine	255.4744	0.1491	0.0476	0.3821	3.1349	0.0022	2.21	1.55
Fetal Alcohol	Annual_Alcohol	0.2458	0.0793	0.0169	0.3393	4.6992	7.02E-06	1.78	1.52
All Anomalies	Daily.Interpol.	255.4744	0.1426	0.0481	0.3836	2.9645	0.0037	2.15	1.49
VATER/VACTERL	Log(Amphetamine)	0.4377	0.1506	0.0582	0.4501	2.5891	0.0110	2.05	1.37
Amniotic band	Annual_Alcohol	0.3730	0.0709	0.0208	0.4186	3.4067	8.95E-04	1.61	1.34
Teratogenic Synds	Annual_Alcohol	1.0683	0.0942	0.0333	0.6690	2.8296	0.0055	1.53	1.25
Valproate syndrome	Cocaine	0.0434	0.0362	0.0169	0.1354	2.1472	0.0338	1.87	1.17
All Anomalies	LM.Cannabis_x_Herb.THC_x_Daily.Interpol.	255.4744	0.9163	0.4650	0.3970	1.9705	0.0512	15.82	1.14
Valproate syndrome	Annual_Alcohol	0.0434	0.0030	0.0069	0.1379	0.4324	0.6662	1.16	1

Amniotic band	Log(Amphetamine)	0.3730	0.0095	0.0550	0.4383	0.1737	0.8624	1.16	1
Skeletal dysplasias	Annual_Alcohol	1.8050	0.0161	0.0332	0.6669	0.4851	0.6285	1.17	1
Conjoined twins	Annual_Alcohol	0.1162	0.0051	0.0097	0.1943	0.5277	0.5987	1.18	1
Fetal Alcohol	Tobacco	0.2458	0.0099	0.0061	0.3652	1.6225	0.1073	1.18	1
Lateral anomalies	Annual_Alcohol	1.7332	0.0184	0.0342	0.6641	0.5371	0.5923	1.19	1
Matern Infect Malform	Annual_Alcohol	0.6880	0.0286	0.0285	0.5734	1.0030	0.3179	1.27	1
Lateral anomalies	Log(Amphetamine)	1.7332	0.0345	0.0859	0.6645	0.4019	0.6886	1.27	1
Amniotic band	Cocaine	0.3730	0.0236	0.0545	0.4380	0.4332	0.6656	1.28	1
Skeletal dysplasias	Log(Amphetamine)	1.8050	0.0459	0.0836	0.6667	0.5487	0.5842	1.33	1
VATER/VACTERL	Annual_Alcohol	0.4377	0.0385	0.0236	0.4585	1.6321	0.1057	1.37	1
Conjoined twins	Log(Amphetamine)	0.1162	0.0202	0.0243	0.1940	0.8296	0.4084	1.43	1
Fetal Alcohol	Cocaine	0.2458	0.0423	0.0458	0.3679	0.9242	0.3573	1.46	1
Conjoined twins	Cocaine	0.1162	0.0268	0.0241	0.1936	1.1102	0.2691	1.52	1
Valproate syndrome	Resin	0.0434	0.0316	0.1591	0.1430	0.1986	0.8430	1.74	1
Valproate syndrome	Log(Amphetamine)	0.0434	0.0313	0.0171	0.1361	1.8352	0.0690	1.77	1
Conjoined twins	LM.Cannabis_x_Herb.THC_x_Daily.Interpol.	0.1162	0.0665	0.2326	0.1986	0.2860	0.7754	2.05	1
Conjoined twins	LM.Cannabis_x_Resin.THC_x_Daily.Interpol.	0.1162	0.0762	0.1075	0.2011	0.7088	0.4802	2.17	1
Valproate syndrome	LM.Cannabis_x_Resin.THC_x_Daily.Interpol.	0.0434	0.0594	0.0787	0.1472	0.7556	0.4517	2.24	1
Conjoined twins	Resin	0.1162	0.0856	0.2188	0.1967	0.3912	0.6964	2.34	1
Conjoined twins	LMCannabis_Resin	0.1162	0.0866	0.1641	0.1965	0.5278	0.5988	2.35	1
Valproate syndrome	LM.Cannabis_x_Herb.THC_x_Daily.Interpol.	0.0434	0.0642	0.1660	0.1417	0.3869	0.6996	2.39	1
VATER/VACTERL	Resin	0.4377	0.2796	0.5502	0.4737	0.5082	0.6125	2.81	1
Valproate syndrome	LMCannabis_Resin	0.0434	0.1069	0.1190	0.1425	0.8985	0.3710	3.37	1
Conjoined twins	LMCannabis_Herb	0.1162	0.1460	0.6776	0.1945	0.2155	0.8298	3.37	1
Conjoined twins	Herb	0.1162	0.2175	0.4999	0.1944	0.4350	0.6643	4.98	1
Amniotic band	Herb	0.3730	0.5226	1.1264	0.4380	0.4640	0.6435	5.37	1
Amniotic band	Resin	0.3730	0.4708	0.4314	0.3878	1.0915	0.2776	5.49	1
Valproate syndrome	LMCannabis_Herb	0.0434	0.1800	0.4805	0.1379	0.3746	0.7086	6.01	1

Teratogenic Synds	Resin	1.0683	0.9295	0.7879	0.7083	1.1797	0.2408	6.06	1
Fetal Alcohol	Resin	0.2458	0.5007	0.4234	0.3806	1.1826	0.2396	6.08	1
Situs inversus	Resin	0.5932	0.7215	0.4637	0.4169	1.5557	0.1228	9.13	1
Matern Infect Malform	Resin	0.6880	1.0193	0.6513	0.5855	1.5651	0.1206	9.22	1
Amniotic band	LM.Cannabis_x_Herb.THC_x_Daily.Interpol.	0.3730	0.8282	0.5165	0.4409	1.6036	0.1116	10.52	1
Skeletal dysplasias	Resin	1.8050	1.3411	0.7124	0.6404	1.8827	0.0625	12.93	1
Valproate syndrome	LM_Cannabis	0.0434	0.5334	0.5681	0.1375	0.9388	0.3497	67.75	1
VATER/VACTERL	Herb	0.4377	2.2705	1.3842	0.4584	1.6403	0.1039	180.82	1
All Anomalies	LMCannabis_Herb	255.4744	2.3984	1.3670	0.3924	1.7545	0.0819	520.23	1
Amniotic band	LM_Cannabis	0.3730	2.9224	1.7915	0.4336	1.6312	0.1055	921.49	1
Skeletal dysplasias	LM_Cannabis	1.8050	4.5038	2.7272	0.6601	1.6514	0.1013	994.22	1
Conjoined twins	Daily.Interpol.	0.1162	1.3580	2.1129	0.1983	0.6427	0.5217	1.02E+03	1
Situs inversus	LM_Cannabis	0.5932	2.8790	1.6609	0.4020	1.7334	0.0856	1.35E+03	1
Fetal Alcohol	LM_Cannabis	0.2458	2.8516	1.5032	0.3638	1.8970	0.0602	2.50E+03	1
Amniotic band	Daily.Interpol.	0.3730	6.6092	4.7097	0.4421	1.4033	0.1633	1.62E+06	1
Valproate syndrome	Daily.Interpol.	0.0434	2.2712	1.4958	0.1404	1.5184	0.1317	4.94E+06	1
All Anomalies	Daily.Interpol.	255.4744	6.4900	4.2581	0.3997	1.5242	0.1303	5.23E+06	1
Valproate syndrome	Tobacco	0.0434	-0.0020	0.0023	0.1376	-0.8538	0.3949	1.13	NA
Skeletal dysplasias	Tobacco	1.8050	-0.0105	0.0111	0.6650	-0.9518	0.3431	1.14	NA
Amniotic band	Tobacco	0.3730	-0.0076	0.0073	0.4364	-1.0402	0.3004	1.14	NA
Teratogenic Synds	Tobacco	1.0683	-0.0121	0.0114	0.6877	-1.0563	0.2930	1.14	NA
All Anomalies	Tobacco	255.4744	-0.0077	0.0066	0.3952	-1.1649	0.2464	1.15	NA
Conjoined twins	Tobacco	0.1162	-0.0039	0.0032	0.1934	-1.2046	0.2307	1.16	NA
All Anomalies	Annual_Alcohol	255.4744	-0.0082	0.0198	0.3971	-0.4126	0.6806	1.16	NA
VATER/VACTERL	Tobacco	0.4377	-0.0105	0.0080	0.4605	-1.3106	0.1929	1.17	NA
Lateral anomalies	Tobacco	1.7332	-0.0197	0.0114	0.6557	-1.7320	0.0862	1.20	NA
Matern Infect Malform	Resin	0.6880	-0.0186	0.0094	0.5666	-1.9760	0.0505	1.21	NA
Situs inversus	Annual_Alcohol	0.5932	-0.0246	0.0201	0.4045	-1.2210	0.2245	1.30	NA
Situs inversus	Tobacco	0.5932	-0.0237	0.0064	0.3855	-3.7024	3.24E-04	1.30	NA
Fetal Alcohol	Log(Amphetamine)	0.2458	-0.0265	0.0462	0.3687	-0.5728	0.5679	1.34	NA

Teratogenic Synds	Log(Amphetamine)	1.0683	-0.0580	0.0865	0.6896	-0.6704	0.5039	1.37	NA
Matern Infect Malform	Log(Amphetamine)	0.6880	-0.0815	0.0718	0.5727	-1.1348	0.2587	1.53	NA
Situs inversus	Log(Amphetamine)	0.5932	-0.1247	0.0497	0.3967	-2.5064	0.0135	1.99	NA
Conjoined twins	LM_Cannabis	0.1162	-0.2036	0.8036	0.1945	-0.2534	0.8004	4.63	NA
Valproate syndrome	Herb	0.0434	-0.1677	0.3546	0.1379	-0.4728	0.6372	5.50	NA
All Anomalies	LM_Cannabis	255.4744	-0.5886	1.6411	0.3972	-0.3587	0.7205	7.17	NA

Table S5. Random Forrest Additive Regression Table for All Anomalies

Variable	Importance
Income	3.4546
LM.Cannabis_x_Resin.THC_x_Daily.Interpol.	2.1885
LM.Cannabis_x_Herb.THC_x_Daily.Interpol.	1.8197
Daily.Interpol.	1.5029
Tobacco	1.4966
LM.Cannabis_x_Resin.THC	1.2614
Herb	1.2395
Amphetamines	1.1972
Alcohol	1.1359
LM.Cannabis_x_Herb.THC	0.9637
Daily.Interpol.	0.9284
LM.Cannabis	0.7007
Resin	0.5942

Table S6. Random Forrest Additive Regression Table for VACTERL Syndrome

Variable	Importance
Income	3.7952
LM.Cannabis_x_Herb.THC_x_Daily.Interpol.	3.0873
Daily.Interpol.	2.4974
Cocaine	1.8505
LM.Cannabis_x_Resin.THC_x_Daily.Interpol.	1.7959
Alcohol	1.6821
Tobacco	1.3963
Amphetamines	1.2005
LM.Cannabis	0.9885
LM.Cannabis_x_Herb.THC	0.9260
Herb	0.8642
Resin	0.7581
LM.Cannabis_x_Resin.THC	0.7403

Table S7. Random Forrest Additive Regression Table for Foetal Alcohol Syndrome

Variable	Importance
Alcohol	3.0384
LM.Cannabis_x_Herb.THC_x_Daily.Interpol.	1.7490
Income	1.4107
LM.Cannabis_x_Resin.THC_x_Daily.Interpol.	1.1661
Daily.Interpol.	1.0496
Herb	1.0178
Cocaine	0.9861
Tobacco	0.9759
LM.Cannabis_x_Herb.THC	0.9156
LM.Cannabis_x_Resin.THC	0.7670
Resin	0.7396
Amphetamines	0.6570
LM.Cannabis	0.6049

Table S8. Random Forrest Additive Regression Table for Situs Inversus

Variable	Importance
Income	3.9593
Tobacco	2.4043
Cocaine	1.6454
LM.Cannabis_x_Herb.THC_x_Daily.Interpol.	1.5474
Herb	1.4380
LM.Cannabis_x_Resin.THC_x_Daily.Interpol.	1.4340
Amphetamines	1.1595
LM.Cannabis_x_Herb.THC	1.1307
Alcohol	1.0694
LM.Cannabis_x_Resin.THC	0.8533
LM.Cannabis_x_Herb.THC: LM.Cannabis_x_Resin.THC_x_Daily.Interpol.	0.8139
LM.Cannabis	0.7935
Resin	0.5965

Table S9. Random Forrest Additive Regression Table for Lateralization Anomalies

Variable	Importance
Resin	7.5604
LM.Cannabis_x_Resin.THC_x_Daily.Interpol.	4.6247
Resin	4.1062
Tobacco	4.0939
LM.Cannabis_x_Herb.THC_x_Daily.Interpol.	3.5151
LM.Cannabis_x_Resin.THC	3.4361
Daily.Interpol.	3.1644
LM.Cannabis_x_Herb.THC	3.0207
Herb	2.8943
Alcohol	2.6885
Cocaine	2.2928
LM.Cannabis	1.3929
Amphetamines	1.2167

Table S10. Random Forrest Additive Regression Table for Teratogenic Syndromes

Variable	Importance
Income	9.0388
LM.Cannabis_x_Herb.THC_x_Daily.Interpol.	6.9946
Alcohol	5.7832
LM.Cannabis_x_Resin.THC_x_Daily.Interpol.	5.5778
Tobacco	4.9817
Herb	4.3483
Daily.Interpol.	4.0041
Cocaine	3.4548
LM.Cannabis_x_Herb.THC	2.4145
LM.Cannabis_x_Resin.THC	2.3917
Resin	2.1607
LM.Cannabis	1.8638
Amphetamines	1.6147

Table S11. Final Interactive Inverse Probability Weighted Panel Regression Models for All Anomalies

Parameters			Model Parameters	
**Term**	**Estimate (C.I.)**	**P-Value**	**Parameter**	**Value**
Additive				
(LAnomRt ~ TobRt + AlcRt + LpmHerbDailyInt + LpmResinDailyInt + pm.Daily.Intpltd + pmResin + LAmph + LCocc + MHY)				
LM.Cannabis_x_Resin.THC_x_Daily.Interpol.	0.12 (0.06, 0.19)	0.0001	Adj.R.Squared	0.0032
Income	0 (0, 0)	3.66E-05	Statistic	14.2766
			Deg.Freedom	2,119
			P-Value	2.77E-06
Interactive				
Rate ~ Tobacco * LM.Cannabis_x_Resin.THC_x_Daily.Interpol. * LM.Cannabis_x_Herb.THC_x_Daily.Interpol. + Alcohol + Amphetamines + Cocaine + Income				
Tobacco	0.06 (0.03, 0.08)	1.25E-05	Adj.R.Squared	0.1471
LM.Cannabis_x_Herb.THC_x_Daily.Interpol.	1.84 (1.57, 2.11)	<2.2E-16	Statistic	29.4698
Alcohol	-0.09 (-0.13, -0.04)	0.0006	Deg.Freedom	8,113
Amphetamines	0.11 (0, 0.21)	0.0433	P-Value	<2.2E-16
Cocaine	-0.28 (-0.35, -0.2)	4.39E-10		
Income	0 (0, 0)	3.10E-08		
LM.Cannabis_x_Resin.THC_x_Daily.Interpol.: LM.Cannabis_x_Herb.THC_x_Daily.Interpol.	0.45 (0.35, 0.54)	7.81E-15		
Tobacco: LM.Cannabis_x_Resin.THC_x_Daily.Interpol.: LM.Cannabis_x_Herb.THC_x_Daily.Interpol.	-0.01 (-0.02, -0.01)	1.84E-08		
1 Lag				
Rate ~ Tobacco * LM.Cannabis_x_Resin.THC_x_Daily.Interpol. * LM.Cannabis_x_Herb.THC_x_Daily.Interpol. + Alcohol+ Amphetamines + Cocaine + Income				
Tobacco	0.05 (0.03, 0.08)	4.67E-05	Adj.R.Squared	0.2401

Alcohol	-0.05 (-0.1, -0.01)	0.0211	Statistic	44.9761
Cocaine	-0.16 (-0.23, -0.08)	4.52E-05	Deg.Freedom	6,101
Income	0 (0, 0)	6.13E-07	P-Value	<2.2E-16
Tobacco: LM.Cannabis_x_Resin.THC_x_Daily.Interpol.	0.03 (0.03, 0.04)	<2.2E-16		
LM.Cannabis_x_Resin.THC_x_Daily.Interpol.: LM.Cannabis_x_Herb.THC_x_Daily.Interpol.	0.07 (0.06, 0.08)	<2.2E-16		
2 Lags				
Rate ~ Tobacco * LM.Cannabis_x_Resin.THC_x_Daily.Interpol. * LM.Cannabis_x_Herb.THC_x_Daily.Interpol. + Alcohol+ Amphetamines + Cocaine + Income				
Tobacco	0.05 (0.02, 0.08)	0.0008	Adj.R.Squared	0.3093
LM.Cannabis_x_Herb.THC_x_Daily.Interpol.	14.5 (2.72, 26.28)	0.0176	Statistic	24.3414
Income	0 (0, 0)	0.0014	Deg.Freedom	8,85
Tobacco: LM.Cannabis_x_Resin.THC_x_Daily.Interpol.	0.09 (0.06, 0.11)	2.66E-08	P-Value	<2.2E-16
Tobacco: LM.Cannabis_x_Herb.THC_x_Daily.Interpol.	-0.74 (-1.25, -0.24)	0.0049		
LM.Cannabis_x_Resin.THC_x_Daily.Interpol.: LM.Cannabis_x_Herb.THC_x_Daily.Interpol.	1.49 (0.17, 2.81)	0.0303		
Tobacco: LM.Cannabis_x_Resin.THC_x_Daily.Interpol.: LM.Cannabis_x_Herb.THC_x_Daily.Interpol.	-0.07 (-0.12, -0.01)	0.0189		

Table S12. Final Interactive Inverse Probability Weighted Panel Regression Models for VACTERL Syndrome

Parameters			Model Parameters	
**Term**	**Estimate (C.I.)**	**P-Value**	**Parameter**	**Value**
Additive				
(Rate ~ Tobacco + Alcohol + LM.Cannabis_x_Herb.THC_x_Daily.Interpol. + LM.Cannabis_x_Resin.THC_x_Daily.Interpol. + Daily.Interpol. + LM.Cannabis_x_Resin.THC + Amphetamines + Cocaine + Income)				
Alcohol	0.09 (0.03, 0.15)	0.0067	R.Squared	0.3946
LM.Cannabis_x_Herb.THC_x_Daily.Interpol.	-8.99 (-10.85, -7.13)	1.35E-15	Adj.R.Squared	0.3583
LM.Cannabis_x_Resin.THC_x_Daily.Interpol.	2.78 (2.11, 3.45)	1.52E-12	Statistic	18.0280
Herb	5.19 (1.72, 8.66)	0.0042	Deg.Freedom	6,100
Cocaine	0.7 (0.55, 0.85)	2.12E-14	P-Value	4.45E-14
Income	0 (0, 0)	0.0058		
Interactive				
*Rate ~ Tobacco * LM.Cannabis_x_Resin.THC_x_Daily.Interpol. + Daily.Interpol. + Alcohol + Amphetamines + Cocaine + Income*				
Daily.Interpol.	-56.8 (-79.34, -34.26)	3.33E-06	Adj.R.Squared	0.0578
Cocaine	0.91 (0.6, 1.23)	1.43E-07	Statistic	8.7463
Income	0 (0, 0)	0.0001	Deg.Freedom	4,102
Tobacco: LM.Cannabis_x_Resin.THC_x_Daily.Interpol.	0.03 (0.02, 0.05)	4.72E-05	P-Value	4.12E-06
1 Lag				
*Rate ~ Tobacco * Daily.Interpol. + LM.Cannabis_x_Herb.THC_x_Daily.Interpol. + Alcohol + Amphetamines + Cocaine + Income*				
Tobacco	0.05 (0.01, 0.08)	0.0115	Adj.R.Squared	0.2463
Daily.Interpol.	109.51 (20.01, 199)	0.0185	Statistic	4.2094
Cocaine	0.52 (0.24, 0.79)	0.0004	Deg.Freedom	4,91
LM.Cannabis_x_Herb.THC: LM.Cannabis_x_Resin.THC_x_Daily.Interpol.	-5.36 (-9.42, -1.3)	0.0113	P-Value	3.50E-03
2 Lags				
*Rate ~ Tobacco * LM.Cannabis_x_Herb.THC_x_Daily.Interpol. + Alcohol + Amphetamines + Daily.Interpol. + Cocaine + Income*				
Alcohol	0.16 (0.07, 0.24)	0.0005	Adj.R.Squared	0.1410
Income	0 (0, 0)	0.0041	Statistic	6.6789
			Deg.Freedom	2,82
			P-Value	2.10E-03
4 Lags				

Rate ~ Tobacco * LM.Cannabis_x_Herb.THC_x_Daily.Interpol. + Alcohol + Amphetamines + Cocaine + Income				
LM.Cannabis_x_Herb.THC_x_Daily.Interpol.	74.71 (32.72, 116.7)	0.0009	Adj.R.Squared	0.1734
Alcohol	0.21 (0.12, 0.3)	3.53E-05	Statistic	6.5315
Cocaine	0.34 (0.14, 0.53)	0.0013	Deg.Freedom	4,58
Tobacco: LM.Cannabis_x_Herb.THC_x_Daily.Interpol.	-3.06 (-4.77, -1.35)	0.0009	P-Value	2.17E-04
Additive Model Without Cannabis Terms				
Rate ~ Tobacco + Alcohol + Cocaine + Income-Tobacco-Alcohol-Income				
Cocaine	0.16 (0.05, 0.27)	0.0045	R.Squared	0.2829
			Adj.R.Squared	0.2761
			Statistic	8.4271
			Deg.Freedom	1,105
			P-Value	4.51E-03

Table S13. Final Interactive Inverse Probability Weighted Panel Regression Models for Foetal Alcohol Syndrome

Parameters			Model Parameters	
**Term**	**Estimate (C.I.)**	**P-Value**	**Parameter**	**Value**
Additive				
(Rate ~ Tobacco + Alcohol + LM.Cannabis_x_Herb.THC_x_Daily.Interpol. + LM.Cannabis_x_Resin.THC_x_Daily.Interpol. + Daily.Interpol. + LM.Cannabis_x_Resin.THC + Amphetamines + Cocaine + Income)				
Tobacco	0.02 (0, 0.04)	0.0179	Adj.R.Squared	0.2921
Alcohol	0.07 (0.03, 0.1)	0.0005	Statistic	24.6949
LM.Cannabis_x_Herb.THC_x_Daily.Interpol.	-0.59 (-0.73, -0.44)	7.52E-13	Deg.Freedom	6,115
LM.Cannabis_x_Resin.THC_x_Daily.Interpol.	0.69 (0.53, 0.86)	1.44E-13	P-Value	<2.2E-16
Daily.Interpol.	-6.76 (-10.44, -3.08)	0.0005		
Income	0 (0, 0)	1.39E-05		
Interactive				
*Rate ~ Tobacco * LM.Cannabis_x_Resin.THC_x_Daily.Interpol. * LM.Cannabis_x_Herb.THC_x_Daily.Interpol. + Herb + Alcohol + Daily.Interpol. + Amphetamines + Cocaine + Income*				
Tobacco	0.03 (0.01, 0.05)	0.0042	Adj.R.Squared	0.1773
LM.Cannabis_x_Resin.THC_x_Daily.Interpol.	2.81 (1.07, 4.55)	0.0020	Statistic	19.8698
Alcohol	0.05 (0.01, 0.09)	0.0115	Deg.Freedom	8,113
Cocaine	-0.12 (-0.18, -0.06)	9.77E-05	P-Value	<2.2E-16
Income	0 (0, 0)	1.11E-05		
Tobacco: LM.Cannabis_x_Resin.THC_x_Daily.Interpol.	-0.1 (-0.17, -0.03)	0.0074		
LM.Cannabis_x_Resin.THC_x_Daily.Interpol.: LM.Cannabis_x_Herb.THC_x_Daily.Interpol.	0.31 (0.12, 0.5)	0.0016		
Tobacco: LM.Cannabis_x_Resin.THC_x_Daily.Interpol.: LM.Cannabis_x_Herb.THC_x_Daily.Interpol.	-0.01 (-0.02, 0)	0.0046		
2 Lags				
*Rate ~ Tobacco * LM.Cannabis_x_Herb.THC_x_Daily.Interpol. * LM.Cannabis_x_Resin.THC_x_Daily.Interpol. + Herb + Alcohol + Daily.Interpol. + Amphetamines + Cocaine + Income*				
Tobacco	0.04 (0.02, 0.06)	0.0002	Adj.R.Squared	0.1446
LM.Cannabis_x_Herb.THC_x_Daily.Interpol.	-9.71 (-13.45, -5.97)	2.25E-06	Statistic	23.5455
LM.Cannabis_x_Resin.THC_x_Daily.Interpol.	11.6 (7.37, 15.83)	6.97E-07	Deg.Freedom	9,84
Herb	2.83 (0.81, 4.85)	0.0070	P-Value	<2.2E-16
Alcohol	0.05 (0.01, 0.08)	0.0126		
Amphetamines	-0.19 (-0.29, -0.09)	0.0002		
Income	0 (0, 0)	0.0012		
Tobacco: LM.Cannabis_x_Herb.THC_x_Daily.Interpol.	0.36 (0.21, 0.52)	1.31E-05		
Tobacco: LM.Cannabis_x_Resin.THC_x_Daily.Interpol.	-0.44 (-0.61, -0.26)	3.47E-06		

Table S14. Final Interactive Inverse Probability Weighted Panel Regression Models for Situs Inversus

Parameters			Model Parameters	
**Term**	**Estimate (C.I.)**	**P-Value**	**Parameter**	**Value**
Additive				
(Rate ~ Tobacco + Alcohol + LM.Cannabis_x_Herb.THC_x_Daily.Interpol. + LM.Cannabis_x_Resin.THC_x_Daily.Interpol. + Daily.Interpol. + LM.Cannabis_x_Resin.THC + Amphetamines + Cocaine + Income)				
LM.Cannabis_x_Herb.THC_x_Daily.Interpol.	0.66 (0.23, 1.09)	0.0034	Adj.R.Squared	0.3120
LM.Cannabis_x_Resin.THC_x_Daily.Interpol.	-0.78 (-1.27, -0.28)	0.0026	Statistic	15.7351
LM.Cannabis_x_Herb.THC	9.82 (5.78, 13.86)	5.44E-06	Deg.Freedom	6,115
Amphetamines	-0.15 (-0.27, -0.03)	0.014758	P-Value	3.97E-13
Cocaine	-0.1 (-0.2, -0.01)	0.0377		
Income	0 (0, 0)	1.32E-10		
Interactive				
Rate ~ Tobacco * Resin * LM.Cannabis_x_Resin.THC + LM.Cannabis_x_Resin.THC_x_Daily.Interpol. + Alcohol + LM.Cannabis_x_Herb.THC_x_Daily.Interpol. + Amphetamines + Cocaine + Income				
Resin	-4.99 (-8.99, -0.99)	0.0164	Adj.R.Squared	0.3941
LM.Cannabis_x_Herb.THC_x_Daily.Interpol.	2.23 (0.67, 3.79)	0.0061	Statistic	87.5252
Amphetamines	-0.21 (-0.39, -0.02)	0.0357	Deg.Freedom	7,99
Cocaine	0.38 (0.16, 0.61)	0.0013	P-Value	3.85E-08
Income	0 (0, 0)	0.0037		
Tobacco: Resin	0.41 (0.23, 0.58)	1.26E-05		
Tobacco: LM.Cannabis_x_Resin.THC	-0.15 (-0.24, -0.06)	0.0012		
2 Lags				
Rate ~ Tobacco * LM.Cannabis_x_Herb.THC_x_Daily.Interpol. * LM.Cannabis_x_Resin.THC_x_Daily.Interpol. + LM.Cannabis_x_Herb.THC + Alcohol + Daily.Interpol. + Amphetamines + Cocaine + Income				
LM.Cannabis_x_Herb.THC_x_Daily.Interpol.	16.19 (10.6, 21.78)	1.88E-07	Adj.R.Squared	0.0558
LM.Cannabis_x_Resin.THC_x_Daily.Interpol.	-14.82 (-21.37, -8.27)	2.77E-05	Statistic	14.6021
LM.Cannabis_x_Herb.THC	13.39 (4.82, 21.97)	0.0030	Deg.Freedom	8,85
Alcohol	-0.11 (-0.15, -0.07)	9.84E-07	P-Value	3.28E-13
Daily.Interpol.	22.17 (6.9, 37.43)	0.0056		
Tobacco: LM.Cannabis_x_Herb.THC_x_Daily.Interpol.	-0.86 (-1.12, -0.6)	7.01E-09		
Tobacco: LM.Cannabis_x_Resin.THC_x_Daily.Interpol.	0.62 (0.35, 0.88)	1.36E-05		
Tobacco: LM.Cannabis_x_Herb.THC_x_Daily.Interpol.: LM.Cannabis_x_Resin.THC_x_Daily.Interpol.	-0.02 (-0.03, -0.01)	0.0004		

Table S15. Final Interactive Inverse Probability Weighted Panel Regression Models for Lateralization Anomalies

Parameters			Model Parameters	
**Term**	**Estimate (C.I.)**	**P-Value**	**Parameter**	**Value**
Additive				
(Rate ~ Tobacco + Alcohol + LM.Cannabis_x_Herb.THC_x_Daily.Interpol. + LM.Cannabis_x_Resin.THC_x_Daily.Interpol. + Daily.Interpol. + LM.Cannabis_x_Resin.THC + Amphetamines + Cocaine + Income)				
LM.Cannabis_x_Resin.THC_x_Daily.Interpol.	-1.74 (-2.83, -0.65)	0.0023	Adj.R.Squared	0.2783
Resin	2.63 (1.24, 4.01)	0.0003	Statistic	10.6103
LM.Cannabis_x_Herb.THC_x_Daily.Interpol.	3.53 (1.19, 5.88)	0.0039	Deg.Freedom	4,102
Cocaine	0.27 (0.1, 0.44)	0.0025	P-Value	3.15E-07
Interactive				
Rate ~ Tobacco * Resin * LM.Cannabis_x_Resin.THC + LM.Cannabis_x_Resin.THC_x_Daily.Interpol. + Alcohol + LM.Cannabis_x_Herb.THC_x_Daily.Interpol. + Amphetamines + Cocaine + Income				
Resin	-4.99 (-8.99, -0.99)	0.0164	Adj.R.Squared	0.3941
LM.Cannabis_x_Herb.THC_x_Daily.Interpol.	2.23 (0.67, 3.79)	0.0061	Statistic	8.5252
Amphetamines	-0.21 (-0.39, -0.02)	0.0357	Deg.Freedom	7,99
Cocaine	0.38 (0.16, 0.61)	0.0013	P-Value	3.84E-08
Income	0 (0, 0)	0.0037		
Tobacco: Resin	0.41 (0.23, 0.58)	1.26E-05		
Tobacco: LM.Cannabis_x_Resin.THC	-0.15 (-0.24, -0.06)	0.0012		
2 Lags				
Rate ~ Tobacco * LM.Cannabis_x_Herb.THC_x_Daily.Interpol. + LM.Cannabis_x_Resin.THC * Resin + LM.Cannabis_x_Resin.THC_x_Daily.Interpol. + Alcohol + Amphetamines + Cocaine + Income				
Resin	2.39 (1.41, 3.37)	7.39E-06	Adj.R.Squared	0.1396
Cocaine	0.39 (0.26, 0.53)	3.18E-07	Statistic	11.5721
LM.Cannabis_x_Resin.THC: Resin	-7.12 (-11.42, -2.83)	0.0017	Deg.Freedom	3.81
			P-Value	2.17E-06

Table S16. Final Interactive Inverse Probability Weighted Panel Regression Models for Teratogenic Syndromes

Parameters			Model Parameters	
erm	Estimate (C.I.)	P-Value	Parameter	Value
Additive				
(Rate ~ Tobacco + Alcohol + LM.Cannabis_x_Herb.THC_x_Daily.Interpol. + LM.Cannabis_x_Resin.THC_x_Daily.Interpol. + Daily.Interpol. + LM.Cannabis_x_Resin.THC + Amphetamines + Cocaine + Income)				
Tobacco	0.08 (0.04, 0.13)	0.0008	R.Squared	0.4089
Alcohol	0.25 (0.16, 0.35)	3.66E-07	Adj.R.Squared	0.3614
LM.Cannabis_x_Herb.THC_x_Daily.Interpol.	-3.29 (-3.87, -2.71)	<2.2E-16	Statistic	30.9959
LM.Cannabis_x_Resin.THC_x_Daily.Interpol.	3.88 (3.21, 4.55)	<2.2E-16	Deg.Freedom	9,112
Herb	-5.85 (-9.91, -1.79)	0.0056	P-Value	<2.2E-16
Daily.Interpol.	-106 (-128.15, -83.85)	9.44E-16		
Amphetamines	-0.42 (-0.63, -0.21)	0.0001		
Cocaine	1.44 (1.11, 1.77)	1.16E-13		
Income	0 (0, 0)	9.29E-05		
Interactive				
Rate ~ Tobacco * LM.Cannabis_x_Herb.THC_x_Daily.Interpol. * LM.Cannabis_x_Resin.THC_x_Daily.Interpol. + Herb + Alcohol + Daily.Interpol. + Amphetamines + Cocaine + Income				
Tobacco	0.07 (0.03, 0.11)	0.0006	Adj.R.Squared	0.4164
LM.Cannabis_x_Herb.THC_x_Daily.Interpol.	6.17 (4.07, 8.27)	6.64E-08	Statistic	36.9812
Alcohol	0.31 (0.22, 0.39)	1.03E-10	Deg.Freedom	10,111
Daily.Interpol.	-48.3 (-76.33, -20.27)	0.0010	P-Value	<2.2E-16
Amphetamines	-0.44 (-0.63, -0.25)	1.33E-05		
Cocaine	1.23 (0.91, 1.55)	1.87E-11		
Income	0 (0, 0)	6.22E-06		
Tobacco: LM.Cannabis_x_Herb.THC_x_Daily.Interpol.	-0.69 (-0.88, -0.5)	5.84E-11		
Tobacco: LM.Cannabis_x_Resin.THC_x_Daily.Interpol.	0.22 (0.19, 0.25)	<2.2E-16		
Tobacco: LM.Cannabis_x_Herb.THC_x_Daily.Interpol.: LM.Cannabis_x_Resin.THC_x_Daily.Interpol.	-0.02 (-0.03, -0.02)	6.01E-08		
2 Lags				
Rate ~ Tobacco * LM.Cannabis_x_Resin.THC_x_Daily.Interpol. + LM.Cannabis_x_Herb.THC_x_Daily.Interpol. + Alcohol + Herb + Daily.Interpol. + Amphetamines + Cocaine + Income				
Tobacco	0.09 (0.02, 0.15)	0.00991	R.Squared	0.2902
Daily.Interpol.	3.24 (2.26, 4.22)	5.52E-09	Adj.R.Squared	0.2413

LM.Cannabis_x_Herb.THC_x_Daily.Interpol.	-2.7 (-3.57, -1.83)	3.59E-08	Statistic	14.2913
Alcohol	0.14 (0.02, 0.26)	0.02882	Deg.Freedom	6,87
Herb	-9.35 (-15.97, -2.73)	0.00687	P-Value	2.87E-11
Income	0 (0, 0)	3.24E-07		
Additive Model without Cannabis Terms				
Rate ~ Tobacco + Alcohol + Amphetamines + Cocaine + Income-Amphetamines-Income				
Tobacco	-0.08 (-0.12, -0.04)	7.26E-05	R.Squared	0.3041
Alcohol	0.25 (0.13, 0.36)	6.84E-05	Adj.R.Squared	0.2864
Cocaine	0.37 (0.2, 0.54)	3.89E-05	Statistic	13.4408
			Deg.Freedom	3,118
			P-Value	1.32E-07

Table S17. All E-Values

No.	Anomaly	Regression	Model Type	Term	P-Value	E-Value Estimate	Lower Bound E-Value
1	VACTERL	Spatial	4 Lags	Daily.Interpol.	6.81E-12	Infinity	Infinity
2	VACTERL	Spatial	6 Lags	Daily.Interpol.	8.10E-10	Infinity	Infinity
3	VACTERL	Spatial	2 Lags	Daily.Interpol.	6.56E-06	Infinity	6.52E+138
4	VACTERL	Spatial	Interactive	Daily.Interpol.	0.0006	Infinity	2.53E+67
5	VACTERL	Panel	4 Lags	LM.Cannabis_x_Herb.THC_x_Daily.Interpol.	0.0009	5.93E+38	1.56E+17
6	FAS	Panel	2 Lags	LM.Cannabis_x_Resin.THC_x_Daily.Interpol.	6.97E-07	1.00E+18	3.56E+11
7	VACTERL	Panel	1 Lag	Daily.Interpol.	0.0185	3.32E+59	1.67E+11
8	Situs Inversus	Panel	2 Lags	LM.Cannabis_x_Herb.THC_x_Daily.Interpol.	1.88E-07	1.38E+16	4.79E+10
9	Situs Inversus	Panel	2 Lags	Daily.Interpol.	0.0056	9.76E+21	1.20E+07
10	Situs Inversus	Panel	Additive	LM.Cannabis_x_Herb.THC	5.44E-06	7.97E+10	6.73E+05
11	Teratogenic Syndromes	Spatial	Interactive	Herb	0.0002	7.54E+17	2.85E+05
12	VACTERL	Spatial	4 Lags	LM.Cannabis_x_Herb.THC	0.0022	1.65E+11	1.76E+04
13	Situs Inversus	Panel	2 Lags	LM.Cannabis_x_Herb.THC	0.0030	2.55E+13	1.07E+04
14	All Anomalies	Panel	2 Lags	LM.Cannabis_x_Herb.THC_x_Daily.Interpol.	0.0176	4.47E+15	1.76E+03
15	All Anomalies	Spatial	Interactive	Herb	8.09E-06	2.87E+04	1.08E+03
16	Teratogenic Syndromes	Panel	Interactive	LM.Cannabis_x_Herb.THC_x_Daily.Interpol.	6.64E-08	1.86E+04	850.04
17	VACTERL	Spatial	Additive	Daily.Interpol.	0.0205	2.61E+16	647.17
18	All Anomalies	Spatial	Additive	LM.Cannabis_x_Resin.THC	8.96E-06	8.49E+03	213.85
19	Teratogenic Syndromes	Spatial	Additive	Herb	0.0035	1.77E+05	155.66
20	Teratogenic Syndromes	Panel	Additive	LM.Cannabis_x_Resin.THC_x_Daily.Interpol.	<2.2E-16	356.29	145.49
21	Situs Inversus	Spatial	Additive	Herb	0.0019	9.61E+04	107.71
22	Situs Inversus	Spatial	Interactive	Herb	0.0019	9.61E+04	107.71

23	Teratogenic Syndromes	Spatial	2 Lags	Herb	0.0137	2.28E+05	107.43
24	All Anomalies	Panel	Interactive	LM.Cannabis_x_Herb.THC_x_Daily.Interpol.	<2.2E-16	212.22	106.93
25	FAS	Spatial	Additive	Herb	0.0004	1.41E+04	104.35
26	FAS	Spatial	Interactive	Herb	0.0004	1.41E+04	104.35
27	VACTERL	Panel	Additive	LM.Cannabis_x_Resin.THC_x_Daily.Interpol.	1.52E-12	230.82	72.72
28	FAS	Panel	Interactive	LM.Cannabis_x_Resin.THC_x_Daily.Interpol.	0.0020	1.57E+04	61.02
29	VACTERL	Panel	Additive	Herb	0.0042	1.42E+04	37.93
30	Lateralization	Spatial	Additive	LM.Cannabis_x_Resin.THC	0.0006	1.83E+03	36.69
31	Lateralization	Spatial	Interactive	LM.Cannabis_x_Resin.THC	0.0006	1.83E+03	36.69
32	FAS	Panel	2 Lags	Herb	0.0070	4.29E+04	36.32
33	Teratogenic Syndromes	Spatial	Additive	LM.Cannabis_x_Resin.THC_x_Daily.Interpol.	0.0003	2.91E+03	33.76
34	Teratogenic Syndromes	Spatial	Interactive	LM.Cannabis_x_Resin.THC_x_Daily.Interpol.	5.65E-05	2.63E+03	29.12
35	Teratogenic Syndromes	Spatial	2 Lags	LM.Cannabis_x_Resin.THC_x_Daily.Interpol.	0.0029	2.02E+05	21.03
36	Lateralization	Panel	Additive	Resin	0.0003	186.86	16.72
37	Teratogenic Syndromes	Panel	2 Lags	lag(LpmResinDailyInt, 2)	5.52E-09	39.30	15.60
38	Lateralization	Panel	Additive	LM.Cannabis_x_Herb.THC_x_Daily.Interpol.	0.0039	892.53	15.10
39	Lateralization	Panel	2 Lags	Resin	7.39E-06	50.49	13.06
40	Lateralization	Panel	Interactive	Tobacco: Resin	1.26E-05	75.09	10.63
41	FAS	Panel	Additive	LM.Cannabis_x_Resin.THC_x_Daily.Interpol.	1.44E-13	17.04	10.05
42	Lateralization	Panel	Interactive	LM.Cannabis_x_Herb.THC_x_Daily.Interpol.	0.0061	263.18	9.83
43	Situs Inversus	Panel	Interactive	LM.Cannabis_x_Herb.THC_x_Daily.Interpol.	0.0061	50.05	4.74
44	All Anomalies	Panel	Interactive	LM.Cannabis_x_Resin.THC_x_Daily.Interpol.: LM.Cannabis_x_Herb.THC_x_Daily.Interpol.	7.81E-15	5.67	4.29
45	Situs Inversus	Spatial	1 Lags	Herb	0.0299	1.05E+04	4.10
46	Situs Inversus	Panel	2 Lags	Tobacco: LM.Cannabis_x_Resin.THC_x_Daily.Interpol.	1.36E-05	7.48	3.88
47	FAS	Panel	2 Lags	Tobacco: LM.Cannabis_x_Herb.THC_x_Daily.Interpol.	1.31E-05	6.65	3.61

48	All Anomalies	Spatial	2 Lags	LM.Cannabis_x_Resin.THC	0.0037	18.72	3.60
49	Lateralization	Spatial	2 Lags	LM.Cannabis_x_Resin.THC	0.0134	54.61	3.41
50	Situs Inversus	Panel	Additive	LM.Cannabis_x_Herb.THC_x_Daily.Interpol.	0.0034	8.35	2.86
51	All Anomalies	Panel	2 Lags	LM.Cannabis_x_Resin.THC_x_Daily.Interpol.: LM.Cannabis_x_Herb.THC_x_Daily.Interpol.	0.0303	73.47	2.36
52	FAS	Panel	Interactive	LM.Cannabis_x_Resin.THC_x_Daily.Interpol.: LM.Cannabis_x_Herb.THC_x_Daily.Interpol.	0.0016	4.78	2.31
53	Situs Inversus	Panel	Interactive	Tobacco: Resin	1.26E-05	3.01	2.15
54	Teratogenic Syndromes	Panel	Interactive	Tobacco: LM.Cannabis_x_Resin.THC_x_Daily.Interpol.	<2.2E-16	2.10	1.95
55	All Anomalies	Panel	1 Lag	LM.Cannabis_x_Resin.THC_x_Daily.Interpol.: LM.Cannabis_x_Herb.THC_x_Daily.Interpol.	<2.2E-16	1.69	1.62
56	All Anomalies	Panel	2 Lags	Tobacco: LM.Cannabis_x_Resin.THC_x_Daily.Interpol.	2.66E-08	1.77	1.57
57	FAS	Spatial	2 Lags	LM.Cannabis_x_Resin.THC_x_Daily.Interpol.	0.0034	23.51	1.53
58	All Anomalies	Panel	Additive	LM.Cannabis_x_Resin.THC_x_Daily.Interpol.	0.0001	1.75	1.45
59	All Anomalies	Panel	1 Lag	Tobacco: LM.Cannabis_x_Resin.THC_x_Daily.Interpol.	<2.2E-16	1.42	1.38
60	All Anomalies	Spatial	Interactive	LM.Cannabis_x_Herb.THC_x_Daily.Interpol.	2.23E-02	1.92	1.34
61	Lateralization	Spatial	Additive	LM.Cannabis_x_Herb.THC_x_Daily.Interpol.	0.0476	149.75	1.29
62	Lateralization	Spatial	Interactive	LM.Cannabis_x_Herb.THC_x_Daily.Interpol.	0.0476	149.75	1.29
63	All Anomalies	Spatial	2 Lags	LM.Cannabis_x_Resin.THC_x_Daily.Interpol.	0.0198	1.65	1.20
64	VACTERL	Panel	Interactive	Tobacco: LM.Cannabis_x_Resin.THC_x_Daily.Interpol.	4.72E-05	1.26	1.18

SUPPLEMENTARY FIGURES

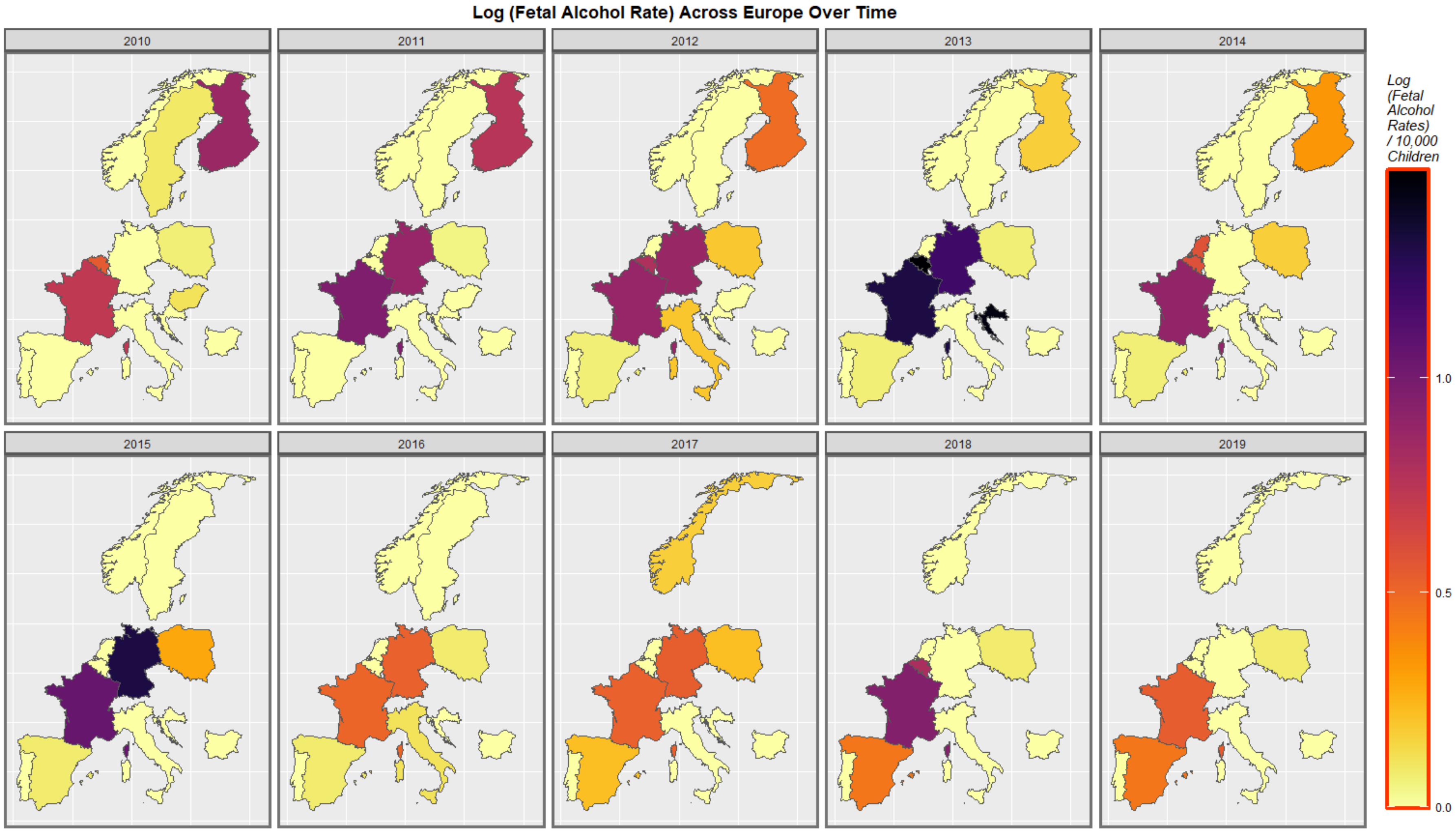

Figure S1. Sequential map-graph of the log rate of Foetal Alcohol Syndrome over time in selected European nations.

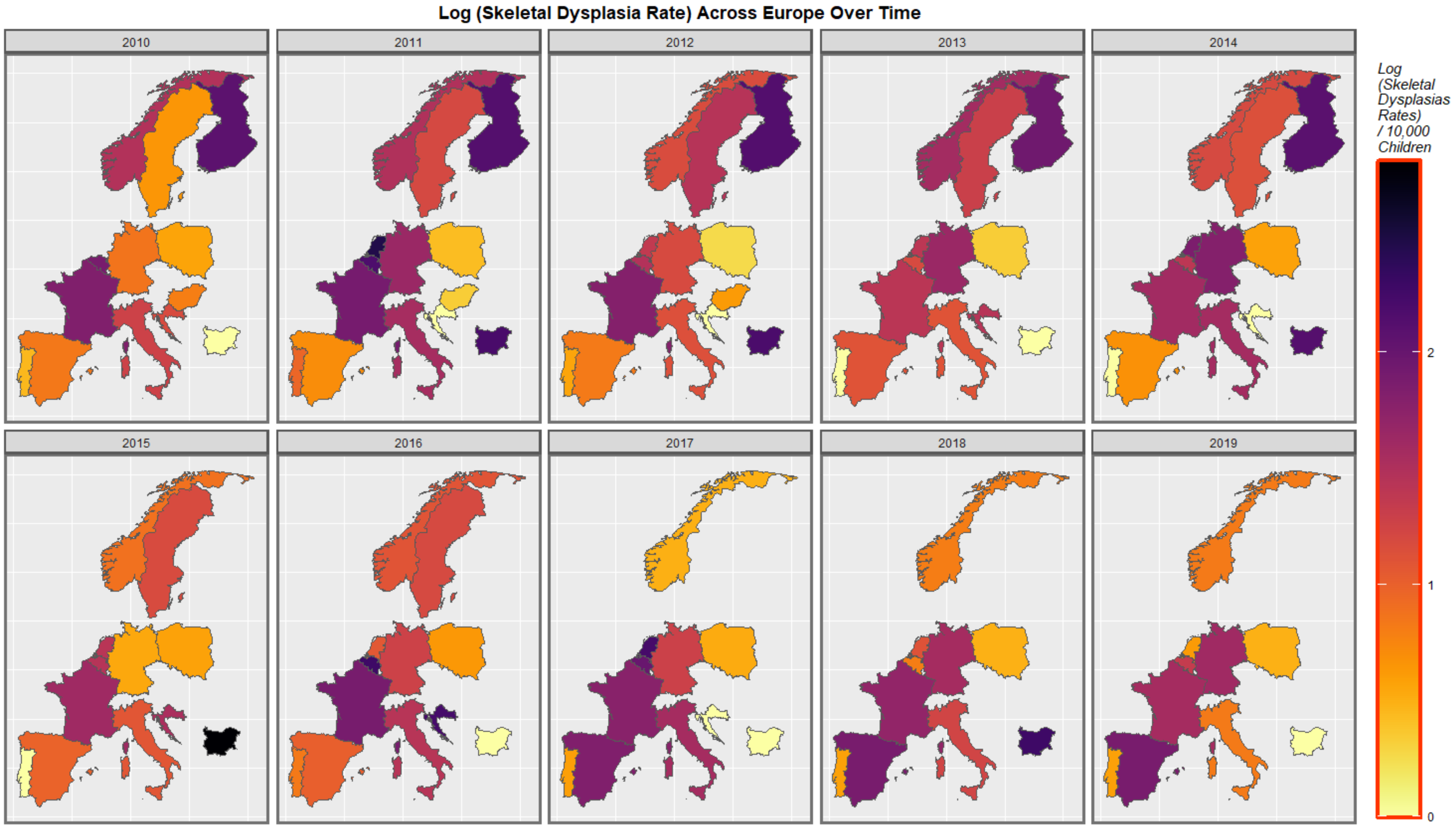

Figure S2. Sequential map-graph of the log rate of Skeletal Dysplasia over time in selected European nations.

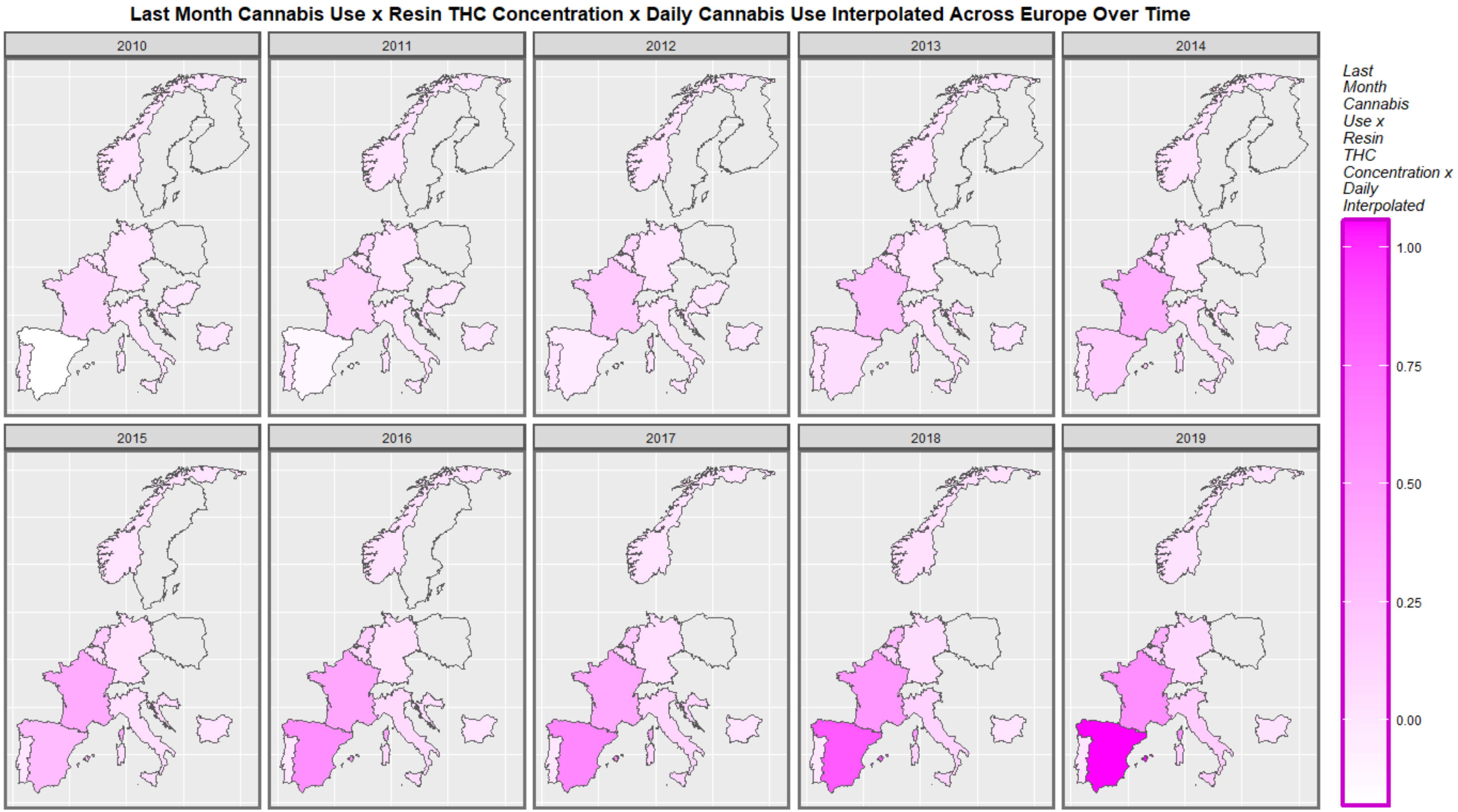

Figure S3. Sequential map-graph of the log rate of past month cannabis use x cannabis resin THC concentration x daily cannabis use interpolated over time in selected European nations.

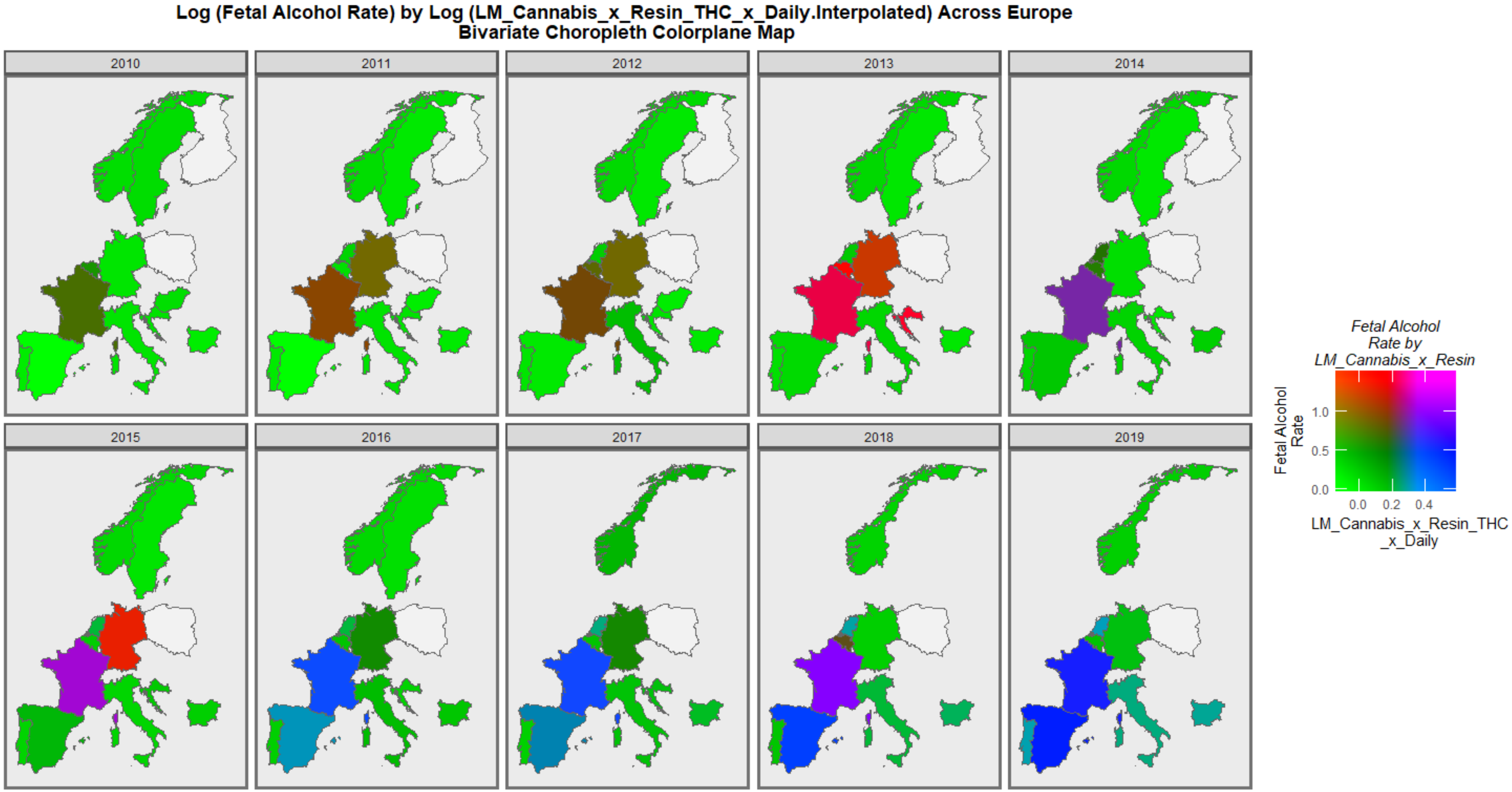

Figure S4. Bivariate colorplane sequential map-graph of the log rate of Foetal Alcohol Syndrome by last month cannabis use : cannabis resin THC concentration : daily cannabis use interpolated in selected European nations.

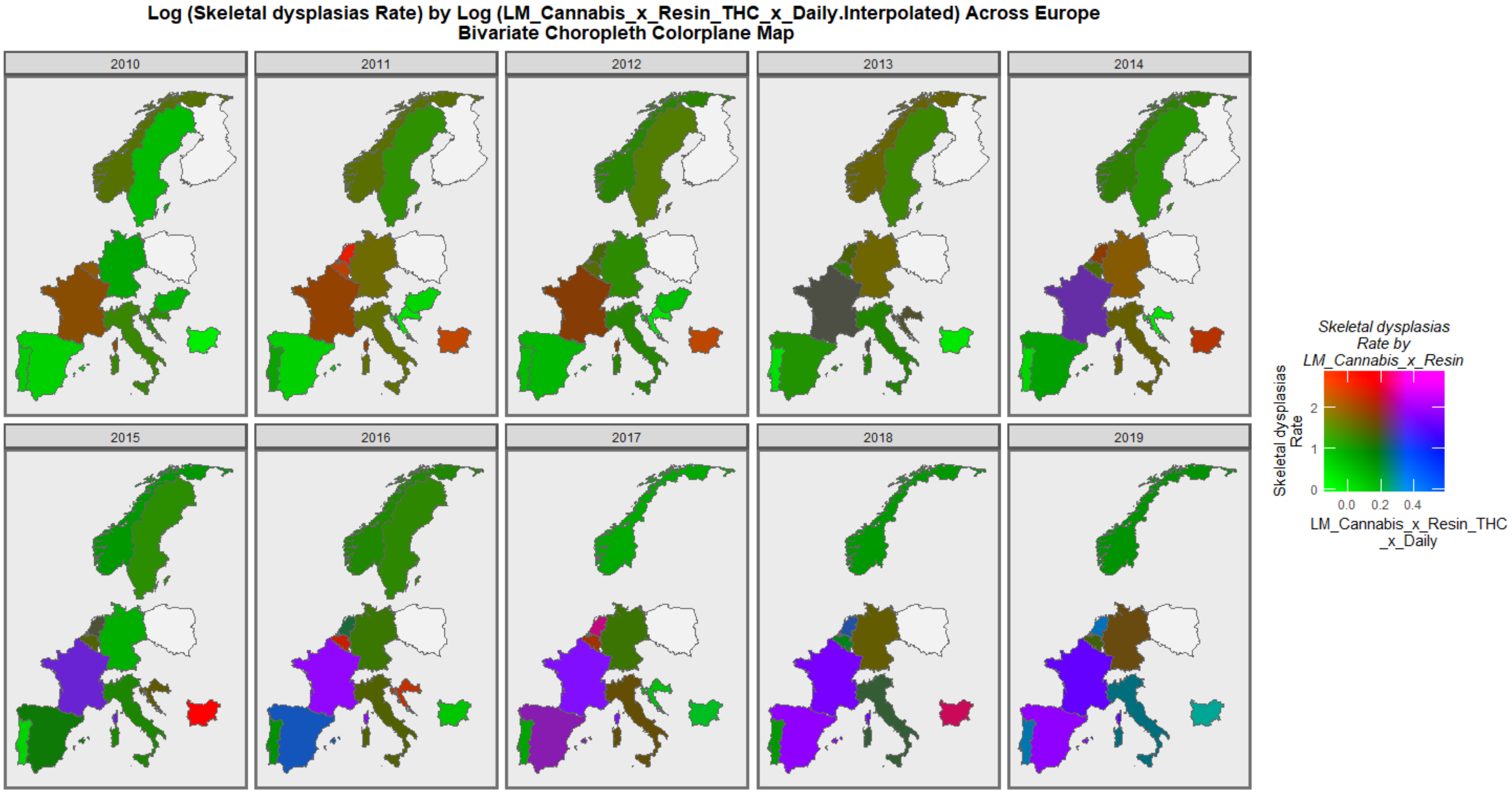

Figure S5. Bivariate colorplane sequential map-graph of the log rate of Skeletal Dysplasia by last month cannabis use : cannabis resin THC concentration : daily cannabis use interpolated in selected European nations.

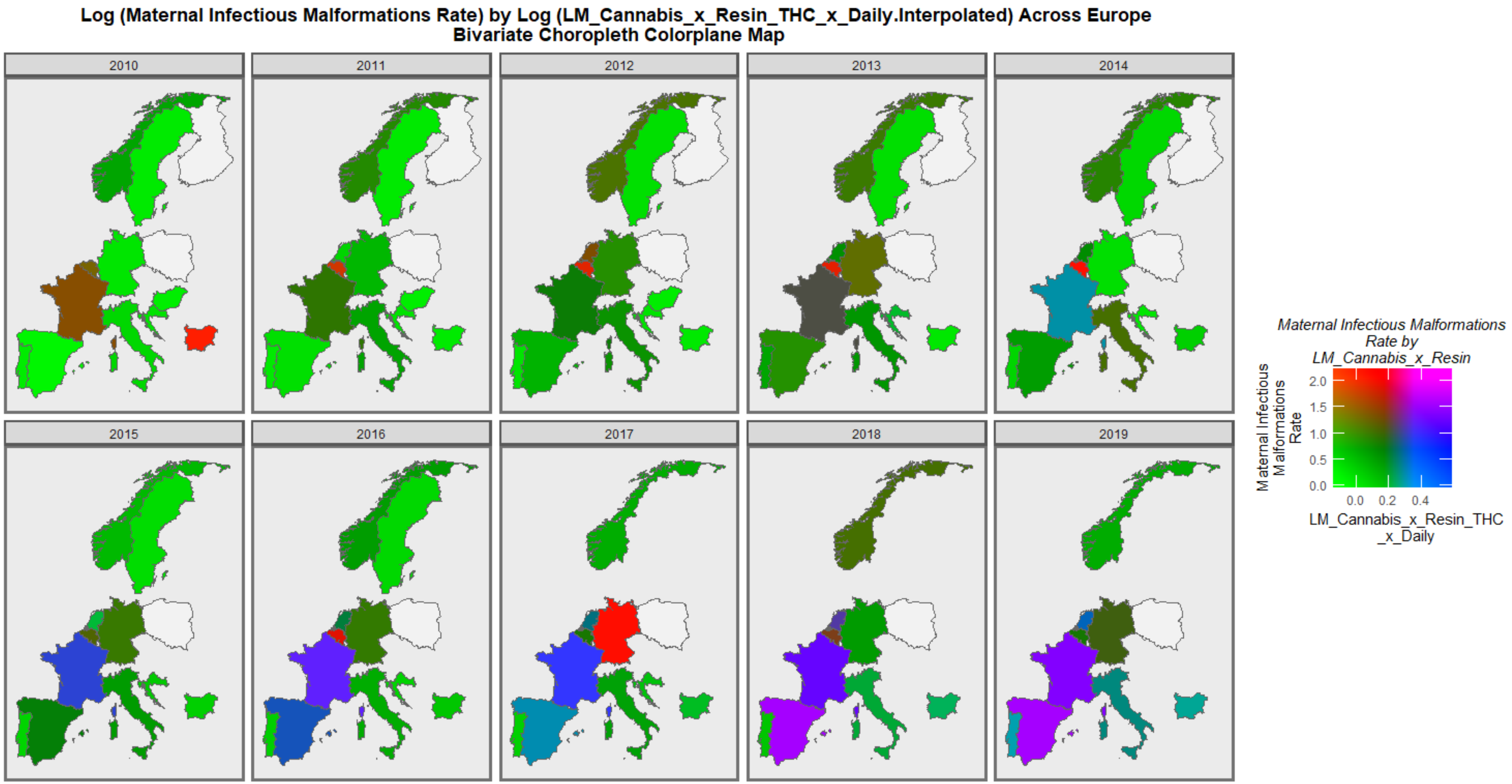

Figure S6.: Bivariate colorplane sequential map-graph of the log rate of Maternal Infections Anomalies by last month cannabis use : cannabis resin THC concentration : daily cannabis use interpolated in selected European nations.

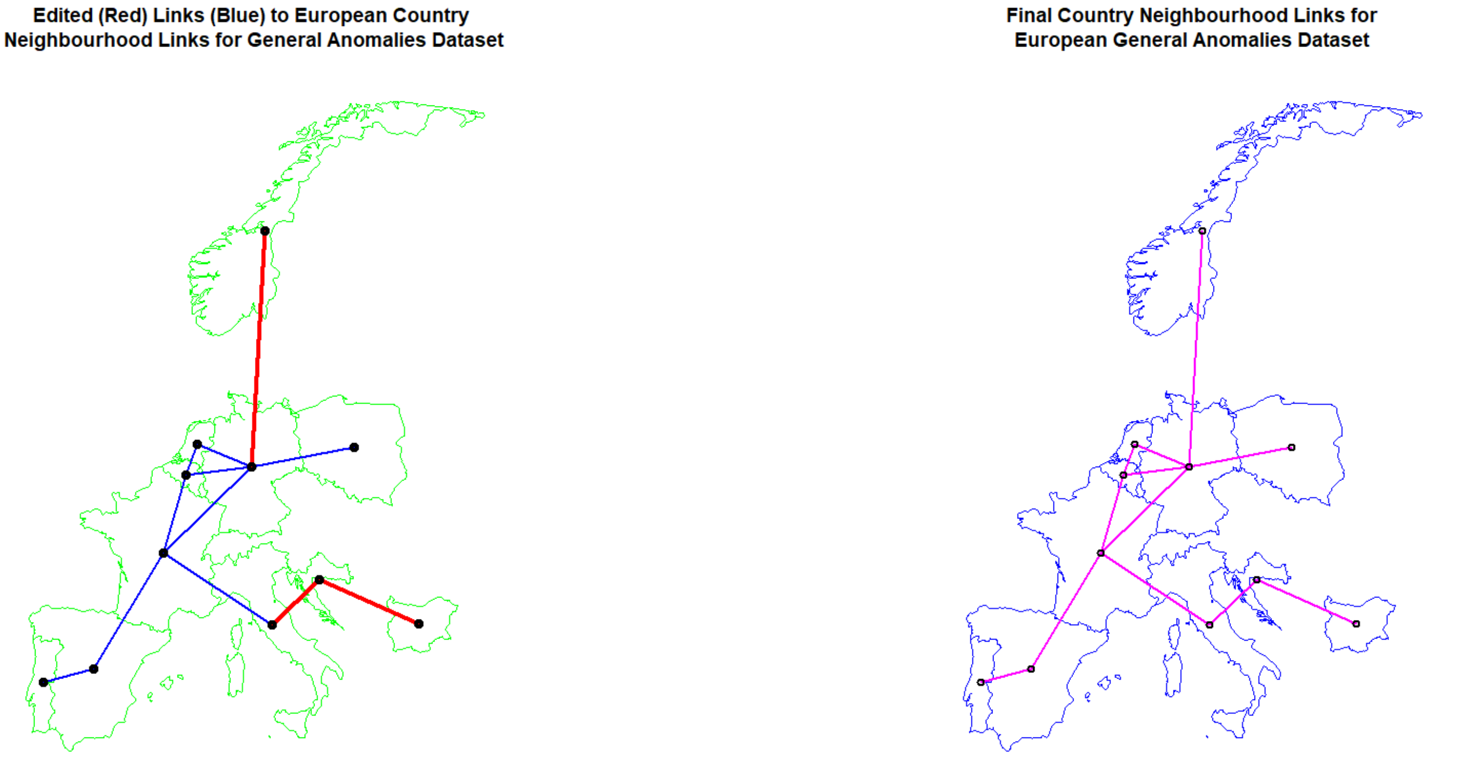

Figure S7. International geospatial links (A) raw (in blue) and edited (in red) and (B) final (in pink) used to derive the sparse spatial weights matrix for spatial regression.
